# Neoadjuvant pepinemab in combination with nivolumab and/or ipilimumab in resectable metastatic melanoma: a pilot biomarker-driven trial

**DOI:** 10.1038/s41467-026-78231-3

**Published:** 2026-10-06

**Authors:** Ayana T. Ruffin, Crystal L. Mallow, Brian M. Olson, Jacklyn Hammons, Brenda Melendez, Manoj Chelvanambi, Elaine M. Gersz, Megen C. Wittling, Laurence P. Diggs, Christine Reilly, Jeffrey M. Switchenko, Alexander J. Lazar, Shannon K. Swisher, Jayden Kim, Brian Burns, Agnes Harutyunyan, Terrence L. Fisher, Keith A. Delman, Melinda L. Yushak, Douglas C. Parker, David Lawson, Jennifer A. Wargo, Maurice Zauderer, Elizabeth E. Evans, Chrystal M. Paulos, Michael C. Lowe, Gregory B. Lesinski

**Affiliations:** 1 Department of Surgery, Winship Cancer Institute of Emory University, Atlanta, GA USA; 2 Vaccinex, Inc, Rochester, NY USA; 3 Department of Hematology and Medical Oncology, Winship Cancer Institute of Emory University Atlanta, Atlanta, GA USA; 4 MD Anderson Cancer Center, Houston, TX USA; 5 Department of Biostatistics and Bioinformatics, Rollins School of Public Health of Emory University Atlanta, Atlanta, GA USA; 6 Winship Cancer Institute of Emory University, Atlanta, GA USA; 7 Department of Pathology, Winship Cancer Institute of Emory University, Atlanta, GA USA; 8 Department of Microbiology and Immunology, Winship Cancer Institute of Emory University, Atlanta, GA USA

**Keywords:** Tumour immunology, Melanoma

## Abstract

Neoadjuvant immune checkpoint inhibitors (ICIs) improve event-free survival in metastatic melanoma, yet resistance and recurrence remain common. Here we evaluated pepinemab, a Semaphorin 4D (SEMA4D)–blocking antibody, in combination with anti-PD1 nivolumab and/or anti-CTLA4 ipilimumab in a pilot biomarker-driven neoadjuvant trial (NCT03769155) for patients with resectable metastatic melanoma. The primary endpoint was to determine if pepinemab combined with ICIs modulates phenotypic properties within the tumor microenvironment in a manner that enhances T effector cell infiltration. Secondary endpoints included safety and tolerability, pathological response rates and radiographical response. Patients received two neoadjuvant doses followed by curative-intent surgery and one year of adjuvant nivolumab. Pepinemab-containing regimens demonstrated manageable toxicity profiles and did not delay or preclude curative-intent surgical resection in the 38 patients treated. The trial met its primary endpoint, as enrichment of transcripts associated with activated dendritic cells and immune recruitment pathways was observed in tumors from patients receiving pepinemab-containing regimens. Further, immune remodeling was evident by formation of mature lymphoid aggregates resembling tertiary lymphoid structures that contained clonally expanded T cell receptor repertoires. While this study was not powered to assess clinical efficacy, our findings do support further evaluation of SEMA4D blockade as a strategy to modulate the tumor microenvironment and potentially augment immunotherapy in melanoma and other solid malignancies.

## Abstract (web summary)

Preclinical studies have suggested that blocking semaphorin 4D (SEMA4D) in combination with immune checkpoint inhibitors can enhance anti-tumor immunity. Here the authors report the results of a pilot biomarker-driven neoadjuvant trial of the SEMA4D blocking antibody pepinemab in combination with nivolumab (anti-PD-1) and/or ipilimumab (anti CTLA-4) in patients with resectable metastatic melanoma.

## Introduction

The management of patients with solid tumors is rapidly evolving with integration of neoadjuvant immunotherapy. This shift in surgical practice to incorporate immunotherapy in the pre-operative setting is gaining traction across melanoma, lung, oral, rectal, and breast cancers, with its application poised to expand to other tumor types as emerging clinical trial data continue to shape the field. Several clinical trials (*SWOG1801, NADINA*, NCT02519322) in melanoma have demonstrated that patients receiving neoadjuvant-adjuvant ICI (anti-PD-1, anti CTLA-4, and anti-LAG-3) had significantly longer event free survival than those receiving adjuvant therapy alone^1–3^. Despite these advances^1–6^, some patients who initially benefit from antibodies that block inhibitory checkpoint molecules still progress, particularly those who do not experience a major pathologic response (mPR)^4^. Proposed mediators of tumor-extrinsic resistance to ICIs include impaired antigen presentation and enrichment of suppressive myeloid cells, including myeloid-derived suppressor cells (MDSCs) and tumor associated macrophages within the tumor microenvironment (TME)^7–9^. These data highlight the significance of ICI resistance as a pressing clinical issue and the potential benefit of adapting complementary targets distinct from existing immune checkpoint therapies. While neoadjuvant ICI therapy has improved outcomes in melanoma patients^1–3^, additional approaches are needed to reduce recurrence risk.

Semaphorin 4D (SEMA4D) is a membrane-bound and secreted glycoprotein known for its role in regulating cell motility in the nervous system and cell to cell interactions of immune cells^10^. SEMA4D is present on tumors, resting T cells and, to a lesser extent, on B cells, monocytes, and dendritic cells (DCs)^11^ while its expression is upregulated upon cell activation. The receptors that bind SEMA4D—plexin B1, plexin B2 (PLXNB1/2) and lower affinity receptor CD72—are expressed on tumors, B cells and antigen presenting cells of the myeloid lineage^11–14^. In the TME, interactions between PLXNB1/2 and SEMA4D drive tumor migration, proliferation, and invasion, contributing to disease progression^13,15,16^. Preclinical studies revealed that blocking SEMA4D in combination with ICIs enhances intratumoral T cell infiltration and is associated with increased IFN-γ and TNF-ɑ production. Blocking SEMA4D limits chemokine-driven recruitment of MDSCs while reprogramming myeloid populations to enhance antigen presentation, reduce immunosuppressive macrophages, and downregulate arginase expression, thereby bolstering T cell expansion^17^. Consistent with this mechanism, SEMA4D blockade enhances the efficacy of ICIs across diverse tumor models, suggesting broad therapeutic potential across disease contexts^18,19^.

Pepinemab (VX15/2503) is a humanized IgG4 SEMA4D-blocking monoclonal antibody^18^ currently under clinical investigation in patients with oncologic and neurodegenerative diseases^20,21^. Pepinemab is well tolerated as both a single agent^22^ and in combination with checkpoint inhibitors^23^.Treatment with pepinemab plus avelumab demonstrated antitumor activity in NSCLC patients who progressed on prior therapy with single-agent checkpoint inhibitors, as well as in immunotherapy naïve patients with challenging PD-L1 negative or low tumors^23^. Overall, while these findings suggest that targeting SEMA4D-PLXNB1/2 interactions could remodel the TME and enhance ICI efficacy, the specific impact of SEMA4D blockade in the neoadjuvant setting for human melanoma patients remains poorly defined.

We hypothesized that blockade of SEMA4D would complement ICI activity, not by simply increasing immune infiltration, but by remodeling the TME to support productive immune communication through distinct and complementary mechanisms. Here, we report results from a pilot biomarker-driven study (NCT03769155) evaluating neoadjuvant pepinemab in combination with nivolumab and/or ipilimumab. Using comprehensive analyses of tumor tissue and peripheral blood, we show that SEMA4D blockade in combination with ICI is associated with coordinated myeloid and lymphoid remodeling, including the emergence of mature lymphoid aggregates (mLAs) resembling tertiary lymphoid structures (TLS). These findings support a model whereby SEMA4D blockade may complement ICI by promoting immune organization and provide rationale for further evaluation in larger randomized studies.

## Results

### Clinical characteristics and safety of neoadjuvant pepinemab-containing regimens

**Fig. 1:**
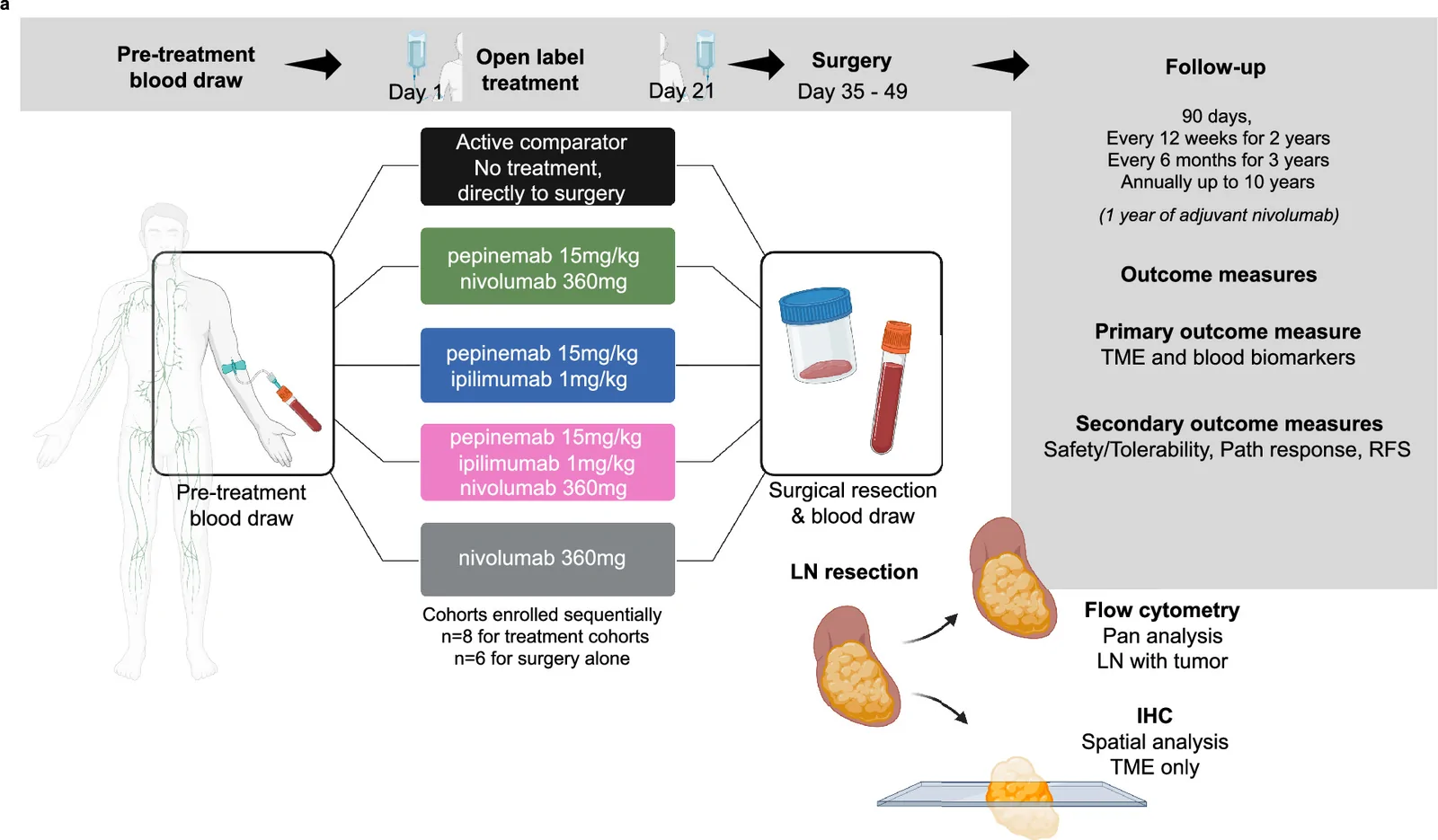
Clinical trial design. **a** Schematic of the pilot biomarker neoadjuvant trial evaluating pepinemab in combination with nivolumab and/or ipilimumab in resectable melanoma. Patients were enrolled sequentially into four treatment arms: Arm A, pepinemab plus nivolumab (*n* = 8); Arm B, pepinemab plus ipilimumab (*n* = 8); Arm C, pepinemab plus ipilimumab plus nivolumab (*n* = 8); and Arm D, nivolumab alone (*n* = 8). Patients received two treatment doses on days 1 and 21, followed by planned surgical resection between days 35 and 49. An active comparator cohort (Arm E, *n* = 6) underwent surgical resection without neoadjuvant immunotherapy within 30 days of consent. Peripheral blood was collected on days 1 and 21, and tumor-involved lymph nodes were obtained at the time of surgery. Follow-up assessments are shown. Primary objectives included characterization of the tumor microenvironment (TME) and peripheral blood biomarkers. Secondary objectives included safety, tolerability, pathologic response, and recurrence-free survival (RFS). Figure created in BioRender; Evans, E. (2026) https://BioRender.com/f86y400.

From December 2018 to September 2022, 38 patients with resectable metastatic melanoma were sequentially enrolled into four treatment arms (*n* = 8 patients per arm) or a surgery-only arm (*n* = 6). An overview of the study design is shown in Fig. 1. Two treatment doses were administered, the first on day 1 and the second on day 21, followed by surgery between days 35 and 49. Patients assigned to the surgery-only standard-of-care arm underwent resection within 30 days of consent. The study protocol can be found in Supplementary Information. The median age of treated patients was 60 years (range 20–83), and most patients were male (60.5%). Clinical staging included 15.8% stage IIIB, 44.7% stage IIIC, and 39.5% stage IIID disease. Additionally, 44.7% of patients presented with de novo disease, whereas 55.3% had undergone prior surgical management of their primary disease (Table 1).

**Table 1:** Clinical and demographic characteristics of melanoma patient cohort

Treatment Arm	Arm A		Arm B		Arm C		Arm D		Arm E		Total	
(Treatment regimens)	pepinemab + nivolumab		pepinemab + ipilimumab		pepinemab + ipilimumab + nivolumab		nivolumab		Control arm (upfront surgery with adjuvant therapy) [no neoadjuvant therapy]			
Subjects (*N*)	8	100.0%	8	100.0%	8	100.0%	8	100.0%	6	100.0%	38	100.0%
Included in assessment of efficacy	8	100.0%	8	100.0%	8	100.0%	8	100.0%	5	83.3%	37	97.4%
Age		Mean (range)		Mean (range)		Mean (range)		Mean (range)		Mean (range)		Mean (range)
Median (range)	59 (41–83)	62 (41–83)	56 (20–68)	51 (20–68)	57 (47–72)	59 (47–72)	59 (47–74)	59 (47–74)	71 (65–82)	72 (65–82)	60 (20–83)	60 (20–83)
Gender												
Male	4	50.0%	5	62.5%	5	62.5%	6	75.0%	3	50.0%	23	60.5%
Female	4	50.0%	3	37.5%	3	37.5%	2	25.0%	3	50.0%	15	39.5%
Total	8	100.0%	8	100.0%	8	100.0%	8	100.0%	6	100.0%	38	100.0%
Race												
Black or African American	0	0.0%	1	12.5%	0	0.0%	0	0.0%	0	0.0%	1	2.6%
White	8	100.0%	7	87.5%	8	100.0%	8	100.0%	6	100.0%	37	97.4%
Unknown or not reported	0	0.0%	0	0.0%	0	0.0%	0	0.0%	0	0.0%	0	0.0%
Total	8	100.0%	8	100.0%	8	100.0%	8	100.0%	6	100.0%	38	100.0%
Ethnicity												
Hispanic or Latino/a	0	0.0%	0	0.0%	0	0.0%	1	12.5%	0	0.0%	1	2.6%
Non-hispanic	0	0.0%	7	87.5%	6	75.0%	4	50.0%	6	100.0%	23	60.5%
Not hispanic, Latino/a, or Spanish Origin	8	100.0%	1	12.5%	1	12.5%	2	25.0%	0	0.0%	12	31.6%
Unknown or not reported	0	0.0%	0	0.0%	1	12.5%	1	12.5%	0	0.0%	2	5.3%
Total	8	100.0%	8	100.0%	8	100.0%	8	100.0%	6	100.0%	38	100.0%
ECOG PS												
0	5	62.5%	7	87.5%	7	87.5%	6	75.0%	4	66.7%	29	76.3%
1	3	37.5%	1	12.5%	1	12.5%	2	25.0%	2	33.3%	9	23.7%
Total	8	100.0%	8	100.0%	8	100.0%	8	100.0%	6	100.0%	38	100.0%
BRAF mutation Status												
Present	1	12.5%	4	50.0%	5	62.5%	5	62.5%	3	50.0%	18	47.4%
Absent	5	62.5%	2	25.0%	1	12.5%	3	37.5%	3	50.0%	14	36.8%
Unknown or not assessed	2	25.0%	2	25.0%	2	25.0%	0	0.0%	0	0.0%	6	15.8%
Total	8	100.0%	8	100.0%	8	100.0%	8	100.0%	6	100.0%	38	100.0%
Other mutation status												
Present	1	12.5%	1	12.5%	6	75.0%	4	50.0%	2	33.3%	14	36.8%
Absent	0	0.0%	1	12.5%	0	0.0%	2	25.0%	4	66.7%	7	18.4%
Unknown or not assessed	7	87.5%	6	75.0%	2	25.0%	2	25.0%	0	0.0%	17	44.7%
Total	8	100.0%	8	100.0%	8	100.0%	8	100.0%	6	100.0%	38	100.0%
Clinical stage												
Stage 3B (IIIB)	0	0.0%	1	12.5%	1	12.5%	2	25.0%	2	33.3%	6	15.8%
Stage 3C (IIIC)	3	37.5%	4	50.0%	3	37.5%	4	50.0%	3	50.0%	17	44.7%
Stage 3D (IIID)	5	62.5%	3	37.5%	4	50.0%	2	25.0%	1	16.7%	15	39.5%
Total	8	100.0%	8	100.0%	8	100.0%	8	100.0%	6	100.0%	38	100.0%
Presentation of disease												
De novo	2	25.0%	4	50.0%	4	50.0%	4	50.0%	3	50.0%	17	44.7%
Recurrence	6	75.0%	4	50.0%	4	50.0%	4	50.0%	3	50.0%	21	55.3%
Total	8	100.0%	8	100.0%	8	100.0%	8	100.0%	6	100.0%	38	100.0%
Pretreatment status												
Prior surgery	6	75.0%	3	37.5%	4	50.0%	4	50.0%	3	50.0%	20	52.6%
Prior systemic therapy	0	0.0%	0	0.0%	0	0.0%	0	0.0%	0	0.0%	0	0.0%
Prior surgery and systemic therapy	0	0.0%	1	12.5%	0	0.0%	0	0.0%	0	0.0%	1	2.6%
N/A (For de novo presentations)	2	25.0%	4	50.0%	4	50.0%	4	50.0%	3	50.0%	17	44.7%
Total	8	100.0%	8	100.0%	8	100.0%	8	100.0%	6	100.0%	38	100.0%

Baseline clinical and demographic characteristics of patients enrolled in the Emory neoadjuvant trial, stratified by treatment arm. Variables include age, sex, race, ethnicity, ECOG performance status, mutation status, clinical stage, disease presentation, and prior treatment history. Data are presented as *n* (%) unless otherwise indicated.

Pepinemab-containing regimens were well tolerated, without additional toxicities beyond those expected from ICI therapy alone. Importantly, no patient experienced toxicity requiring delay or discontinuation of therapy or planned surgery. Among the 32 patients receiving neoadjuvant therapy, 12 (37.5%) completed 13 cycles of adjuvant nivolumab, 11 (34.4%) discontinued adjuvant therapy early because of recurrence, 7 (21.9%) discontinued because of toxicity, and 2 (6.3%) were lost to follow-up after surgery. Grade 3 adverse events (AEs) occurred most frequently in patients receiving ipilimumab-containing regimens. Specifically, 5 of 8 patients receiving pepinemab/nivolumab/ipilimumab experienced grade 3 AEs, including dermatitis, adrenal insufficiency (AI), colitis, enteritis, and nephritis, whereas 3 of 8 patients receiving pepinemab/ipilimumab experienced grade 3 AEs, including AI, thrombocytopenia, and transaminitis. In contrast, only 1 of 16 patients treated without ipilimumab experienced a grade 3 AE. No grade 4 or 5 AEs were observed with either neoadjuvant or adjuvant therapy (Supplementary Table 4).

Pathologic response was analyzed as a secondary endpoint and demonstrated numerically higher mPR rates in patients receiving pepinemab in combination with nivolumab and ipilimumab; however, these findings should be interpreted cautiously given the small cohort sizes and non-randomized study design (Supplementary Fig. 1a–c).

This pilot, biomarker-driven study incorporated multiple translational platforms with distinct tissue and biospecimen requirements to address its primary endpoint of evaluating phenotypic changes in the TME. To appreciate the evaluable sample numbers and how they varied across analyses, a CONSORT-style overview of patient enrollment and assay-specific sample availability is shown in Supplementary Fig. 2. Variability in tissue quality and specimen availability resulted in differing numbers of evaluable samples across multiplex immunohistochemistry (mIHC), bulk RNA profiling, GeoMx spatial profiling, immune repertoire sequencing, and serum cytokine analyses. GeoMx analyses were performed at the level of individual lymphoid aggregates rather than by patient, resulting in aggregate-level sample counts that differed from patient-level analyses.

### Pepinemab-containing regimens are associated with transcriptional remodeling of antigen presentation and immune recruitment pathways in melanoma tumors

**Fig. 2:**
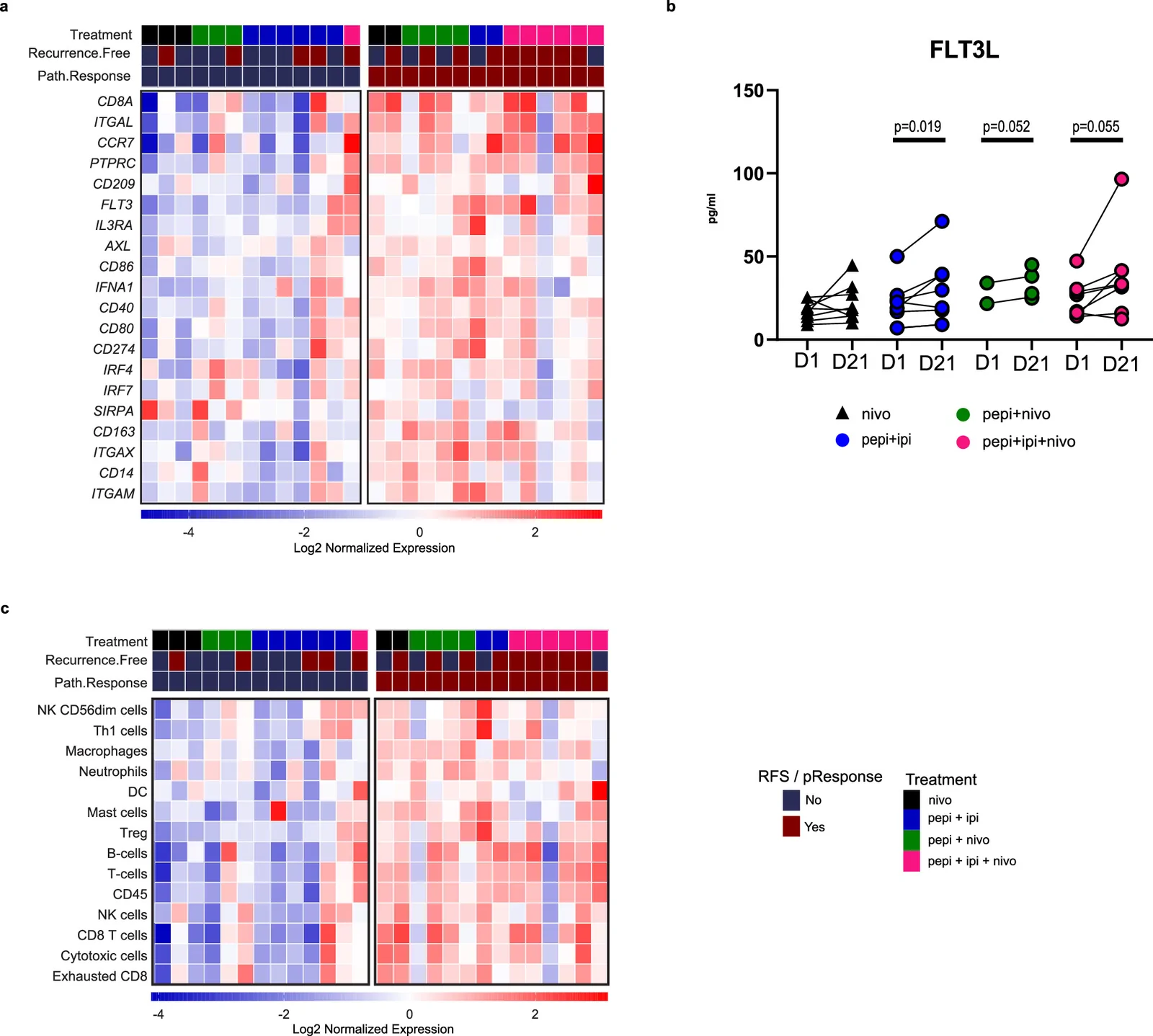
Transcriptional signatures of antigen presentation and immune recruitment in melanoma tumors treated with pepinemab-containing regimens compared with nivolumab alone. **a** Heatmap showing expression of dendritic cell–associated markers, cytokines, and transcription factors identified by ROSALIND cell type analysis. Values represent mean log2-normalized expression for each patient. Patients are annotated by treatment group, recurrence-free survival (RFS) status, and pathologic response. RFS status was defined as recurrence-free for >1 year after surgery. **b** Plasma FLT3L concentrations at baseline (day 1, D1) and after treatment (day 21, D21) for patients receiving nivolumab (*n* = 8), pepinemab plus ipilimumab (*n* = 8), pepinemab plus nivolumab (*n* = 5), or pepinemab plus ipilimumab and nivolumab (*n* = 8). *P* values were calculated using two-sided paired *t*-tests. **c** Heatmap showing inferred immune cell-type abundance based on ROSALIND cell type analysis. Values represent mean log2-normalized expression scores for each cell type and patient. Patients are annotated as in (**a**). Source data are provided as a Source Data file. *N* values can be found in Supplementary Table 3. pepi pepinemab, nivo nivolumab, ipi ipilimumab, RFS recurrence-free survival, pResponse pathologic response includes, PR partial response, mPR major pathologic response, CR complete response, DC dendritic cell, NK natural killer.

To assess treatment-associated immune changes, transcriptional profiling of tumor samples was performed. These analyses identified enrichment of transcripts associated with activated dendritic cells (DCs) and immune recruitment pathways in tumors from patients receiving pepinemab-containing regimens (Fig. 2a). Consistent with these findings, circulating FLT3L protein, a key regulator of DC development and cDC1 differentiation, was elevated in patients treated with pepinemab-containing regimens (Fig. 2b). Across treatment groups, tumors from patients with pathologic response demonstrated enrichment of lymphoid-associated transcripts, including genes linked to B cells, T cells, and DCs (Fig. 2c). Consistent with these findings, tumors from patients with pathologic response also demonstrated enrichment of activated DC-associated transcriptional programs.

Within tumors, the combination of pepinemab and nivolumab was associated with enrichment of transcripts related to NF-κB, EMT, and MAPK signaling pathways compared with nivolumab alone (Supplementary Fig. 3a, b). Among patients with pathologic response, tumors from patients receiving pepinemab/nivolumab/ipilimumab demonstrated a distinct pattern of NF-κB pathway gene expression relative to other responders, including transcripts associated with non-canonical NF-κB signaling involving the NFKB2/RelB complex (Supplementary Fig. 3c). Differential expression analyses also identified changes in EMT- and MAPK-related genes, including upregulation of FLT3, CXCR4, CCL21, and CCR7, which are associated with immune cell recruitment, alongside downregulation of MET, HGF, GREM1, and CCL2, which influence antigen-presenting cell migration and polarization (Supplementary Fig. 3d, e).

Together, these findings suggest that pepinemab-containing regimens are associated with transcriptional programs linked to enhanced antigen presentation and immune cell recruitment within the TME. These transcriptional changes prompted further evaluation of treatment-associated alterations in the spatial organization of immune cells within the TME.

### Pepinemab-containing regimens are associated with spatial immune remodeling and altered lymphoid cell localization within tumors

Enhanced immune infiltration was validated in patient lymph node resection specimens using mIHC. For these studies, the tumor bed area following neoadjuvant therapy was identified by regions harboring SOX10-expressing melanoma cells and/or containing evidence of tumor regression, including fibrosis and altered lymph node architecture. To provide contextual comparison for dual nivolumab/ipilimumab therapy, samples from an external neoadjuvant melanoma cohort were analyzed using harmonized mIHC methods.

**Fig. 3:**
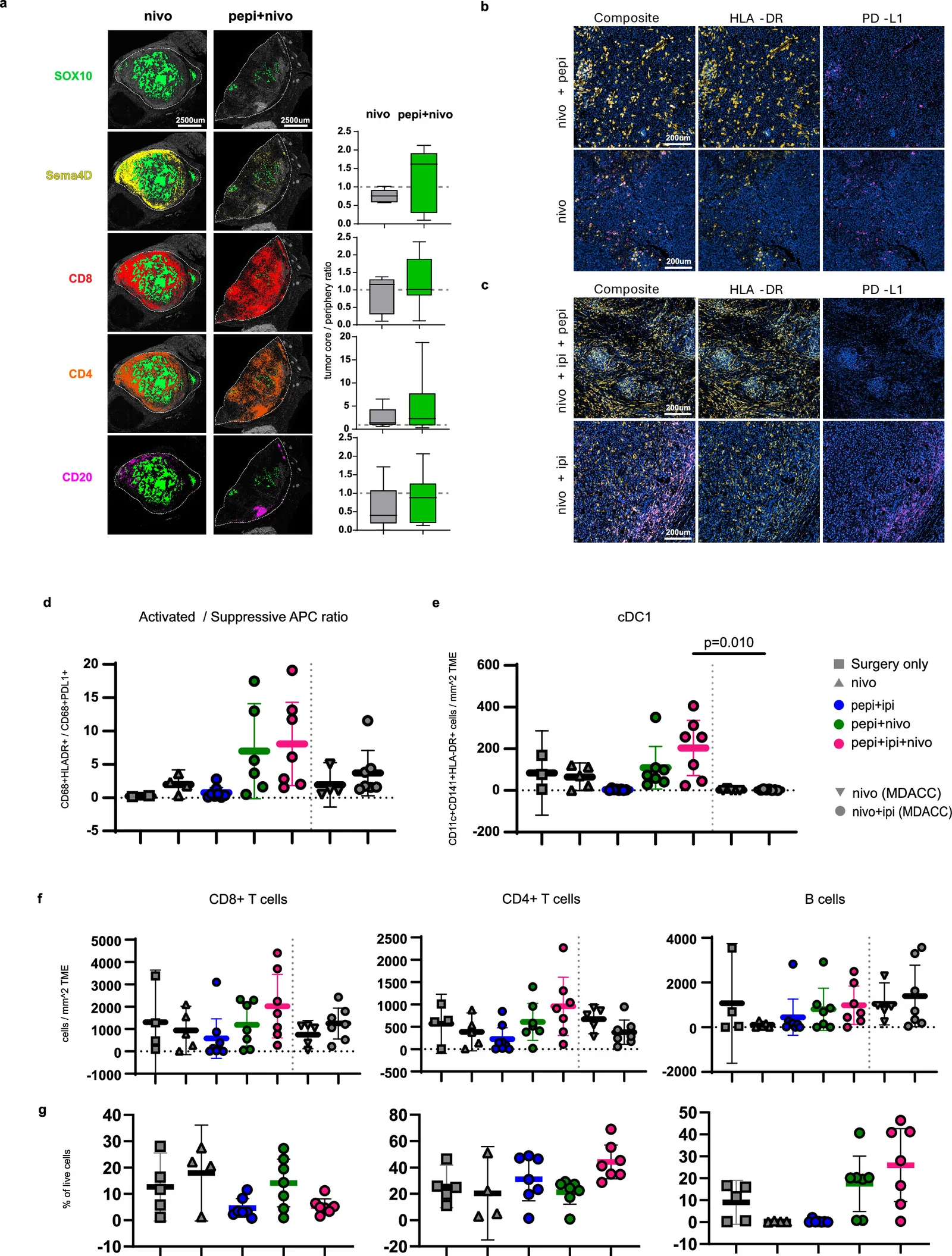
Pepinemab treatment is associated with altered immune cell distribution and antigen-presenting cell activation in melanoma. **a** Representative pseudo-colored inverted multiplex immunohistochemistry (mIHC) images and quantification of tumor core-to-periphery density ratios for SOX10⁺ tumor cells, SEMA4D⁺ cells, CD8⁺ T cells, CD4⁺ T cells, and CD20⁺ B cells in tumors from patients treated with nivolumab (nivo; *n* = 5) or pepinemab plus nivolumab (pepi+nivo; *n* = 7). The tumor periphery was defined as the region extending 1000 μm inward from the tumor bed edge, with the remaining area designated as the tumor core. Representative pseudo-colored inverted mIHC images of HLA-DR and PD-L1 expression in on-treatment tumor resections from patients treated with **b** nivo or pepi+nivo and **c** nivo+ipi or pepi+nivo+ipi. HLA-DR⁺ cells are shown in yellow and PD-L1⁺ cells in magenta. Representative images based on mean data from (**d**). **d** Quantification of the activated-to-suppressive antigen-presenting cell (APC) ratio, defined as CD68⁺HLA-DR⁺ cells divided by CD68⁺PD-L1⁺ cells. **e** Quantification of conventional type 1 dendritic cells (cDC1s; CD11c⁺CD141⁺HLA-DR⁺). **f** Quantification of CD8⁺ T cells, CD4⁺FoxP3⁻ T cells, and CD20⁺ B cells by mIHC. **g** Quantification of CD8⁺ T cells, CD4⁺ T cells, and CD19⁺ B cells among live cells by flow cytometry. Sample sizes varied across analyses because of tissue availability and assay quality control; exact sample sizes for each panel are provided in Supplementary Table 3. For box-and-whisker plots, center lines indicate the median, boxes indicate the interquartile range (25th–75th percentiles), and whiskers indicate the minimum and maximum values. For scatter plots, individual points represent individual patients, center lines indicate the mean, and error bars indicate the 95% confidence interval. Statistical analyses were performed using ordinary one-way ANOVA with correction for multiple comparisons. Exact *P* values are shown where significant. Data from the external MD Anderson Cancer Center (MDACC) cohort are indicated by a vertical dotted line separating MDACC and Emory samples. Source data are provided as a Source Data file.

In this small cohort, tumors from patients treated with single agent nivolumab appeared to demonstrate higher SEMA4D expression and features of immune exclusion at the tumor margins. In contrast, tumors from patients receiving pepinemab in combination with nivolumab demonstrated greater CD4⁺ and CD8⁺ T cell infiltration into the tumor bed (Fig. 3a). Foci of CD20⁺ B cells were also observed within the tumor bed in patients receiving pepinemab-containing regimens, whereas B cells were largely confined to the tumor margins in tumors from patients treated with nivolumab alone (Fig. 3a and Supplementary Fig. 4a, b).

To further characterize treatment-associated changes in the TME, we examined the composition and activation state of myeloid populations. Consistent with the known effects of SEMA4D blockade on myeloid biology, analyses focused on markers of antigen presentation and immune suppression. Tumors from patients receiving pepinemab in combination with nivolumab demonstrated a higher proportion of HLA-DR⁺ antigen-presenting macrophages relative to PD-L1⁺ suppressive macrophages compared with tumors from patients treated with nivolumab alone, resulting in a numerically increased HLA-DR:PD-L1 ratio (Fig. 3b, d). A similar pattern was observed in tumors from patients receiving pepinemab/nivolumab/ipilimumab relative to the external comparator cohort treated with nivolumab plus ipilimumab (Fig. 3c, d). These findings are consistent with a shift toward a more antigen-presenting myeloid phenotype across treatment backbones.

We next evaluated dendritic cell subsets within the TME. Tumors from patients receiving the triple therapy demonstrated numerically higher frequencies of conventional type 1 dendritic cells (cDC1), with significantly higher levels relative to the external comparator cohorts (Fig. 3e). Notably, the nivolumab plus ipilimumab comparator group was derived from an external cohort, and these observations should therefore be interpreted cautiously.

Analyses of lymphocyte populations demonstrated variability across treatment groups, with no statistically significant differences in the overall abundance of CD4⁺ T cells, CD8⁺ T cells, or B cells in this cohort (Fig. 3f, g and Supplementary Fig 5a, b). However, as shown in Fig. 3a, differences in the spatial distribution of lymphoid cells within the TME were observed across treatment groups. Together, these findings suggest that pepinemab-containing regimens are associated with coordinated remodeling of both myeloid and lymphoid compartments within the TME.

### Pepinemab plus nivolumab-containing regimens are associated with increased frequency and maturation of lymphoid aggregates

To further examine these structural changes and based on prior studies linking TLS with clinical outcomes in melanoma treated with immune checkpoint blockade, we evaluated whether lymphoid aggregates (LAs) in this cohort exhibited features of TLS-like organization. Given the exploratory, biomarker-driven design and limited cohort size, analyses relating these features to clinical outcomes were not powered for definitive conclusions.

**Fig. 4:**
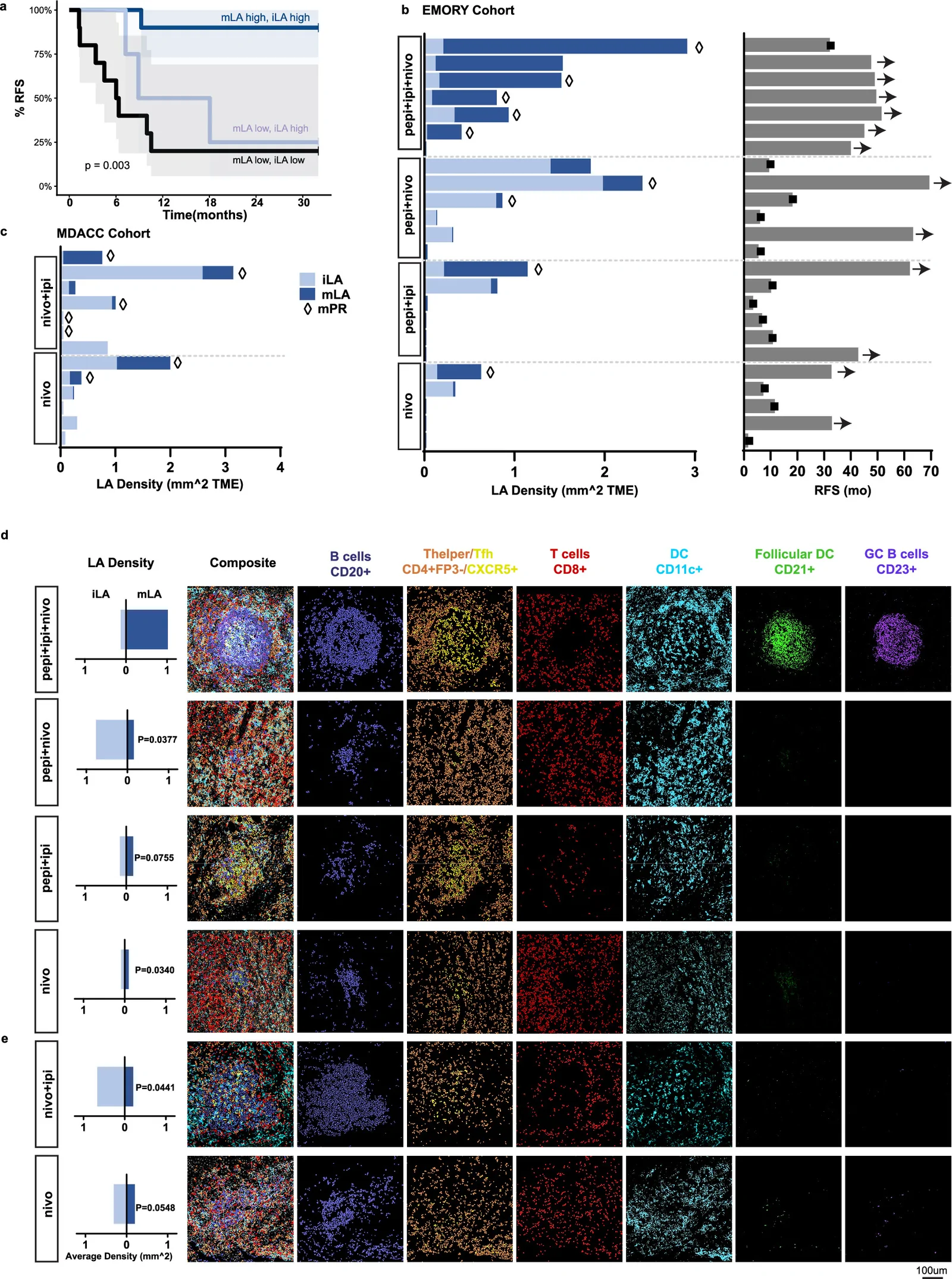
Lymphoid aggregate maturity is associated with recurrence-free survival in melanoma patients. **a** Kaplan–Meier analysis of recurrence-free survival (RFS) stratified by lymphoid aggregate (LA) maturity and abundance. Patients were grouped according to mature LA (mLA) and immature LA (iLA) density within the tumor microenvironment using maximally selected rank statistics (survminer cutpoint analysis). Shading represents the 95% confidence interval (CI). **b** Average density of immature lymphoid aggregates (iLA; CD20⁺CD21⁻CD23⁻) and mature lymphoid aggregates (mLA; CD20⁺CD23⁺) within the tumor bed for individual patients in the Emory cohort. Diamonds indicate major pathologic response (mPR) or pathologic complete response (pCR). Corresponding swimmer plots show RFS through 70 months of follow-up. Squares indicate recurrence or death, and arrows indicate patients without an event at the time of data cutoff. **c** Average density of iLA and mLA within the tumor bed for individual patients in the MD Anderson Cancer Center (MDACC) comparator cohort. Diamonds indicate mPR or pCR. **d** Representative pseudo-colored inverted multiplex immunohistochemistry (mIHC) images of lymphoid aggregates from the Emory cohort. Images were selected from tumors with iLA and mLA densities approximating the cohort mean shown in (**b**). Bar graphs show cohort-average iLA (light blue) and mLA (dark blue) density for each treatment group. Cell populations shown include CD20⁺ B cells, CD4⁺FoxP3⁻CXCR5⁺ T helper/T follicular helper cells, CD8⁺ T cells, CD11c⁺ dendritic cells, CD21⁺ follicular dendritic cells, and CD23⁺ germinal center B cells. **e** Representative pseudo-colored inverted mIHC images of lymphoid aggregates from the MDACC comparator cohort. Images were selected from tumors with iLA and mLA densities approximating the cohort mean shown in (**c**). Bar graphs show cohort-average iLA (light blue) and mLA (dark blue) density for each treatment group. Sample sizes for (**a**): mLA high/iLA high (*n* = 10), mLA low/iLA high (*n* = 4), and mLA low/iLA low (*n* = 10). Groups were defined using maximally selected rank statistics (survminer cutpoint analysis). Kaplan–Meier *P* values were calculated using a two-sided log-rank test. Exact sample sizes for each panel are provided in Supplementary Table 3. Asterisk denotes *P* < 0.05 relative to the pepi+ipi+nivo cohort; one-way ANOVA with Tukey’s multiple-comparison test (d,e). Scale bar, 100 μm (**d**, **e**). Differences in mTLS density among treatment groups were assessed by one-way ANOVA (*P* = 0.0154), followed by Tukey’s multiple-comparison test, with each treatment group compared with pepi+ipi+nivo; adjusted *P* values are shown in the graphs (**d**, **e**).

mIHC was used to further characterize the structural organization and maturity of intratumoral LAs using criteria commonly applied to TLS. Lymphoid aggregates were graded according to spatial proximity to tumor regions using a four-tier system (G1–G4), as shown in Supplementary Fig. 4c. mLAs were defined by the presence of spatially organized B and T cell zones, CD21⁺ follicular dendritic cells, and CD23⁺ germinal center B cells. LAs with these features were identified across treatment groups, with variability in their frequency and degree of organization (Fig. 4a–c).

Consistent with prior reports, high density of mLAs was associated with longer recurrence-free survival (Fig. 4a) and mLAs were observed in some patients with mPR (Fig. 4b). Median follow up was 59.8 months (range 2–89.3). Patients without recurrence were censored at the date of last documented follow-up or death. Differences in LA organization were also observed across treatment groups. Tumors from patients receiving doublet regimens (pepinemab plus nivolumab or nivolumab plus ipilimumab) demonstrated a higher frequency of immature lymphoid aggregates (iLAs), whereas features of mLAs were most evident in tumors from patients receiving pepinemab/nivolumab/ipilimumab triple therapy (Fig. 4b–e).

Together, these findings suggest that the addition of pepinemab to nivolumab-based regimens is associated with increased iLA formation and, in the context of nivolumab plus ipilimumab, features of LA maturation. These findings prompted further molecular characterization of lymphoid aggregates using spatial transcriptomic and immune repertoire analyses.

### Spatial transcriptomic profiling identifies adaptive immune programs and increased clonality within lymphoid aggregates

**Fig. 5:**
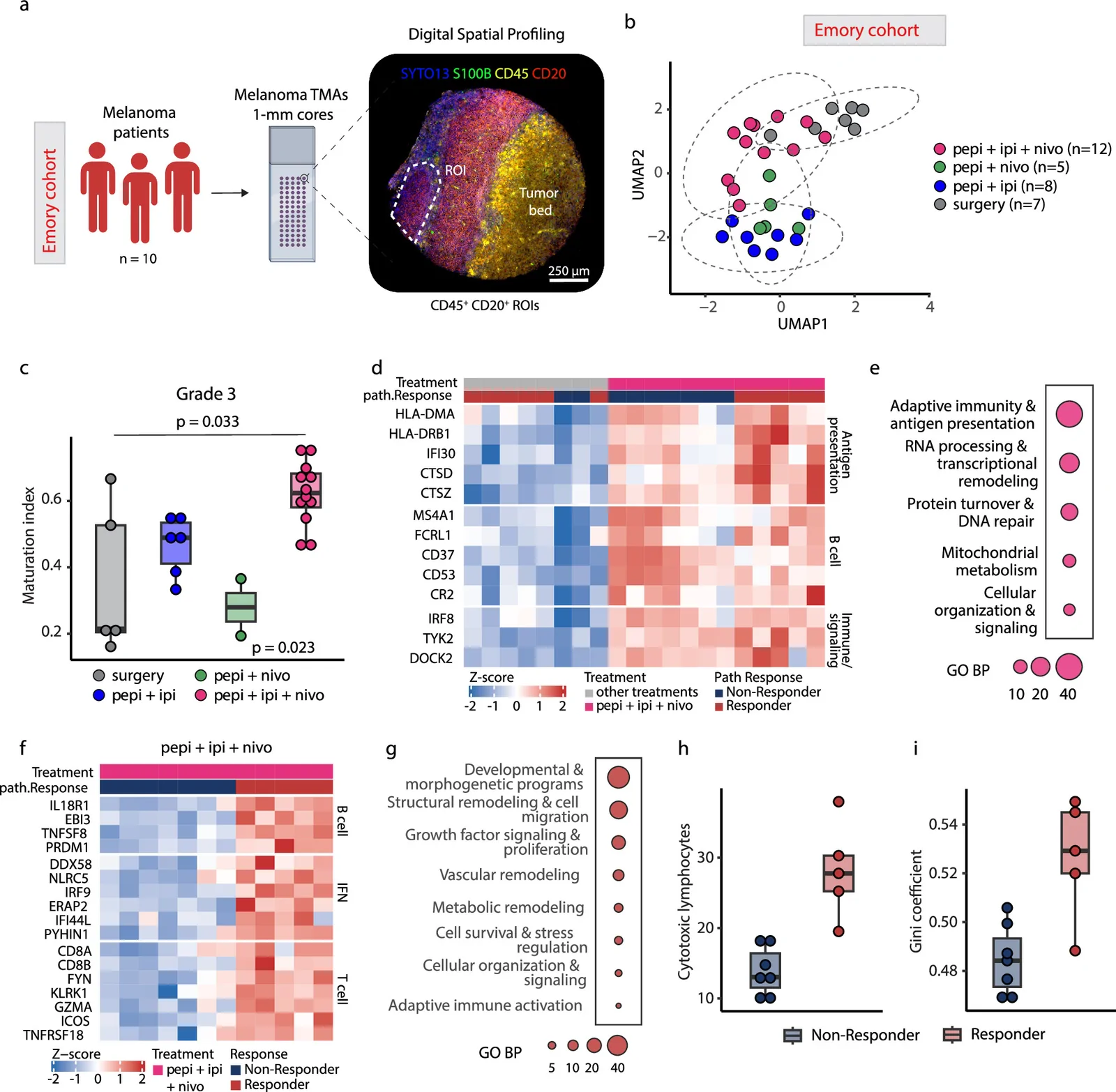
Clonal expansion of cytotoxic T cells within lymphoid aggregates in melanoma patients treated with pepinemab-containing regimens. **a** Overview of CD45⁺CD20⁺ lymphoid aggregate identification and region selection for GeoMx digital spatial profiling in melanoma patients treated with immune checkpoint inhibitors or surgery alone (Emory cohort). **b** Uniform Manifold Approximation and Projection (UMAP) of immune aggregates, where each point represents a single ROI; treatment-specific ROI counts are indicated in the legend. **c** Rank-based maturation index in grade 3 (G3) immune aggregates across treatments (surgery, 2 patients/5 ROIs; pepi + ipi, 2 patients/6 ROIs; pepi + nivo, 1 patient/2 ROIs; pepi + ipi + nivo, 3 patients/12 ROIs). Statistical comparisons were performed using a linear mixed-effects model with patient as a random effect to account for repeated measures, two-sided. **d** Heatmap of selected differentially expressed genes in G3 immune aggregates, grouped by B cell, antigen presentation, and immune signaling programs, comparing pepi + ipi + nivo (3 patients/12 ROIs) with all other ICI treatments (3 patients/8 ROIs), using a linear mixed-effects model with patient as a random effect and response as a covariate, two-sided (*p* < 0.05). Each value represents a single immune aggregate. **e** Summary of enriched Gene Ontology (GO) biological process categories in G3 immune aggregates evaluated using GSEA comparing pepi + ipi + nivo (3 patients/12 ROIs) with all other ICI treatments (3 patients/8 ROIs). Dot size indicates the number of significantly enriched pathways per category (FDR < 0.05). **f** Heatmap of differentially expressed immune activation genes in G3 immune aggregates from pepi + ipi + nivo-treated patients, comparing pathologic responders (2 patients/5 ROIs) versus non-responders (1 patient/7 ROIs), using a linear model, two-sided, with Benjamini-Hochberg multiple comparisons adjustment (FDR < 0.10). Genes are grouped by T-cell, IFN, and B cell programs. Each value represents a single immune aggregate. **g** Summary of GO biological process category enrichment (GSEA) in G3 immune aggregates from pepi + ipi + nivo responders (2 patients/5 ROIs) versus non-responders (1 patient/7 ROIs). Bubble size denotes the number of enriched pathways per category (FDR < 0.05). **h** Cytotoxic lymphocyte MCP scores in G3 immune aggregates from pepi + ipi + nivo responders (2 patients/5 ROIs) versus non-responders (1 patient/7 ROIs) presented descriptively. **i** T-cell receptor clonality in G3 immune aggregates from pepi + ipi + nivo responders (2 patients/5 ROIs) versus non-responders (1 patient/7 ROIs), quantified by the Gini coefficient and presented descriptively. For box-and-whisker plots, center lines indicate the median, boxes indicate the interquartile range (IQR), and whiskers extend 1.5 × IQR. pepi pepinemab, nivo nivolumab, ipi ipilimumab, pCR Responders: pathologic complete response, mPR major pathologic response, and pPR partial pathologic response. pNR Non-responders: pathologic non-response. **a** created in BioRender; Melendez, B. (2025) https://BioRender.com/nzw77wz.

Because lymphoid aggregates foster antigen presenting cell and T cell interactions that support adaptive immunity, we next examined transcriptional programs and immune repertoires within these structures. Surgical resection samples underwent Bruker Spatial GeoMx spatial profiling to characterize the immune architecture of lymphoid aggregates and to delineate treatment-associated differences linked to pepinemab-containing regimens. A total of 32 regions of interest (ROIs) containing lymphoid aggregates, including mature aggregates with germinal center features, were selected from representative melanoma specimens across treatment groups and the external comparator cohort (Fig. 5a and Supplementary Fig. 6a). ROIs clustered according to treatment group and cohort, indicating distinct transcriptional programs associated with different therapeutic regimens (Fig. 5b and Supplementary Fig. 6b).

To further characterize mLAs identified by mIHC, a rank-based maturation index integrating germinal center B cell, follicular dendritic cell, and T follicular helper cell transcriptional signatures was applied (Supplementary Fig. 6c). G3 lymphoid aggregates demonstrated higher maturation index scores in tumors from patients receiving triple therapy compared with surgery alone or pepinemab/nivolumab (Supplementary Fig. 6c). In contrast, the external comparator cohort demonstrated a broader distribution of maturation scores without evidence of treatment-specific enrichment (Supplementary Fig. 6d).

Consistent with these observations, G3 lymphoid aggregates from the triple therapy group demonstrated increased expression of genes associated with antigen presentation and follicular organization, including *HLA-DMA, HLA-DRB1, CTSZ, and CR2*, (Fig. 5d). Corresponding changes in CR2 (CD21) were also observed by mIHC (Fig. 4d, e). Pathway-level analyses identified enrichment of programs related to B cell receptor signaling pathways and antigen presentation with additional enrichment of RNA processing and transcriptional remodeling (Fig. 5e and Supplementary Fig. 6e–g).

Within the triple therapy group, G3 lymphoid aggregates were enriched in patients with pathologic response relative to non-responders, including transcriptional programs associated with cytotoxic T cells, interferon responses, antigen processing, and B cell differentiation (Fig. 5f). Gene set enrichment analyses showed upregulation of pathways related to structural and extracellular matrix remodeling, vascular remodeling, growth factor signaling, developmental processes, and adaptive immune activation (Fig. 5g and Supplementary Fig. 6f). These transcriptional features were observed alongside a pattern of higher cytotoxic lymphocyte scores and TCR clonality (Fig. 5h, i). Considering the limited number of patients, these findings are exploratory.

These findings were integrated with bulk BCR and TCR repertoire analyses by end-to-end immunosequencing. Tumors from patients with pathologic response tended to contain fewer unique BCR and TCR clonotypes and reduced repertoire diversity, as measured by Chao1 and D50 indices (Supplementary Fig. 7a–e). Notably, patients with pathologic response demonstrated a predominantly oligoclonal BCR repertoire characterized by large or hyperexpanded clones comprising greater than 25% of total clonal abundance (Supplementary Fig. 7b, c). No significant differences in TCR or BCR gene usage were identified across treatment groups (Supplementary Fig. 8a–d).

### Pepinemab-containing regimens are associated with peripheral cytokine, chemokine, and B cell changes

Systemic immune changes associated with treatment were evaluated using plasma cytokine analyses and flow cytometric profiling of peripheral blood mononuclear cells (PBMCs). In plasma from patients treated with pepinemab-containing regimens, increased levels of soluble cytotoxic mediators, including perforin, granzyme A, TNF-α, and sFasL, were observed (Supplementary Fig. 9a). While CXCL9 increased following treatment across multiple regimens, CXCL10 and CXCL11 were preferentially elevated in patients receiving triple therapy (Supplementary Fig. 9b). Growth factors associated with myeloid and B cell maturation, including BAFF and FGF-2, were also increased in patients receiving the triple combination regimen. (Supplementary Fig. 9c). Increased BAFF levels observed in patients receiving triple therapy were consistent with the non-canonical NF-κB-associated transcriptional programs identified in tumor analyses above. Representative flow cytometry gating strategies used to define peripheral immune populations are shown in Supplementary Fig. 10a. Peripheral B cell populations associated with extrafollicular responses, including DN2 B cells and antibody-secreting cell precursors, were elevated in patients receiving pepinemab/ipilimumab and modestly increased in patients receiving pepinemab/nivolumab/ipilimumab (Supplementary Fig. 10a–c). No clear association between peripheral B cell responses and clinical outcomes was identified. Plasma IgG3 levels were also decreased following treatment with pepinemab/nivolumab/ipilimumab compared with pretreatment samples (Supplementary Fig. 10d).

## Discussion

This pilot biomarker-driven study revealed that pepinemab, a first-in-class SEMA4D-blocking antibody, had manageable toxicity profiles in combination with ICI and was associated with immune modulation in the neoadjuvant setting. Our data suggest that pepinemab and ICI combinations are associated with remodeling of the TME, including the presence of mLAs consistent with organized, APC-associated antitumor immunity. In patients with resectable stage III melanoma, a high density of mLAs was observed, which was associated with longer recurrence-free survival, following treatment with neoadjuvant pepinemab combined with nivolumab and ipilimumab without evidence of additional toxicity. Notably, all treated patients proceeded to curative-intent surgery without delay.

Biomarker analyses of patient tumors identified changes consistent with proposed mechanisms of pepinemab activity described in prior preclinical studies, including enhanced antigen presentation and antitumor immune responses^17,19^. Treatment-associated remodeling of the myeloid compartment was observed, including altered expression of genes linked to macrophage and dendritic cell maturation, migration, antigen processing, and antigen presentation. We observed features consistent with increased antigen-presenting macrophage states and reduced suppressive macrophage-associated features in tumors from patients receiving pepinemab-containing regimens. In parallel, frequencies of cDC1 cells, which have been implicated in lymphoid aggregate development, appeared elevated in tumors from patients treated with pepinemab-containing regimens. Consistent with this observation, tumor expression of FLT3-associated genes and circulating FLT3L protein, both linked to cDC1 development and function^24,25^ were elevated in patients receiving pepinemab-containing therapy. Tumors from pepinemab-treated patients also demonstrated alterations in EMT- and MAPK-associated transcriptional programs. These findings may reflect, in part, modulation of the MET–PlexinB1/2 signaling axis following SEMA4D inhibition.

A central observation of this study was the association between pepinemab-containing regimens and the organization of immune infiltrates into mLAs. In patients with stage III melanoma, receipt of the triplet therapy in particular, was associated with prominent intratumoral B cell foci, including structures resembling germinal centers and organized lymphoid aggregates. Although prior studies have demonstrated that immunotherapy can influence lymphoid aggregate abundance^26,27^, our findings suggest that pepinemab and ICI treatment may additionally influence aggregate maturity, defined by active germinal centers, compartmentalized B and T cell zones, and follicular dendritic cells. When combined with nivolumab, pepinemab was associated with enrichment of extracellular matrix-related pathways, which may contribute to the structural organization required for lymphoid aggregate formation. In contrast, addition of pepinemab to nivolumab and ipilimumab was associated with enrichment of cell cycle and non-canonical NF-κB signaling pathways. Together, these findings are consistent with enhanced immune organization and activation within lymphoid aggregates, including features associated with memory B cell and plasma cell development that may support durable immune responses. Consistent with this interpretation, increased frequency and density of mLAs were observed in patients treated with pepinemab-containing regimens and were associated with patients with longer recurrence-free survival.

The increased abundance and maturity of lymphoid aggregates, together with enrichment of genes associated with cell proliferation, antigen cross-presentation, and adaptive immune activation, are consistent with features associated with productive antitumor immune responses in patients receiving pepinemab and ICI-containing regimens. This interpretation is further supported by findings that patients with pathologic response demonstrated oligoclonal B cell repertoires and more clonal, less diverse TCR repertoires within lymphoid aggregates relative to patients treated with ICI alone. Although these analyses were limited by insufficient pretreatment tissue for paired BCR and TCR repertoire analyses, they provide insight into potential differences in adaptive immune responses across treatment groups.

Collectively, these findings suggest that the myeloid-remodeling effects of pepinemab may help overcome barriers imposed by suppressive myeloid populations and impaired antigen presentation within solid tumors. The observed APC-associated remodeling and lymphoid organization may therefore create conditions favorable for coordinated adaptive immune responses together with ICI therapy. These findings also raise the possibility that pepinemab-containing regimens could complement additional immunotherapeutic approaches, including cancer vaccines, agonist antibodies, emerging checkpoint inhibitors, or adoptive cellular therapies. Although this study focused on advanced melanoma, this neoadjuvant strategy is also under investigation in resectable HNSCC (NCT03690986). Pepinemab has additionally been administered safely in combination with ICI in metastatic NSCLC and HNSCC (NCT03268057, NCT04815720), with ongoing investigation in pancreatic and breast cancers (NCT05102721, NCT05378464). Together, these observations suggest that the mechanisms identified here may have broader relevance across multiple solid tumor settings.

This study represents an important initial step toward evaluating the safety, feasibility, and immune modulatory activity of pepinemab administered with ICI in the neoadjuvant melanoma setting. While we observed grade 3/4 treatment related adverse events, the small cohort size limits our ability to draw definitive conclusions on how these compare across other previous studies. A limitation of the study design is the absence of a concurrent nivolumab/ipilimumab-only control arm. To partially address this limitation, we incorporated samples from a similarly designed neoadjuvant melanoma study conducted at MD Anderson Cancer Center (NCT02519322)^28^ as an independent contextual comparator cohort for biomarker analyses. Although direct comparisons across independent clinical trials should be interpreted cautiously, harmonized analysis of tumor specimens from patients with comparable eligibility criteria provided additional context for interpretation of treatment-associated biomarker findings. We also note that this study was designed primarily as an integrated biomarker analysis and was not powered to assess definitive clinical efficacy outcomes. Accordingly, the clinical observations described here as secondary endpoints of the clinical trial should be considered exploratory. Given the biologic and pathologic findings observed in this study, further evaluation of pepinemab-containing regimens in larger randomized trials is warranted. Finally, although the observed changes in lymphoid aggregate organization are notable, these analyses were limited to post-treatment specimens. Future studies incorporating matched pre- and post-treatment biopsies will be important to determine whether pepinemab contributes to de novo lymphoid aggregate formation, as these structures have been associated with patient survival and immunotherapy response.

In summary, neoadjuvant pepinemab combined with ICI was associated with remodeling of the TME consistent with enhanced immune activation. Collectively, these findings suggest that SEMA4D blockade may complement ICI therapy through mechanisms involving myeloid remodeling, antigen presentation, and lymphoid aggregate maturation. These data support further evaluation of pepinemab-containing immunotherapy regimens in melanoma and other solid malignancies.

## Methods

### Patient cohorts

This study complies with all relevant ethical regulations. All patients provided informed written consent prior to participating in this study, and the study was approved by the Institutional Review Board (Emory University IRB00104273). All patients from the MDACC cohort provided written informed consent for participation in the clinical trial (NCT02519322)^28^. The study design and conduct comply with all relevant regulations regarding the use of human study participants and was conducted in accordance with the criteria set by the Declaration of Helsinki.

### Clinical trial design

This study was preregistered with Clinicaltrials.gov on December 7th, 2018 (https://clinicaltrials.gov/study/NCT03769155) Patients with resectable stage IIIB/C/D melanoma, who were immunotherapy naïve and with ECOG performance status of 0 or 1, were sequentially enrolled into one of four treatment cohorts (*n* = 8 each): pepinemab/nivolumab, pepinemab/ipilimumab, pepinemab/nivolumab/ipilimumab, or nivolumab alone (NCT03769155). The first patient was enrolled on December 13th, 2018, and last patient enrolled on December 15th, 2023. Given this inability to randomize across five arms, we chose sequential enrollment to reduce selection bias and minimize concentration of specific variables, e.g., BRAF tumor status, in one arm. For ease of enrollment, pre-treatment research biopsy was not mandated; archival tissue was available for all patients, and most of these were procured by fine needle aspiration to minimize patient impact. These samples were not suitable for spatial mIHC analysis of the tumor architecture. Pepinemab was provided by Vaccinex; nivolumab and ipilimumab were provided by Bristol Myers Squibb. Two doses were given on days 1 and 21 (15 mg/kg pepinemab; 360 mg nivolumab, 1 mg/kg ipilimumab); surgery occurred on between days 35–42. Patients received standard of care adjuvant nivolumab every 4 weeks to complete 1 year of treatment at the discretion of the treating physicians. A control cohort underwent surgery without neoadjuvant therapy (*n* = 6). The primary endpoint of the study was to evaluate the effect of pepinemab in combination with immune checkpoint inhibitors on modulation of the phenotypic properties within the TME. The secondary endpoint was major pathologic response (pMR), including complete and near complete responses (pCR) according to International Neoadjuvant Melanoma Consortium guidelines^29^. Secondary endpoints included safety, surgical delays, ORR, RFS, EFS, and OS. Radiographical response was not reported in the manuscript. Peripheral blood was drawn at three time points: (a) pre-treatment, (b) two weeks into treatment, and (c) at time of surgery. Surgically resected lymph nodes were collected for pathologic response and biomarker analysis.

### Sample processing and flow cytometry staining

Patient tissue resections were stored in hypothermosol for 24 h at 4 °C prior to manual dissociation with a razor, followed by digestion with the GentleMACS Tumor Dissociation Kit (Miltenyi Biotec cat no. 130-095-929). The resulting cell suspension was stained using antibodies listed in (Supplementary Table 1). Surface stains were incubated at 4 °C for 30 min in the dark. Cells were then fixed using Cytofix and SEMA4D saturation samples were stored at 4 °C overnight. Samples were analyzed on a FACSymphony flow cytometer the following day. Where appropriate, samples were then briefly washed with PermWash before freezing in 90%FBS/10% DMSO (freezing media) until batch staining for intracellular markers. Batch staining was performed on frozen TILs and patient PBMCs. PBMCs were thawed in a 37 °C water bath, centrifuged to remove freezing media, then plated to be surface stained. TILs were thawed in PermWash in a 37 °C incubator, then centrifuged to remove freezing media before plating with surface stained PBMCs. All samples were then stained for intracellular markers in PermWash at 4 °C in the dark for 1 h. TILs and PBMCs were run on a FACSymphony flow cytometer and cell populations were identified and analyzed using FlowJo v10 (BD Biosciences).

### Nanostring nCounter assay

RNA quality control using a Bioanalzyer (Agilent). The NanoString nCounter assay was performed per NanoString instructions, using the Tumor Signaling 360 panel. Specifically, the equivalent of 50 ng of RNA (adjusted for DV200) was hybridized to the Tumor Signaling 360 Reporter CodeSet and Capture ProbeSet. A panel standard was performed with the samples. The hybridized RNA was loaded onto the NanoString FLEX Prep Station in order to load the RNA onto the sample cartridge. The sample cartridge was read by the NanoString FLEX Digital Analyer. nCounter results were analyzed using nSolver (NanoString).

### Multiplex immunohistochemistry staining

FFPE tissue was cut at 5 µm and placed on charged slides. Slides were then baked overnight at 60 °C. Slides were deparaffinized in xylenes and rehydrated in an alcohol gradient. Slides were run through a chromogenic sequential stain and strip multiplexed IHC process. Antigen retrieval was performed using HIER citrate pH6.1 (S169984-2, Agilent/Dako). IHC staining was performed using an automated autostainer (Universal staining system, Dako). Slides were blocked for endogenous peroxidase (PX968, Biocare medical) for 20 min, followed by serum free protein block (ab64226, Abcam) for 30 min. Sections were incubated with primary antibodies (see supplemental antibody chart (Supplementary Table 2)) for 60 min, followed by amplification with either mouse or rabbit EnVision+ HRP secondary (K4001 or K4003, Agilent/Dako) and visualized using chromogenic ImmPACT AEC substrate kit (SK-4205, Vector Laboratories), then counterstained using hematoxylin (S330130-2, Agilent/Dako). Slides were scanned at 40× using Motic slide scanner. Slides were then stripped using gradient alcohols and HIER citrate pH6.1 (S169984-2, Agilent/Dako). Slides were sequentially stained for 10–14 antibodies per panel. Whole slide digital images acquired for each stain at 40x using a Motic digital scanner.

### Virtual multiplexed immunohistochemistry image analysis

Sequentially stained whole-slide image (WSI) files were digitally aligned using Tissuealign (Visiopharm®). ROIs were manually annotated based on pathologist-confirmed tumor bed areas, defined as intra-tumoral regions and tumor regression beds comprising viable tumor, tumor-associated stroma, immune infiltrate, necrosis, and/or fibrotic tissue. Adjacent normal lymphoid tissue was excluded. Image analysis algorithms were implemented in Visiopharm Oncotopix® to segment and phenotype single cells within virtually aligned multiplexed WSIs. For each ROI, cell classifications, density metrics, and spatial features—including clustering and neighborhood relationships—were extracted and exported for statistical analysis in GraphPad Prism.

B-cell aggregates were identified using heatmaps and spatial clustering algorithms, defined as clusters containing ≥20 B cells. For each aggregate, the ROI was expanded by 125 µm to delineate the surrounding aggregate neighborhood.

iLA were defined as clusters of ≥20 B cells in close association with mixed CD4⁺ and CD8⁺ T cells and CD11c⁺ dendritic cells, without evidence of structural compartmentalization.

mLAs met the criteria for iLA and also exhibited organized architecture, including distinct B cell and T cell zones, including CXCR5^+^CD4^+^ T follicular helper cells and the presence of activated germinal centers. Germinal centers were classified by the presence of ≥20 CD21⁺ follicular dendritic cells and/or CD23⁺ germinal center B cells.

Spatial grading of lymphoid aggregates (LAs) Lymphoid aggregates were graded based on spatial proximity to tumor regions using a four-tier scale (G1–G4): G1: Located within viable tumor islands, G2: Within the tumor-associated bed, G3: At the edge or immediately adjacent to the tumor-associated bed, G4: Distal to the tumor-associated bed. Grades G1–G3 exhibited atypical lymph node architecture, whereas G4 most closely resembled normal lymph node structure and was therefore excluded in both mIHC and GeoMX analyses.

### Human samples

MDACC cohort: FFPE melanoma tissue specimens were obtained from twelve consented patients with high-risk stage III–IV melanoma treated with neoadjuvant nivolumab or nivolumab plus ipilimumab [NCT02519322] at The University of Texas MD Anderson Cancer Center. Tumor samples were collected and analyzed under Institutional Review Board (IRB)-approved protocols (2015–0041, 2012–0846). These studies were conducted in accordance with the Declaration of Helsinki and approved by the UT MD Anderson Cancer Center IRB.

### Tissue microarray

For tissue microarray (TMA) construction, hematoxylin and eosin (H&E)-stained sections from formalin-fixed, paraffin-embedded tissue blocks were reviewed by a board-certified pathologist (A.J.L.) to identify areas of viable tumor and lymphoid immune aggregates. CD20 immunohistochemistry was used to guide identification of immune aggregate-rich regions.

Using a semi-automated tissue microarrayer (ATA-27; Beecher Instruments), 1-mm diameter cores were extracted from donor blocks and assembled into a standard recipient paraffin block (4.5 × 2 × 1 cm³). Where feasible, multiple cores were obtained per case to represent tumor or tumor bed regions and spatially distinct immune aggregate–containing areas.

### Digital spatial profiling

#### Sample processing

FFPE tissue sections were deparaffinized and hybridized with the human Whole Transcriptome Atlas (WTA) probe set (Bruker Spatial Biology), together with the TCR Profiling add-on, comprising 146 probes targeting variable and joining regions of α, β, γ, and δ TCR chains. Morphology markers included Alexa488-SYTO 13 (nuclei; 1:10,000), Alexa532-S100B (melanoma cells; 1:50), Alexa594-CD45 (immune cells; 1:100), and Alexa647-CD20 (B cells; 1:100). ROIs were selected around CD45⁺CD20⁺ immune aggregates with germinal center-like features (Fig. 5a). Oligonucleotide barcodes were cleaved using the GeoMx Digital Spatial Profiler (Bruker Spatial Biology) and sequenced on a NovaSeq 6000 (Illumina) at the MD Anderson Advanced Technology Genomics Core. The Emory and MDACC cohorts were processed and sequenced as independent experimental batches using identical reagents, protocols, and downstream analytical workflows.

#### Quality control and normalization

ROIs were retained if they met the following criteria: ≥80% trimmed, stitched, and aligned reads; ≥50% sequencing saturation; and ≥1000 total reads. The limit of quantification was defined as two geometric standard deviations above the geometric mean of negative control probes. ROIs with <10% gene detection and genes detected in <5% of ROIs were excluded. To preserve TCR diversity, TCR probes detected in ≥2.5% of ROIs were retained.WTA counts were Q3-normalized to the 75th percentile of gene expression within each ROI using the GeomxTools R package. A pseudocount of 1 was added to normalized values to enable log-based analyses.

#### Dimensionality reduction

Uniform Manifold Approximation and Projection (UMAP) was performed on log2 Q3-normalized counts using the umap R package defaults. Immune aggregates were colored by treatment arms.

#### Maturation index

A rank-based maturation index was defined using transcriptional programs associated with germinal center organization and function. These included a germinal center B cell program (*BCL6, AICDA, MEF2B, RGS13*)^1,2^, a follicular dendritic cell (FDC) network program reflecting stromal organization and antigen retention (*CXCL13, MFGE8, PDPN, CLU, FDCSP*)^3–5^, and a T follicular helper cell support program (*PDCD1, ICOS, CD40LG, SH2D1A, MAF*)^6^. For each ROI, module scores were converted to percentile ranks within the cohort, and the maturation index was defined as the mean of these ranked components, providing a relative measure of immune aggregate maturation.

#### Differential gene expression and pathway analysis

Differential gene expression analyses were restricted to G3 immune aggregates from the Emory cohort, excluding the surgery arm, as pathologic response status was not available for these patients. For the treatment comparison, pepi + ipi + nivo aggregates were compared against all other aggregates using a linear mixed-effects model with patient as a random intercept and response as a covariate (mixedModelDE, GeomxTools R package). The mixedModelDE function was modified to output model diagnostics for each gene (isSingular, lme4 R package). Genes with singular fits were excluded from downstream analysis. Selected genes (*p* < 0.05) were visualized in a heatmap (ComplexHeatmap R package). Within pepi + ipi + nivo aggregates, response effects were evaluated by a linear model (stats R package) due to the limited sample size. *P* values were corrected for multiple comparisons using Benjamini-Hochberg and selected genes (FDR < 0.10) were displayed in a heatmap.

Gene Ontology Biological Process (GO BP) enrichment analysis was conducted using the fgsea package. Genes were converted to Entrez ID (clusterProfiler R package and org.Hs.eg.db) and those mapped were ranked using a signed significance metric (−log10 p × sign of effect size), integrating statistical strength and directionality. Enrichment significance was assessed using permutation-based FGSEA, and pathways with FDR < 0.05 were considered significant. Significant pathways were grouped into higher-order functional categories, and representative pathways were selected for visualization using bubble and bar plots (ggplot2 R package).

#### TCR diversity analysis

TCR repertoire diversity was quantified using the Gini coefficient and D50 index calculated from background-subtracted TCR probe counts. The Gini coefficient measured TCR expression inequality within each ROI, with higher values indicating greater clonal dominance. The D50 index quantified clonal concentration as the number of TCR genes required to account for 50% of total TCR expression, with lower values indicating increased clonality. TCR measures were displayed descriptively as box-and-whisker plots (ggplot2 R package).

#### Cytotoxic lymphocyte scores

Abundance of cytotoxic lymphocytes was estimated using MCP-counter (MCPcounter R package) on Q3-normalized counts. Scores within the pepi + ipi + nivo arm were shown as box-and-whisker plots (ggplot2 R package) and are presented descriptively.

#### BCR and TCR sequencing

Formalin-fixed paraffin embedded tissues were sent to Adaptive Biotechnologies, Inc. for total DNA isolation and for Illumina sequencing of the TCR and BCR gene in each sample. Data was analyzed using the ImmunoSEQ Analyzer (Adaptive Biotechnologies, Inc.) and R program immunarch (https://immunarch.com/).

#### Human cytokine/chemokine 96-Plex Discovery Assay

Plasma from patient blood was collected on day 1, day 21 and day of surgery and was then stored at −80 °C prior to analysis using the Eve Technologies 96-plex human cytokine/chemokine Array Discovery Assay. The multiplexing analysis was performed using the Luminex™ 200 system (Luminex, Austin, TX, USA) by Eve Technologies Corp. (Calgary, Alberta). Ninety-six markers were simultaneously measured in the samples using Eve Technologies’ Human Cytokine 96-Plex Discovery Assay® which consists of two separate kits: the Panel A 48-plex and the Panel B 48-plex (Millipore Sigma, Burlington, Massachusetts, USA). The assay was run according to the manufacturer’s protocol. The Panel A 48-plex consisted of sCD40L, EGF, Eotaxin, FGF-2, FLT-3 Ligand, Fractalkine, G-CSF, GM-CSF, GROα, IFN-α2, IFN-γ, IL-1α, IL-1β, IL-1RA, IL-2, IL-3, IL-4, IL-5, IL-6, IL-7, IL-8, IL-9, IL-10, IL-12(p40), IL-12(p70), IL-13, IL-15, IL-17A, IL-17E/IL-25, IL-17F, IL-18, IL-22, IL-27, IP-10, MCP-1, MCP-3, M-CSF, MDC, MIG/CXCL9, MIP-1α, MIP-1β, PDGF-AA, PDGF-AB/BB, RANTES, TGFα, TNF-α, TNF-β, and VEGF-A. The Panel B 48-plex consisted of 6CKine, APRIL, BAFF, BCA-1, CCL28, CTACK, CXCL16, ENA-78, Eotaxin-2, Eotaxin-3, GCP-2, Granzyme A, Granzyme B, HMGB1, I-309, I-TAC, IFNβ, IFNω, IL-11, IL-16, IL-20, IL-21, IL-23, IL-24, IL-28A, IL-29, IL-31, IL-33, IL-34, IL-35, LIF, Lymphotactin, MCP-2, MCP-4, MIP-1δ, MIP-3α, MIP-3β, MPIF-1, Perforin, sCD137, SCF, SDF-1, sFAS, sFASL, TARC, TPO, TRAIL, and TSLP. Assay sensitivities of these markers range from 0.05 to 100 pg/mL for the 96-plex. Samples were run in duplicates and compared to a standard curve for each analyte.

#### ROSALIND® nanostring gene expression analysis

Nanostring gene expression analysis was performed as described^30^ specifically as noted in that paper “Data was analyzed by ROSALIND® (https://rosalind.bio/), with a HyperScale architecture developed by ROSALIND, Inc. (San Diego, CA). Read Distribution percentages, violin plots, identity heatmaps, and sample MDS plots were generated as part of the QC step. Normalization, fold changes and *p*-values were calculated using criteria provided by Nanostring. ROSALIND® follows the nCounter® Advanced Analysis protocol of dividing counts within a lane by the geometric mean of the normalizer probes from the same lane. Housekeeping probes to be used for normalization are selected based on the geNorm algorithm as implemented in the NormqPCR R library1. Abundance of various cell populations is calculated on ROSALIND using the Nanostring Cell Type Profiling Module. ROSALIND performs a filtering of Cell Type Profiling results to include results that have scores with a *p*-value greater than or equal to 0.05. Fold changes and *p* values were calculated using the fast method as described in the nCounter® Advanced Analysis 2.0 User Manual. *P*-value adjustment was performed using the Benjamini-Hochberg method of estimating false discovery rates (FDR). Clustering of genes for the final heatmap of differentially expressed genes was done using the PAM (Partitioning Around Medoids) method using the fpc R library2 that takes into consideration the direction and type of all signals on a pathway, the position, role and type of every gene, etc. Hypergeometric distribution was used to analyze the enrichment of pathways, gene ontology, domain structure, and other ontologies. The topGO R library3, was used to determine local similarities and dependencies between GO terms in order to perform Elim pruning correction. Several database sources were referenced for enrichment analysis, including Interpro4, NCBI5, MSigDB6,7, REACTOME8, WikiPathways9. Enrichment was calculated relative to a set of background genes relevant for the experiment. Heatmaps were generated in RStudio (4.4.1) using gplots (3.2.0) and heatmap.2x (0.0.0.9000).”

#### Survival analysis

Survival analyses were performed in R (v4.3.0) using the survival and survminer packages. Kaplan–Meier curves were generated, and optimal LA high/low cutoffs were determined using the surv cutpoint function in survminer.

### Reporting summary

Further information on research design is available in the Nature Portfolio Reporting Summary linked to this article.

## Supplementary information

Supplementary Information

Reporting Summary

Transparent Peer Review file

## Source data

Source Data

## Data Availability

The datasets generated during and/or analyzed during the current study for clinical trial NCT03769155 are available on NCBI Gene Omnibus, Emory cohort: GSE332987, PRJNA1469599, MDACC cohort: GSE331322, PRJNA1467675. The Adaptive BCR and TCR sequencing data have been deposited in the ImmuneACCESS database (Adaptive Biotechnologies) under https://doi.org/10.21417/ATR2026S and URL https://clients.adaptivebiotech.com/pub/ruffin-2026-s and are also available on NCBI Gene Omnibus, GSE347762 Nanostring n counter raw and normalized counts are available within the source data file. Given the small cohort size individual de-identified participant data will not be published to protect patient privacy; however, clinical data will be available from the corresponding author upon request. The study protocol is available in the Supplementary Information. The remaining data are available within the Article, Supplementary Information or Source Data file and/or from the corresponding authors upon request. Source data are provided with this paper.
