# Supplementary material for: Neoadjuvant pepinemab in combination with nivolumab and/or ipilimumab in resectable metastatic melanoma: a pilot biomarker-driven trial
Source: Nat Commun. 2026 Oct 6;17:11377. doi: 10.1038/s41467-026-78231-3 (PMC13736988; doi:10.1038/s41467-026-78231-3)
Supplement: Supplementary file 1 [file 41467_2026_78231_MOESM1_ESM.pdf]

## Supplementary Information for

# Neoadjuvant pepinemab in combination with nivolumab and/or ipilimumab in resectable metastatic melanoma: a pilot biomarker-driven trial

**Authors:** Ayana T. Ruffin[1*], Crystal L. Mallow[2*], Brian M. Olson[3], Jacklyn Hammons[3], Brenda Melendez[4], Manoj Chelvanambi[4], Elaine M. Gersz[2], Megen C. Wittling[1], Laurence P. Diggs[4], Christine Reilly[2], Jeffrey M. Switchenko[5], Alexander J. Lazar[4], Shannon K. Swisher[1], Jayden Kim[3], Brian Burns[6], Agnes Harutyunyan[6], Terrence L. Fisher[2], Keith A. Delman[1], Melinda L. Yushak[3], Douglas C. Parker[7], David Lawson[1], Jennifer A. Wargo[4], Maurice Zauderer[2], Elizabeth E. Evans[2], Chrystal M. Paulos[1,8**], Michael C. Lowe[1**], Gregory B. Lesinski[3**]

**Affiliations:**

[1] Department of Surgery, Winship Cancer Institute of Emory University, Atlanta, GA, USA
[2] Vaccinex, Inc. Rochester, NY, USA
[3] Department of Hematology and Medical Oncology, Winship Cancer Institute of Emory University Atlanta, GA, USA
[4] MD Anderson Cancer Center, Houston, TX, USA
[5] Department of Biostatistics and Bioinformatics, Rollins School of Public Health of Emory University Atlanta, GA USA
[6]Winship Cancer Institute of Emory University, Atlanta, GA, USA
[7] Department of Pathology, Winship Cancer Institute of Emory University, Atlanta, GA, USA
[8] Department of Microbiology and Immunology, Winship Cancer Institute of Emory University, Atlanta, GA, USA

* These authors contributed equally to this work

**These authors jointly supervised this work

This file includes:

1. Supplementary Figure 1: Pathologic responses
2. Supplementary Figure 2: Patient enrollment and Consort sample diagram
3. Supplementary Figure 3: Transcriptional and pathway enrichment
4. Supplementary Figure 4: Lymphoid aggregate distribution and spatial grading
5. Supplementary Figure 5: Flow cytometry gating strategy
6. Supplementary Figure 6: Spatial transcriptomic characterization
7. Supplementary Figure 7: BCR and TCR clonotypes
8. Supplementary Figure 8: Gene usage comparison
9. Supplementary Figure 9: Plasma cytokine, chemokine and growth factor analysis
10. Supplementary Figure 10: PBMC analysis
11. Supplementary Table 1: Flow cytometry antibody panel
12. Supplementary Table 2: Immunohistochemistry antibody panel
13. Supplementary Table 3: Individual figure denominators
14. Supplementary Table 4: Grade specific toxicities summary
15. Supplementary Table 5: MDACC demographics
16. Supplementary Note: Clinical Trial protocol

a

| Cohort | Therapy | N | pCR | mPR |
|---|---|---|---|---|
| A | pepinemab / nivolumab | 8 | 2/8, 25.0% (95% CI 3.2%-65.1%) | 3/8, 37.5% (95% CI 8.5%-75.5%) |
| B | pepinemab / ipilimumab | 8 | 1/8, 12.5% (95% CI 0.3%-52.7%) | 1/8, 12.5% (95% CI 0.3%-52.7%) |
| C | pepinemab / ipilimumab / nivolumab | 8 | 5/8, 62.5% (95% CI 24.5%-91.5%) | 6/8, 75.0% (95% CI 34.9%-96.8%) |
| D | nivolumab | 8 | 2/8, 25.0% (95% CI 3.2%-65.1%) | 3/8, 37.5% (95% CI 8.5%-75.5%) |

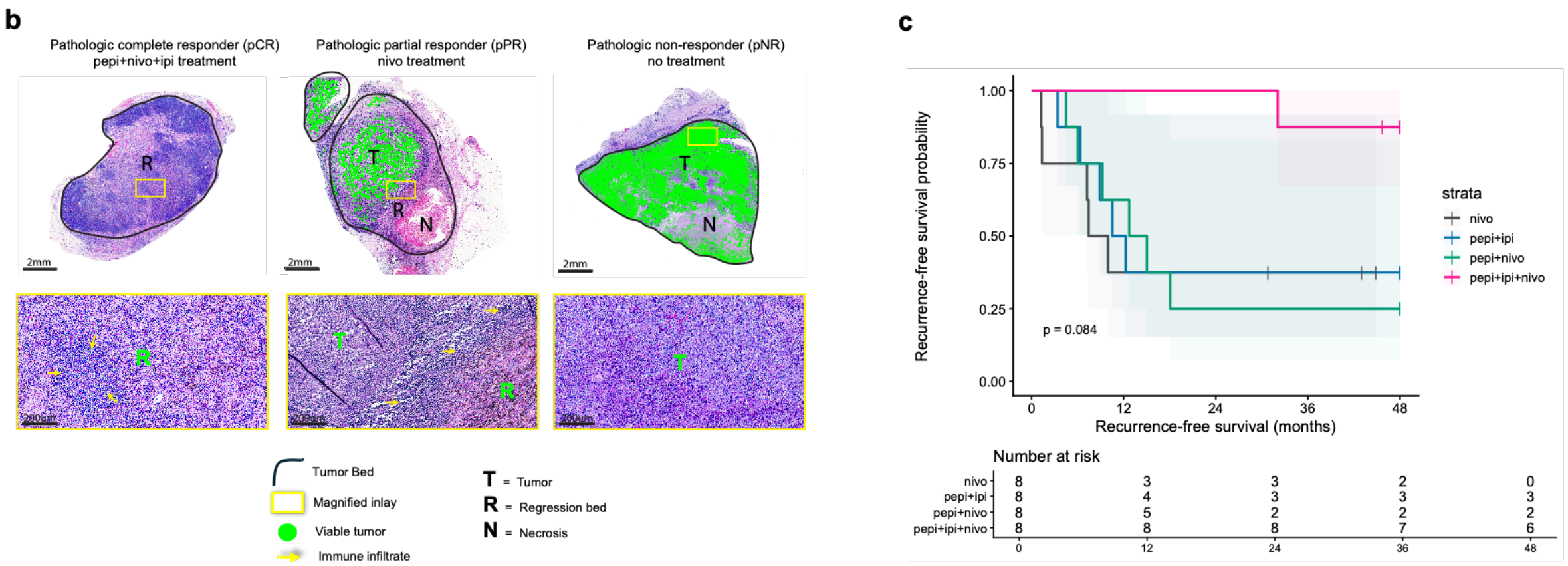


## Supplementary Figure 1: Pathologic responses

**(a)** Summary of pathologic responses for 32 patients who were treated with pepinemab plus nivolumab and/or ipilimumab. Report n/N, percentage, and two-sided 95% CI using exact binomial (Clopper-Pearson). **(b)** Representative images showing pathological complete responder (pCR), partial responder (pPR) and non-responder (pNR). Tumor bed was defined by pathologist as non-normal lymph node architecture including viable tumor cells, regression bed and necrosis. Immune infiltrate pockets are demarcated with an arrow in the magnified inlays. **(c)** Kaplan Meier recurrence-free survival analysis stratified by treatment arm. Recurrence-free survival plotted to 48 months. Data analyzed by Log-Rank (Mantel-Cox) test. Shaded regions represent 95% confidence intervals. Number-at-risk table displayed at 0, 12, 24, 36, and 48 months. Abbreviations: pepinemab (pepi), nivolumab (nivo), ipilimumab (ipi).

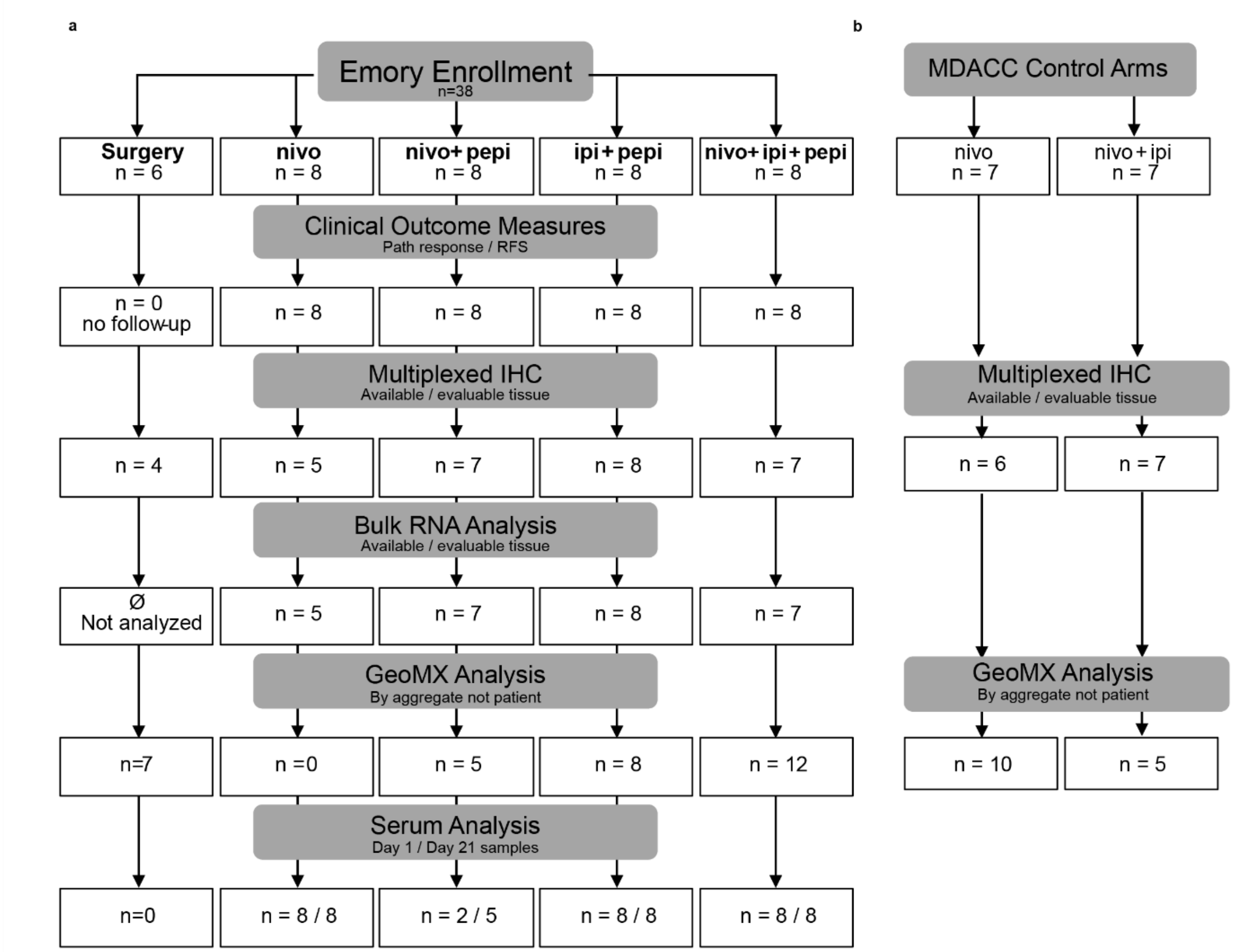


## Supplementary Figure 2 : Patient enrollment and analytical sample availability

**(a)** CONSORT diagram showing patient enrollment in the Emory neoadjuvant melanoma study by treatment cohort and the number of samples available for each analytical platform. Sample numbers for multiplex immunohistochemistry (mIHC) analyses may vary owing to tissue loss during sequential staining rounds. GeoMx Digital Spatial Profiling was performed at the lymphoid aggregate level rather than the patient level; no lymphoid aggregates meeting inclusion criteria were identified in the nivolumab-only cohort.

**(b)** CONSORT diagram showing tissue accrual in the MD Anderson Cancer Center (MDACC) biologic comparator cohort and the number of samples available for each analytical platform.Abbreviations: pepinemab (pepi), nivolumab (nivo), ipilimumab (ipi).

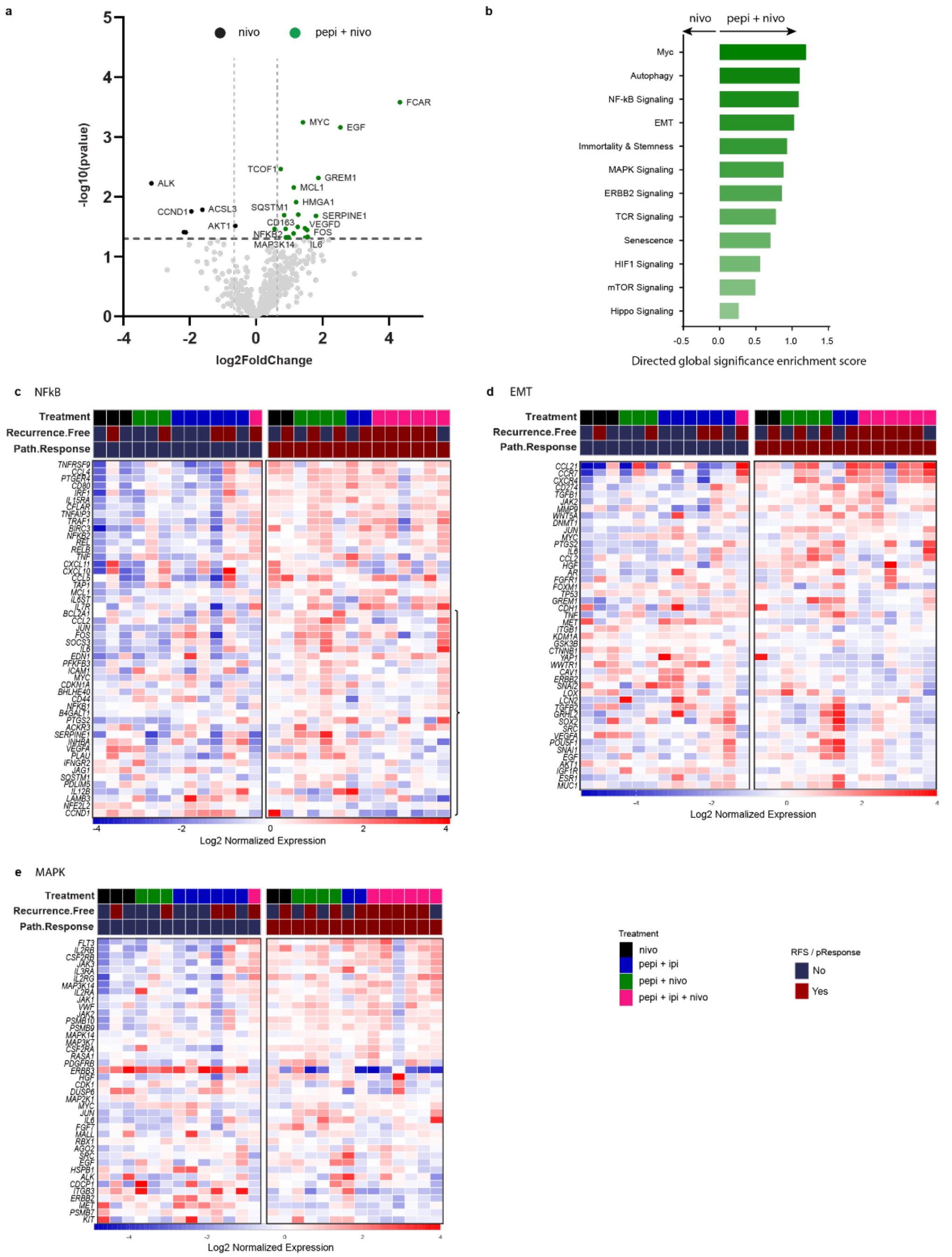


## Supplementary Figure 3: Transcriptional and pathway enrichment

**(a)** Volcano plot depicting differentially expressed genes between nivolumab (nivo) and pepinemab plus nivolumab (pepi+nivo) treatment groups. Nanostring ncounter data analyzed by ROSALIND®. (**b)** Pathway enrichment analysis depicting pathways enriched in pepi + nivo treatment group compared to nivo alone. (**c)** Heatmaps of genes enriched in pathways with the greatest number of differential genes; NFκB **(c**), EMT **(d)** and MAPK **(e).** Pathways stratified by treatment group and pathological response.Abbreviations: pepinemab (pepi), nivolumab (nivo), ipilimumab (ipi), Responders: pathologic complete response (pCR), major pathologic response (mPR), and partial pathologic response (pPR). Non-responders: pathologic non-response (pNR). N values for each group can be found in supplementary table 3.

a

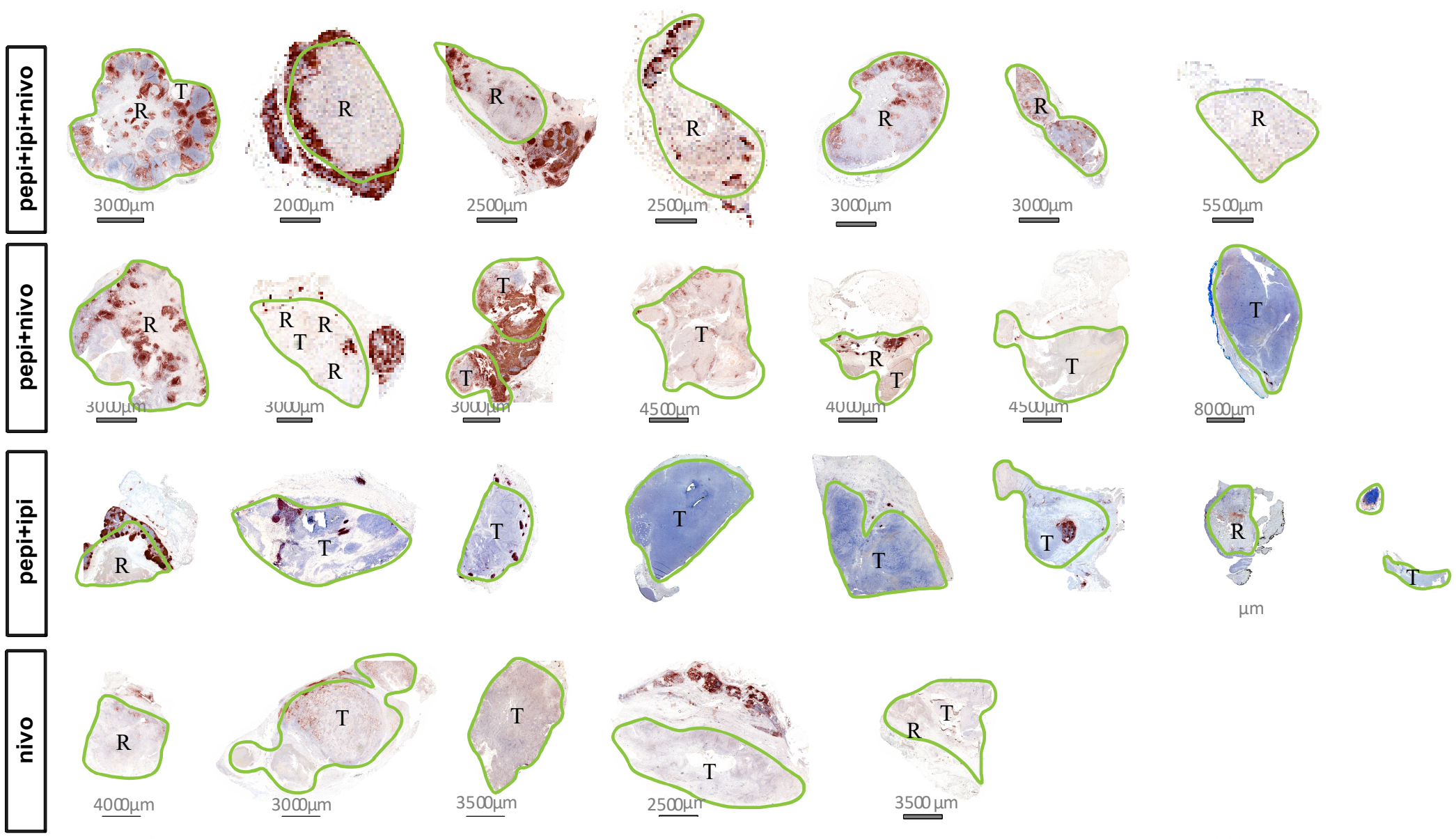


b

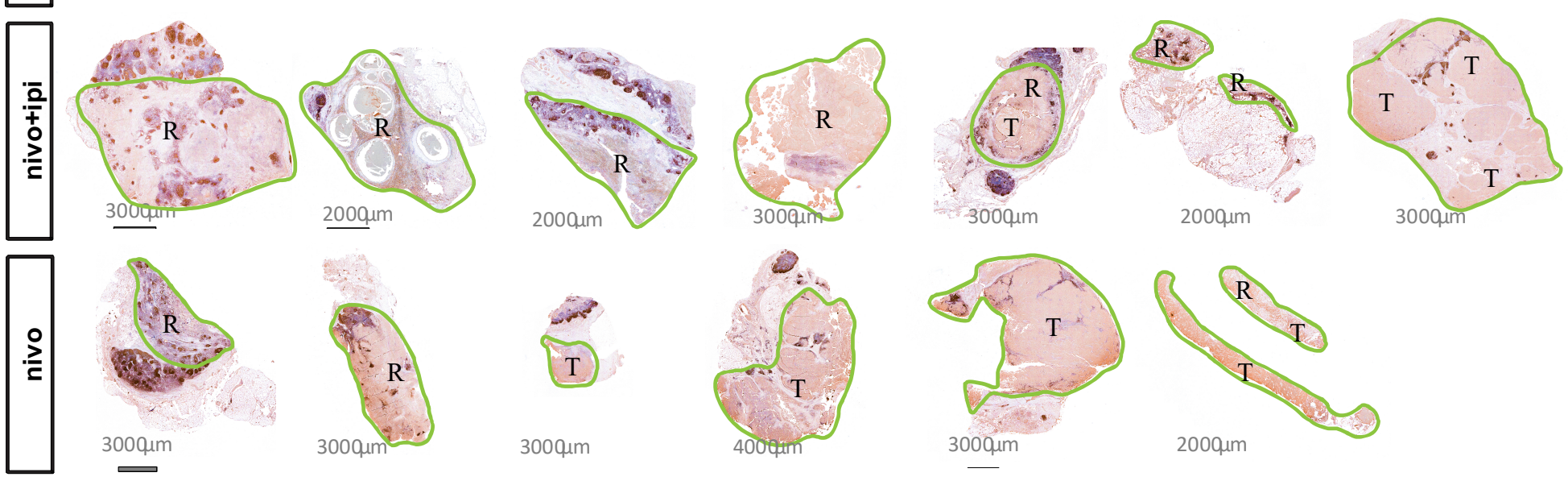


c

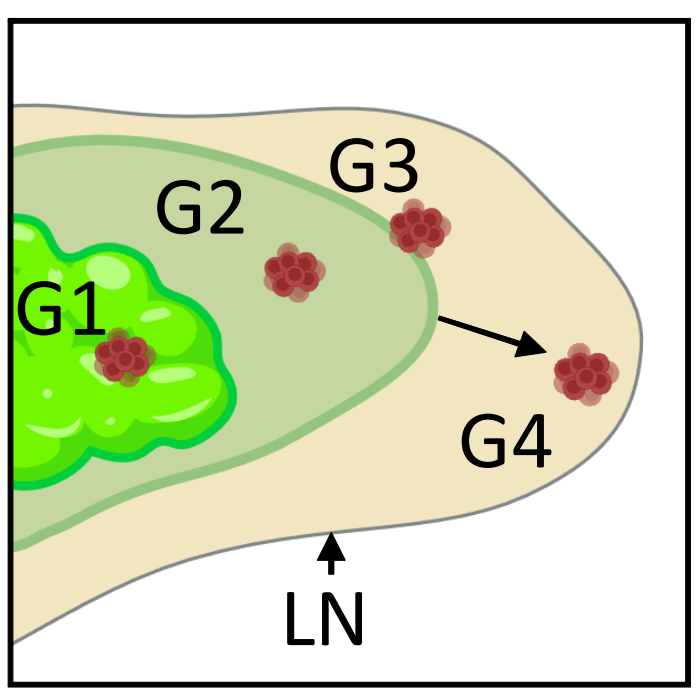


| Grade | Aggregate location | Morphologically resembles normal LN |
|---|---|---|
| G1 | Within tumor island | No |
| G2 | Within tumor involved region | No |
| G3 | Tumor bed adjacent | Questionable |
| G4 | Distal from tumor involved region | Yes |

## Supplementary Figure 4: Lymphoid aggregate distribution and spatial grading

**(a)** Single-plex immunohistochemistry images of CD20 staining from melanoma tumor resections in the Emory cohort, organized by treatment group. (**b**) Single-plex immunohistochemistry images of CD20 staining from melanoma tumors in the MD Anderson Cancer Center (MDACC) biologic comparator cohort. Green outlines indicate the tumor bed boundary. Regression bed and tumor bed regions are denoted by R and T, respectively. **(c)** Schematic illustrating the lymphoid aggregate (LA) spatial grading system used for multiplex immunohistochemistry (mIHC) and GeoMx Digital Spatial Profiling analyses. Created in BioRender; Evans, E. (2026) https://BioRender.com/ wmztx1i. Abbreviations: pepinemab (pepi), nivolumab (nivo), ipilimumab (ipi), regression bed (R), Tumor bed (T). N values can be found in supplementary table 3.

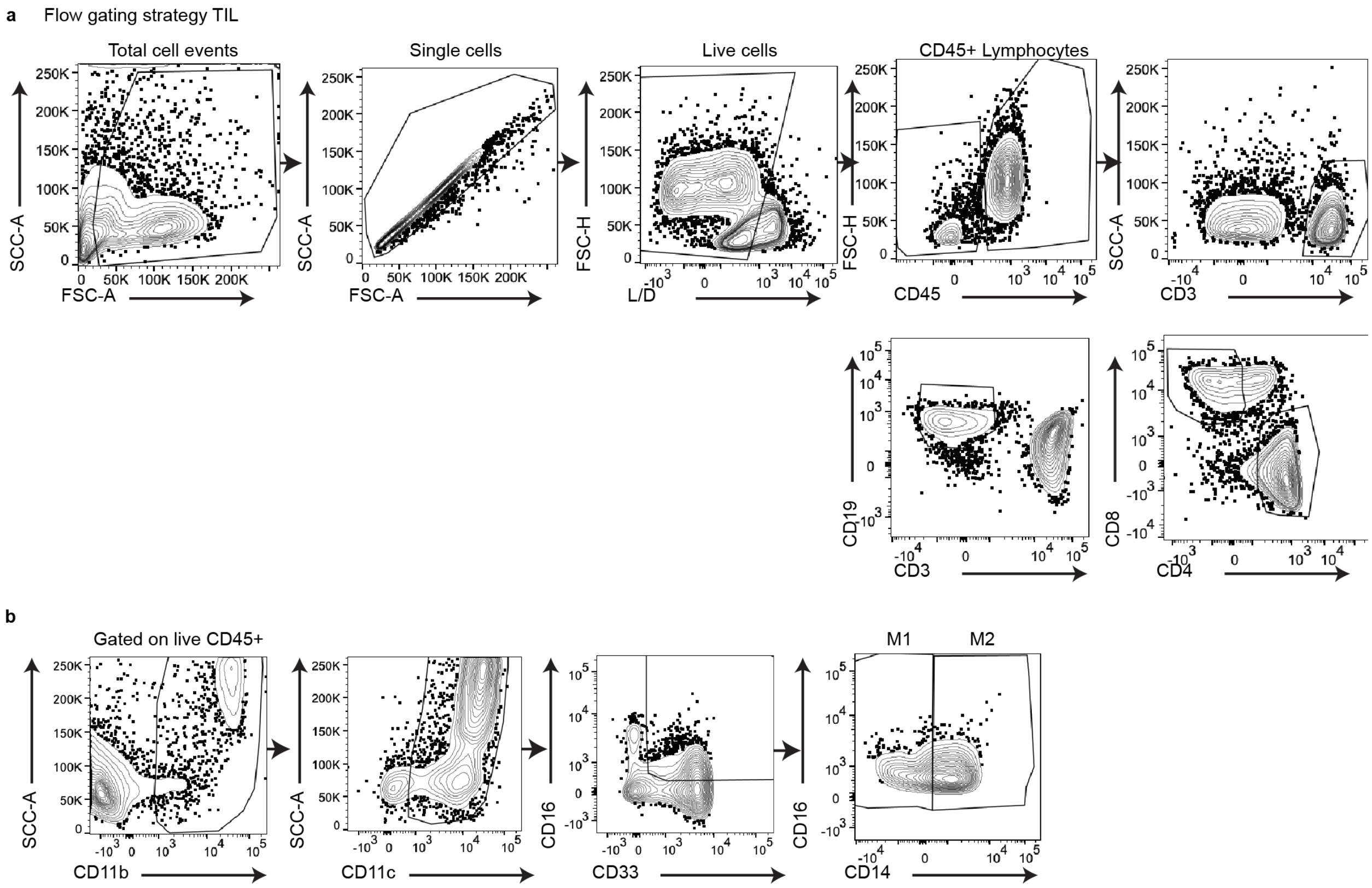


## Supplementary Figure 5: Flow cytometry gating strategy

**(a)** Representative flow cytometry gating strategy for TIL cells and **(b)** myeloid cells. Panel a) is quantified in main Figure 3g. Panel b) is quantified in main figure 3 and d

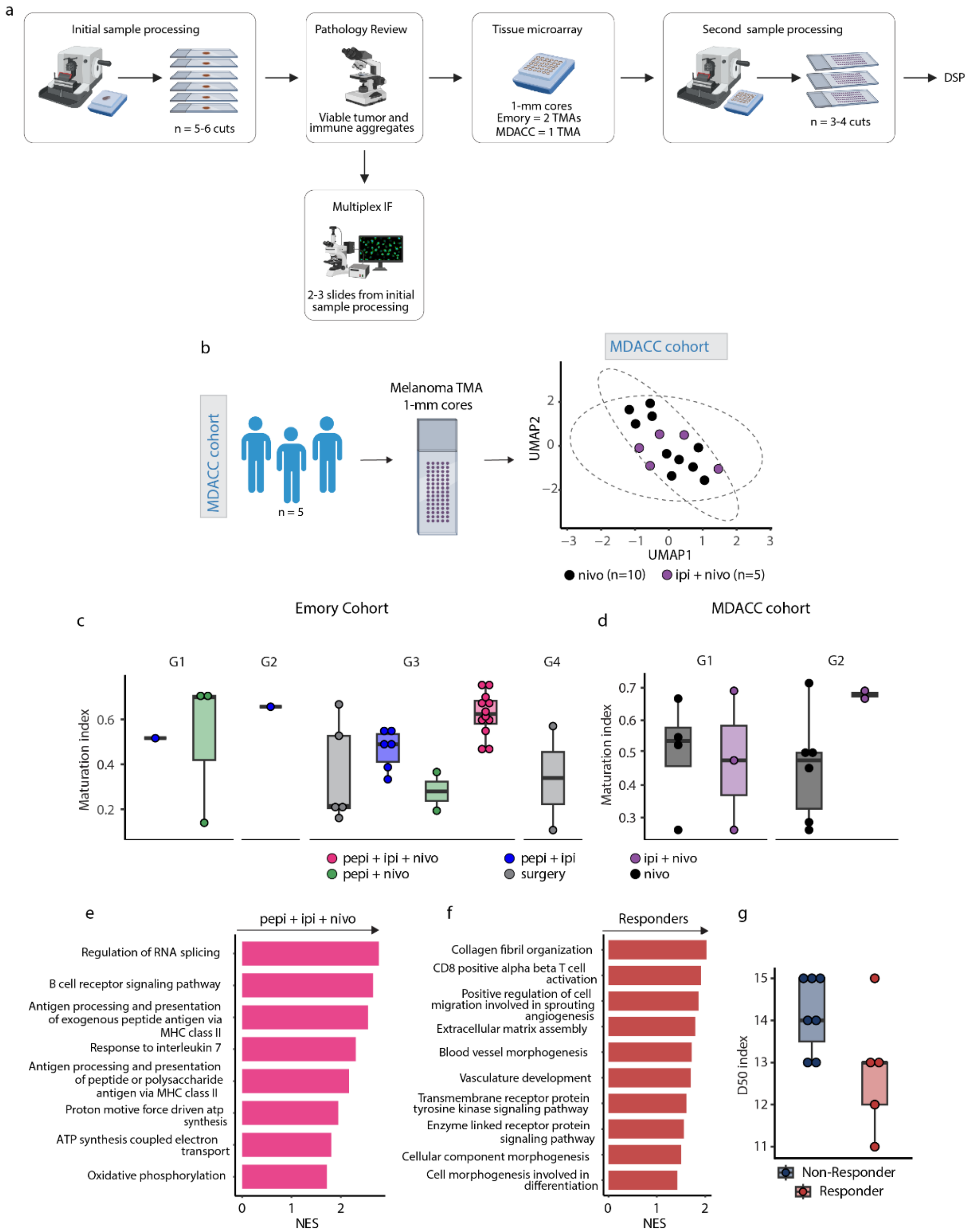


4

## Supplementary Figure 6: Spatial transcriptomic characterization of lymphoid aggregate maturation and immune activation.

**(a)** Workflow schematic of tissue processing and GeoMx digital spatial profiling. **(b)** $CD45^{+}CD20^{+}$ lymphoid aggregate selection and GeoMx digital spatial profiling workflow in the MD Anderson Cancer Center (MDACC) biologic comparator cohort. **(c)** Rank-based maturation index score across immune aggregate grades (G1-G4) in the Emory cohort (surgery, 2 patients/7 ROIs; pepi + ipi, 3 patients/8 ROIs; pepi + nivo, 2 patients/5 ROIs; pepi + ipi + nivo, 3 patients/12 ROIs). **(d)** Rank-based maturation index across immune aggregate grades (G1-G2) in the MDACC cohort presented descriptively (ipi + nivo, 2 patients/5 ROIs; nivo, 3 patients/10 ROIs). **(e)** Selected Gene Ontology biological process pathways enriched in G3 immune aggregates in the Emory cohort, highlighting immune, metabolic, and RNA transcriptional programs summarized in the main figure (FDR <0.05)**. (f)** Representative Gene Ontology biological process pathways

enriched in G3 immune aggregates from pepi + ipi + nivo-treated patients, comparing responders (2 patients/5 ROIs) versus non-responders (1 patient/7 ROIs) and reflecting structural and extracellular matrix remodeling, vascular remodeling, growth factor signaling, developmental processes, and adaptive immune activation summarized in the main figure (FDR < 0.05). **(g)** TCR diversity in G3 immune aggregates from pepi + ipi + nivo responders (2 patients/5 ROIs) versus non-responders (1 patient/7 ROIs), quantified by the D50 index and presented descriptively.For box-and-whisker plots, center lines indicate the median, boxes indicate the interquartile range (IQR), and whiskers extend 1.5 x IQR. Abbreviations: pepinemab (pepi), nivolumab (nivo), ipilimumab (ipi). Responders: pathologic complete response (pCR), major pathologic response (mPR), and partial pathologic response (pPR). Non-responders: pathologic non-response (pNR).Panels a and b Created in Biorender. Melendez, B. (2025) https://BioRender.com/nzw77wz

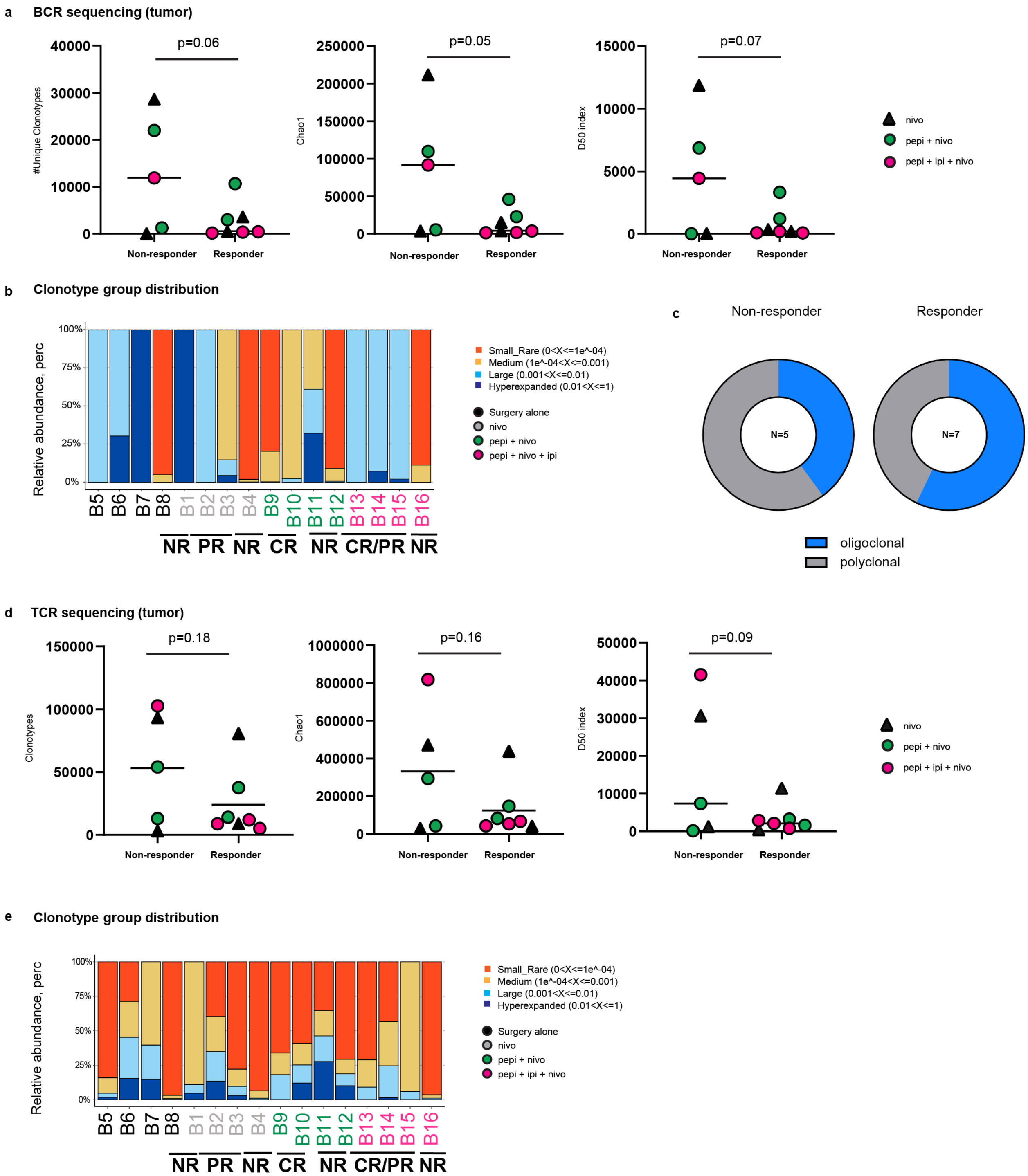


## Supplementary Figure 7: BCR and TCR clonotypes

**(a)** Quantification of number of unique BCR clonotypes, chao 1 value and D50 index grouped by response. Responders: pathologic complete response (pCR), major pathologic response (mPR), and partial pathologic response (pPR). Non-responders: pathologic non-response (pNR). Each dot is an individual patient. Data analyzed by immunoSEQ (**b)** Stacked bar graph depicting distribution of four clonotype groups (defined based on relative abundance of the clone sequences) for BCR clonotypes. Patient response status is labeled under patient identifier. . Non-responder (NR), Partial response (PR), Complete response (CR) .**(c)** Donut graph depicting summary of the proportion of patients with oligoclonal (the combined proportion

of large and hyperexpanded clonotypes >25%) sequences in each response group. (**d)** Quantification of number of unique TCR clonotypes, chao 1 value and D50 index grouped by response. **(e)** Stacked bar graph depicting distribution of four clonotype groups (defined based on relative abundance of the clone sequences) for TCR clonotypes. Data analyzed by unpaired T- test. Abbreviations: pepinemab (pepi), nivolumab (nivo), ipilimumab (ipi). N values can be found in supplementary table 3.

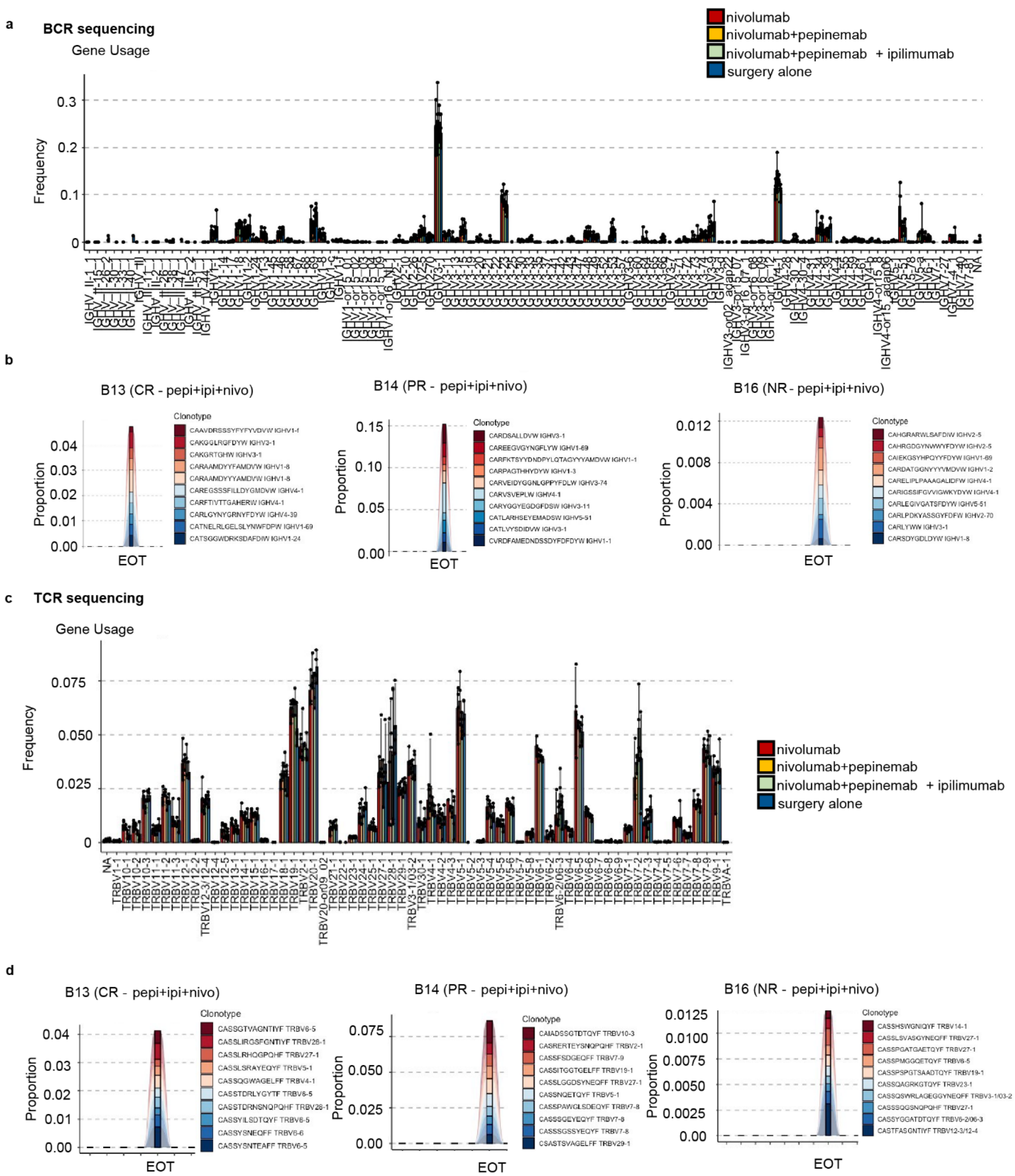
a
BCR sequencing
Gene Usage
nivolumab
nivolumab+pepinemab
nivolumab+pepinemab + ipilimumab
surgery alone
Frequency
b
B13 (CR - pepi+ipi+nivo)
B14 (PR - pepi+ipi+nivo)
B16 (NR - pepi+ipi+nivo)
Clonotype
Proportion
EOT
c
TCR sequencing
Gene Usage
Frequency
nivolumab
nivolumab+pepinemab
nivolumab+pepinemab + ipilimumab
surgery alone
d
B13 (CR - pepi+ipi+nivo)
B14 (PR - pepi+ipi+nivo)
B16 (NR - pepi+ipi+nivo)
Clonotype
Proportion
EOT

## Supplementary Figure 8: Gene usage comparison

**(a)** IGHV gene usage comparison. The x-axis depicts all functional human IGHV alleles while the y-axis is the percentage of IGHV sequences in all repertoires of patients within indicated treatment arm. (**b)** Tracking the relative abundance of top 10 most abundant BCR clonotypes in three patients within the pepi + ipi + nivo treatment arm. Patient response status is indicated next to patient identifier. **(c)** Vβ gene usage comparison. The x-axis depicts all functional human TRBV alleles while the y-axis is the percentage of TCRβ sequences in all repertoires of patients within indicated treatment arm using each TRBV allele. **(d)** Tracking the relative abundance of top 10 most abundant TCR clonotypes in three patients within the pepi + ipi + nivo treatment arm. Patient response status is indicated next to patient identifier. Abbreviations: pepinemab (pepi), nivolumab (nivo), ipilimumab (ipi), End of treatment (EOT). N values can be found in supplementary table 3.

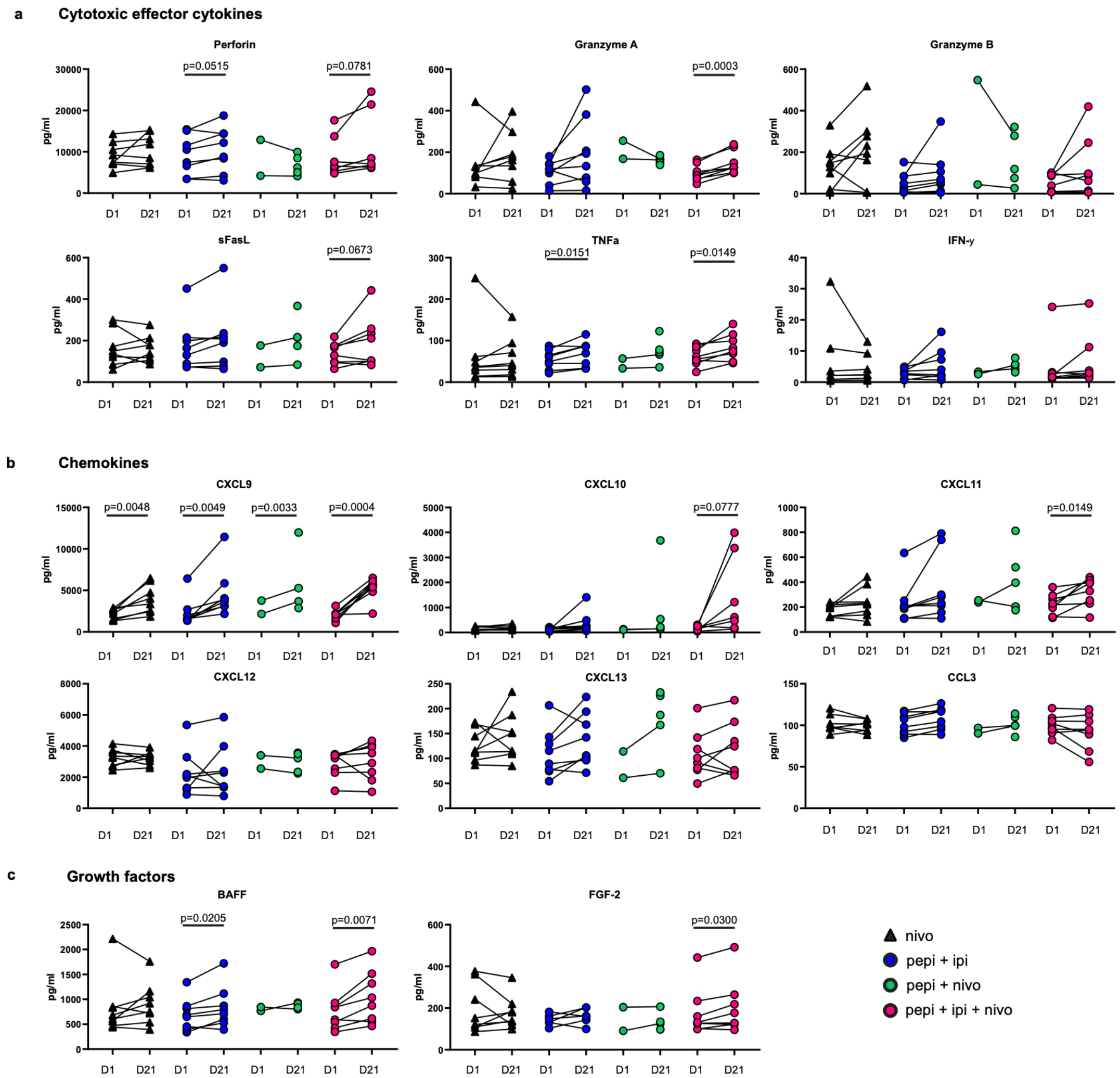


## Supplementary Figure 9: Plasma cytokine, chemokine and growth factor analysis

**(a)** Quantification of plasma levels of cytotoxic effector cytokines perforin, granzyme A, granzyme B, soluble Fas ligand (sFasL), tumor necrosis factor α (TNFα) and interferon gamma (IFN-γ) at baseline Day 1 (D1) and Day 21 (D21). **(b)** Quantification of plasma levels of chemokines CXCL9, CXCL10, CXCL11, CXCL12, CXCL13 and CCL3 at baseline Day 1 (D1) and Day 21 (D21). **(c)** Quantification of growth factors BAFF and FGF-2 at baseline Day 1 (D1) and Day 21. Data analyzed by paired T test between D1 and D21 for each analyte within each treatment group. *p<0.05. **p<0.01, ***p<0.001 . N values can be found in supplementary table 3.Abbreviations: pepinemab (pepi), nivolumab (nivo), ipilimumab (ipi).

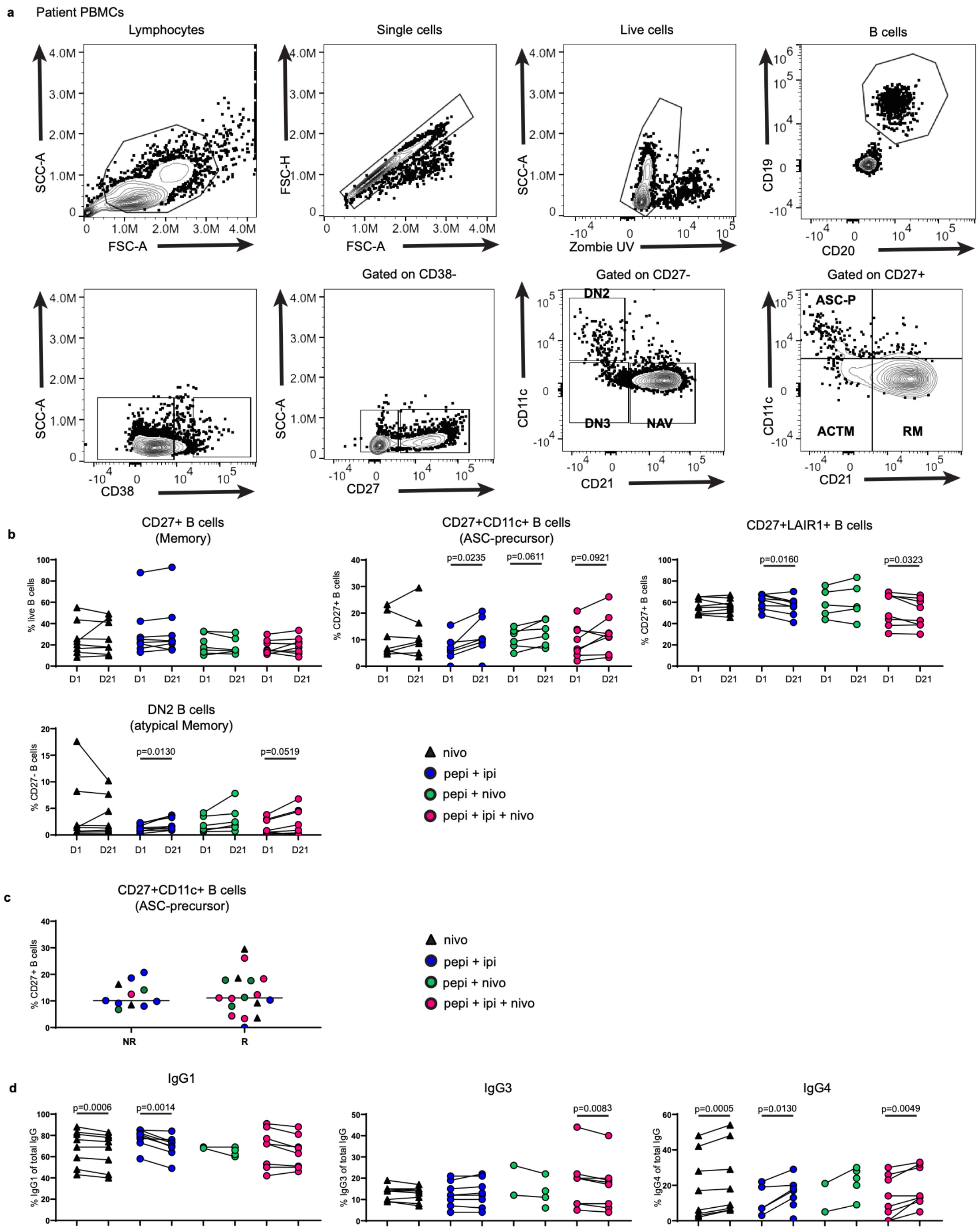

a
Patient PBMCs
Lymphocytes
Single cells
Live cells
B cells
SCC-A
FSC-A
FSC-H
Zombie UV
CD19
CD20
CD38
CD27
Gated on CD38-
Gated on CD27-
Gated on CD27+
DN2
DN3
NAV
CD11c
CD21
ASC-P
ACTM
RM
b
CD27+ B cells
(Memory)
% live B cells
CD27+CD11c+ B cells
(ASC-precursor)
% CD27+ B cells
p=0.0235
p=0.0611
p=0.0921
CD27+LAIR1+ B cells
p=0.0160
p=0.0323
DN2 B cells
(atypical Memory)
% CD27- B cells
p=0.0130
p=0.0519
D1
D21
nivo
pepi + ipi
pepi + nivo
pepi + ipi + nivo
c
CD27+CD11c+ B cells
(ASC-precursor)
% CD27+ B cells
NR
R
nivo
pepi + ipi
pepi + nivo
pepi + ipi + nivo
d
IgG1
% IgG1 of total IgG
p=0.0006
p=0.0014
IgG3
% IgG3 of total IgG
p=0.0083
IgG4
% IgG4 of total IgG
p=0.0005
p=0.0130
p=0.0049
D1
D21

## Supplementary Figure 10: PBMC analysis

**(a)** Representative flow cytometry gating strategy of B cell populations in patient PBMCs. **(b)** Quantification of $CD27^+$ memory B cells, $CD27^+CD11c^+$ B cells, $CD27^+$ $LAIR1^+$ B cells, $CD27^-CD11c^+$ B cells (DN2) at baseline (D1) and Day 21 (D21) post treatment. **(c)** Quantification of $CD27^+CD11c^+$ B cells grouped by response. **(d)** Quantification of IgG1, IgG3 and IgG4 in patient plasma at baseline and Day 21 post treatment. Data analyzed by paired T test between D1 and D21 for each analyte within each treatment group. . *p<0.05. **p<0.01, ***p<0.001. N values can be found in supplementary table 3.

Supplementary Table 1

| PANEL | MARKER | COLOR | DILUTION | Vendor | Catalog Number |
|---|---|---|---|---|---|
| Master Mix | VX15-2503 | unconjugated | 20μg/mL | | |
| | anti-chicken IgY | Biotin | 10μg/mL | Southern Biotech | 8320-08 |
| | anti-human IgG4 | Biotin | 10μg/mL | Southern Biotech | 9200-08 |
| | PlexinB1 | unconjugated | 1μg/mL | R&D Systems | MAB37491 |
| | Live/Dead | Ghost Dye 780 | 1:1000 | Tonbo Biosciences | 13-0865-T100 |
| | CD45 | Pe-Cy7 | 1:100 | Biolegend | 368532 |
| | anti-mouse CD8 | FITC | 1:100 | Biolegend | 1007806 |
| Lymph | streptavidin-APC | | 1:5 | BD Biosciences | 349024 |
| | CD3 | BV711 | 1:100 | Biolegend | 344838 |
| | CD4 | AF700 | 1:100 | Biolegend | 344622 |
| | CD8 | BV650 | 1:100 | Biolegend | 301041 |
| | CD19 | PerCP-Cy5.5 | 1:100 | Biolegend | 302320 |
| | CD56 | BUV395 | 1:100 | BD Biosciences | 563554 |
| | CD38 | BV605 | 1:100 | Biolegend | 303532 |
| | HLA-DR | BV785 | 1:100 | Biolegend | 307642 |
| | CD26 | PECy5 | 1:100 | Biolegend | 302708 |
| | PD1 | BV421 | 1:100 | Biolegend | 367421 |
| | Tim3 | BV510 | 1:100 | Biolegend | 345030 |
| | IgG | PE | 1:100 | Biolegend | 400114 |
| | LAG3 | PE | 1:100 | Biolegend | 369306 |
| | Ki67 | PE-Dazzle594 | 1:100 | Biolegend | 350534 |
| Treg | streptavidiin-APC | | 1:5 | | |
| | CD3 | BV711 | 1:100 | | |
| | CD4 | AF700 | 1:100 | | |
| | GITR | BV605 | 1:100 | Biolegend | 371214 |
| | CD127 | BV610 | 1:100 | Biolegend | 351332 |
| | CD25 | PE | 1:100 | Biolegend | 356104 |
| | FoxP3 | BV421 | 1:100 | Biolegend | 320124 |
| Myeloid | CD11b | PeCy5 | 1:100 | Biolegend | 101210 |
| | CD11c | BV421 | 1:100 | Biolegend | 337226 |
| | CD14 | PerCP-Cy5.5 | 1:100 | Biolegend | 325622 |
| | CD15 | Pe-Dazzle594 | 1:100 | Biolegend | 323038 |
| | CD16 | AF700 | 1:100 | Biolegend | 302026 |
| | CD33 | BV785 | 1:100 | Biolegend | 303428 |
| | CD40 | BV650 | 1:100 | Biolegend | 334338 |
| | HLA-DR | BV510 | 1:100 | Biolegend | 307646 |
| | CD103 | BV605 | 1:100 | Biolegend | 350218 |
| | anti-mouse F(ab) | APC | 1:100 | R&D Systems | F0101B |
| | IgG | PE | 1:100 | | |
| | IgG | BV711 | 1:100 | Biolegend | 400354 |
| | PlexinB2 | PE | 1:100 | R&D Systems | FAB53291P |
| | PDL1 | BV711 | 1:100 | Biolegend | 329722 |
| TCF | CD3 | BV711 | 1:100 | | |
| | CD4 | AF700 | 1:100 | | |
| | CD8 | BV650 | 1:100 | | |
| | PD1 | BV421 | 1:100 | | |
| | TCF1/7 | PE | 1:100 | Cell Signaling | C63D9 |

## Flow cytometry antibody panel

Supplementary Table 2

| MARKER | Clone | Vendor | Catalog Number | DILUTION |
|---|---|---|---|---|
| CD20 | L26 | Thermo | MA513141 | 1:100 |
| CD4 | EPR6855 | Abcam | ab133616 | 1:100 |
| FoxP3 | 236A/E7 | Thermo | 14-4777-82 | 1:40 |
| CD8 | C8/144B | Thermo | MA513473 | 1:50 |
| CD21 | 2G9 | Cell Signaling | 76069S | 1:100 |
| CD23 | E1R4G | Cell Signaling | 87960 | 1:800 |
| CXCR5 | D6L3C | Cell Signaling | 72172S | 1:100 |
| Sema4D | EP3569 | Abcam | ab134128 | 6ug/ml |
| CD11c | EP1347Y | Abcam | ab52632 | 1:500 |
| CD141 | 141C01 | Thermo | MA5-11454 | 1:50 |
| CD68 | D4B9C | Cell Signaling | 76437S | 1:400 |
| HLA-DR | TAL 1B5 | Abcam | ab20181 | 1ug/ml |
| PD-L1 | E1L3N | Cell Signaling | 13684S | 1:200 |
| Sox-10 | EP268 | Cell Marque | 383R-18 | 1:100 |

**Immunohistochemistry antibody panel**

Supplementary Table 3

| | Figure, Panel | Analysis | Surgery | nivo | pepi+ipi | pepi+ nivo | pepi+ipi +nivo | MDACC nivo | MDACC nivo+ipi |
|---|---|---|---|---|---|---|---|---|---|
| Main Figures | 1 | Enrollment | 6 | 8 | 8 | 8 | 8 | NA | NA |
| | 2,a | RNA | 0 | 5 | 8 | 7 | 7 | NA | NA |
| | 2,b | Blood | 0 | 8 | 8 | 5 | 8 | NA | NA |
| | 2,c | RNA | 0 | 5 | 8 | 7 | 7 | NA | NA |
| | 3,a | mIHC | NA | 5 | NA | 7 | NA | NA | NA |
| | 3,d | mIHC | 2 | 4 | 8 | 6 | 7 | 4 | 7 |
| | 3,e | mIHC | 3 | 5 | 8 | 7 | 7 | 5 | 7 |
| | 3,f | mIHC | 4 | 5 | 8 | 7 | 7 | 5 | 7 |
| | 3,g | Flow | 5 | 4 | 7 | 7 | 7 | NA | NA |
| | 4,a | mIHC | NA | 5 | 6 | 6 | 7 | NA | NA |
| | 4,b | mIHC | NA | 5 | 6 | 6 | 7 | NA | NA |
| | 4,c | mIHC | NA | NA | NA | NA | NA | **6** | 7 |
| | 4,d | mIHC | NA | 5 | 6 | 6 | 7 | NA | NA |
| | 4,e | mIHC | NA | NA | NA | NA | NA | **6** | 7 |
| | 5 | GeoMx | 7 | 0 | 8 | 5 | 12 | NA | NA |
| Supplementary Data Figures | Supp 3, a,b | RNA | NA | 5 | NA | 7 | NA | NA | NA |
| | Supp 3, c-e | RNA | NA | 5 | 8 | 7 | 7 | NA | NA |
| | Supp 4 | IHC | NA | 5 | 8 | 7 | 7 | 6 | 7 |
| | Supp 6 | GeoMX | 7 | 0 | 8 | 5 | 12 | 10 | 5 |
| | Supp 7 | BCR/TCR | 4 | 4 | 0 | 4 | 4 | NA | NA |
| | Supp 8 | BCR/TCR | 4 | 4 | 0 | 4 | 4 | NA | NA |
| | Supp 9 | Blood | 0 | 8 | 8 | 5 | 8 | NA | NA |
| | Supp 10 | Blood | 0 | 8 | 8 | 5 | 8 | NA | NA |

## Individual figure denominators.

Assay-specific denominators are reported by treatment arm for both Emory cohorts and MDACC. For each figure, sample sizes (n) and analysis methods are specified by treatment arm. MDACC cohorts are denoted as “MDACC.”

Abbreviations: Bulk RNA analysis (RNA), peripheral blood analysis (Blood), multiplex immunohistochemistry (mIHC), Flow cytometry (Flow), pepinemab (pepi), nivolumab (nivo), ipilimumab (ipi).

Supplementary Table 4

| | surgery | | nivolumab | | pepinemab/nivolumab | | pepinemab/ipilimumab | | pepinemab/ipilimumab/nivolumab | |
|---|---|---|---|---|---|---|---|---|---|---|
| **Event** | **N=8** | | **N=8** | | **N=8** | | **N=8** | | **N=8** | |
| | number of patients (percent) | | | | | | | | | |
| | Any Grade | Grade 3 or 4 | Any Grade | Grade 3 or 4 | Any Grade | Grade 3 or 4 | Any Grade | Grade 3 or 4 | Any Grade | Grade 3 or 4 |
| Abdominal pain | 1 (12.5) | 0 | | | | | 1(12.5) | 0 | 1(12.5) | 0 |
| Acute Kidney injury | | | | | | | | | 1(12.5) | 0 |
| Adrenal insufficiency | | | | | | | 1(12.5) | 0 | | |
| Alanine aminotransferase increased | | | | | | | 2(25) | 0 | | |
| Anemia | | | | | 4(50) | 0 | 1(12.5) | 0 | | |
| Arthralgia | | | | | 2(25) | 2(25) | | | | |
| Aspartate aminotransferase increased | | | | | | | 2(25) | 0 | | |
| Blood bilirubin increased | | | | | 1(12.5) | 0 | 1(12.5) | 0 | | |
| Chills | 1(12.5) | 0 | 1(12.5) | 0 | 1(12.5) | 0 | 2(25) | 0 | 1(12.5) | 0 |
| Cough | | | 2(25) | 0 | | | | | | |
| Constipation | | | | | 1(12.5) | 0 | 2(25) | 0 | 1(12.5) | 0 |
| Diarrhea | | | 2(25) | 0 | 4(50) | 1(12.5) | 1(12.5) | 0 | 2(25) | 1(12.5) |
| Dizziness | | | | | | | 1(12.5) | 0 | | |
| Dysesthesia | | | | | 1(12.5) | 0 | | | | |
| Dyspnea | | | 1(12.5) | 0 | | | 1(12.5) | 0 | | |
| Fatigue | | | 1(12.5) | 0 | 1(12.5) | 0 | 5(62.5) | 0 | 4(50) | 0 |
| Fever | 1(12.5) | 0 | | | 2(25) | 0 | 1(12.5) | 0 | | |
| Flu like symptoms | | | | | | | 1(12.5) | 0 | | |
| General disorders and administration site conditions - Other, specify | | | 1 (12.5) | 0 | | | 2(25) | 0 | | |
| Generalized Muscle Weakness | | | | | 1(12.5) | 1 (12.5) | | | | |
| Headache | | | 1 (12.5) | 0 | 1(12.5) | 0 | 2(25) | 0 | 2(25) | 0 |
| Hemoglobin increased | | | | | | | 1(12.5) | 0 | | |
| Hepatitis viral | | | | | | | 1(12.5) | 0 | | |
| Hyperglycemia | | | | | 5(62.5) | 1(12.5) | 1(12.5) | 0 | | |
| Hypercalcemia | | | | | 1(12.5) | 0 | 1(12.5) | 0 | | |
| Hypokalemia | | | | | 1(12.5) | 0 | 1(12.5) | 0 | 1(12.5) | 1(12.5) |
| Hyponatremia | | | | | | | 1(12.5) | 0 | | |
| Hypothyroidism | | | | | 2(25) | 0 | 1(12.5) | 0 | 1(12.5) | 0 |
| Infusion site extravasation | | | | | | | 1(12.5) | 0 | | |
| Infusion related reaction | | | 1(12.5) | 0 | | | | | | |
| Injection site reaction | | | | | | | 1(12.5) | 0 | | |
| Insomnia | | | | | | | 2(25) | 0 | | |
| Laryngeal inflammation | | | | | | | 1(12.5) | 0 | | |
| Localized edema | | | 1(12.5) | 0 | 2(25) | 0 | 3(37.5) | 0 | | |
| Lymphedema | | | | | 4(50) | 0 | 1(12.5) | 0 | | |
| Myalgia | | | | | 1(12.5) | 0 | 1(12.5) | 0 | | |
| Nausea | | | | | 2(25) | 0 | 3(37.5) | 0 | 2(25) | 0 |
| Nasal congestion | | | | | | | | | 1(12.5) | 0 |
| Neck pain | | | | | | | 1(12.5) | 0 | | |
| Otitis externa | | | | | | | 1(12.5) | 0 | | |
| Pain | 1 (12.5) | 0 | 3(37.5) | 0 | 7(87.5) | 0 | 3(37.5) | 0 | 2(25) | 1(12.5) |
| Papulopustular rash | | | | | 1(12.5) | 0 | 2(25) | 0 | | |
| Paresthesia | 1 (12.5) | 0 | | | 1(12.5) | 0 | 1(12.5) | 0 | | |
| Platelet count decreased | | | | 0 | | | 2(25) | 0 | | |
| Postnasal drip | | | | | | | 2(25) | 0 | | |
| Pruritus | | | 1 (12.5) | 0 | 1(12.5) | 0 | 4(50) | 0 | 2(25) | 0 |
| Rash maculo-papular | | | | | | | 1(12.5) | 0 | | |
| Seroma | | | | | | | 1(12.5) | 0 | | |
| Skin Infection | | | | | | | | | 1(12.5) | 0 |
| Sore throat | | | | | | | 1(12.5) | 0 | | |
| Urinary Incontinence | | | | | 2(25) | 0 | | | | |
| Vertigo | | | | | | | 1(12.5) | 0 | | |
| Vomiting | | | | | | | 1(12.5) | 0 | 1(12.5) | 0 |
| Wound infection | | | | | | | | | 1(12.5) | 1(12.5) |
| White blood cell decreased | | | | | | | 1(12.5) | 0 | | |

## Grade specific toxicities summary.

Adverse events are reported by treatment arm as number of patients with percent in parenthesis.

Supplementary Table 5

| Treatment Arm | MDACC A | | MDACC B | | Total | |
|---|---|---|---|---|---|---|
| (Treatment Regimens) | ipilimumab + nivolumab | | nivolumab | | | |
| Subjects (N) | 7 | NA | 5 | NA | 12 | 100.0% |
| Age | | Mean (Range) | | Mean (Range) | | Mean (Range) |
| Median (Range) | 47 (31-76) | 47 (31-76) | 57 (41-68) | 57 (41-68) | 51 (31-76) | 51 (31-76) |
| Gender | | | | | | |
| Male | 7 | 100.0% | 3 | 60.0% | 10 | 83.3% |
| Female | 0 | 0.0% | 2 | 40.0% | 2 | 16.7% |
| Total | 7 | 100.0% | 5 | 100.0% | 12 | 100.0% |
| Race | | | | | | |
| Black or African American | 0 | 0.0% | 0 | 0.0% | 0 | 0.0% |
| White | 7 | 100.0% | 5 | 100.0% | 12 | 100.0% |
| Unknown or Not Reported | 0 | 0.0% | 0 | 0.0% | 0 | 0.0% |
| Total | 7 | 100.0% | 5 | 100.0% | 12 | 100.0% |
| ECOG PS | | | | | | |
| 0 | 6 | 85.7% | 2 | 40.0% | 8 | 66.7% |
| 1 | 1 | 14.3% | 3 | 60.0% | 4 | 33.3% |
| Total | 7 | 100.0% | 5 | 100.0% | 12 | 100.0% |
| Clinical Stage | | | | | | |
| Stage 3 (III) | 3 | 42.9% | 3 | 60.0% | 6 | 50.0% |
| Stage 4 (IV) | 4 | 57.1% | 2 | 40.0% | 6 | 50.0% |
| Total | 7 | 100.0% | 5 | 100.0% | 12 | 100.0% |

## Clinical and demographic characteristics of MDACC cohorts.

Baseline clinical and demographic characteristics of the MD Anderson Cancer Center (MDACC) biologic comparator cohort, stratified by treatment group. Variables include age, sex, race, ECOG performance status, and clinical stage. Data are presented as n (%) unless otherwise indicated.

**Emory University**
**Winship Cancer Institute**
**Protocol # Winship 4400-18**
**Version Date: 7/26/2022**
**Study Drugs: VX15/2503, ipilimumab, and nivolumab**
**IND# 141015**

# Pilot integrated biomarker study of VX15/2503 in combination with ipilimumab or nivolumab in patients with resectable metastatic melanoma


**Study PI**
Michael Lowe, MD, MA
Winship Cancer Institute of Emory University
1365 Clifton Road NE (2024)
Atlanta, GA 30322
Tel: 404-778-3738
mlowe3@emory.edu

Co-Investigators
Greg Lesinski, PhD
Brian Olson, PhD
Benjamin Stoff, MD
David Lawson, MD
Melinda Yushak, MD
Keith Delman, MD
Maria Russell, MD

Biostatistician

Subir Goyal Ph.D

Study Coordinators
Agnes Harutyunyan

OF CONTENTS

LIST OF ABBREVIATIONS ....................................................................................4
STUDY SYNOPSIS ....................................................................................6
1. INTRODUCTION ....................................................................................10
1.1 Melanoma ....................................................................................10
1.2 Semaphorin 4D Background ....................................................................................11
1.3 Study rationale ....................................................................................13
1.4 VX15/203 ....................................................................................14
1.5 Ipilimumab ....................................................................................17
1.6 Nivolumab ....................................................................................17
2. STUDY OBJECTIVES ....................................................................................18
2.1 Primary Objectives ....................................................................................18
2.2 Secondary Objectives ....................................................................................18
3. STUDY DESIGN ....................................................................................18
3.1 Phase I integrated biomarker study ....................................................................................18
3.2 Correlative biomarker collection ....................................................................................19
4. SELECTION OF STUDY POPULATION ....................................................................................23
4.1 Inclusion Criteria ....................................................................................23
4.2 Exclusion Criteria ....................................................................................24
5. TREATMENT OF SUBJECTS ....................................................................................25
5.1 Study Procedures ....................................................................................25
5.2 End of Treatment Visit ....................................................................................27
5.3 Survival Follow-up Assessment ....................................................................................28
5.4 Schedule of Events ....................................................................................30
5.5 Pre- and Post-Treatment Tumor Sample Collection ....................................................................................32
5.6 Dose Modifications and Stopping Treatment ....................................................................................32
5.7 Management of adverse events ....................................................................................33
5.8 Duration of Treatment ....................................................................................41
5.9 Subject Replacements ....................................................................................41
5.10 Removal of Subjects from Therapy or Assessment ....................................................................................41
5.11 Method of Assigning Subjects to Treatment Groups ....................................................................................42
6. TEST ARTICLE AND ADMINISTRATION ....................................................................................43
6.1 VX15/2503 ....................................................................................43
6.2 Nivolumab ....................................................................................44
6.3 Ipilimumab ....................................................................................45
6.4 Treatment Compliance ....................................................................................45
6.5 Drug Accountability ....................................................................................45
6.6 Contraindications, Precautions and Warnings ....................................................................................46
6.7 Concomitant Treatment ....................................................................................46
7. CORRELATIVE ASSESSMENTS ....................................................................................46
7.1 Pre-treatment tumor samples ....................................................................................46
7.2 Post-treatment tumor biopsy ....................................................................................46
7.3 Blood sample analysis ....................................................................................47

8. SAFETY ASSESSMENTS ........................................................................48
8.1 Clinical Safety Assessments........................................................................48
8.2 Laboratory Safety Assessments........................................................................48
8.3 Adverse Events/Experiences ........................................................................48
9. REPORTABLE EVENTS/INFORMATION ........................................................................51
9.1 AE/SAE Recording and Reporting........................................................................51
9.2 Reporting requirements of IND holder……………………………………51
9.3 Pregnancy…………………………………………………………………..52
10. DATA QUALITY ASSURANCE ........................................................................53
11. STATISTICAL CONSIDERATIONS ........................................................................53
11.1 Determination of Sample Size........................................................................53
11.2 Statistical and Analytical Plans ........................................................................53
11.3 Analysis Sets ........................................................................53
11.4 Subject Disposition and Baseline Characteristics…………………………...53
11.5 Safety analysis………………………………………………………………55
12. INVESTIGATOR REGULATORY OBLIGATIONS ........................................................................55
12.1 IRB/IEC Approval........................................................................55
12.2 Prestudy Documentation ........................................................................55
12.3 Informed consent……………………………………………………………55
12.4 Protocol Adherence ........................................................................55
12.5 Permission to Review Subjects' Source Records........................................................................55
12.6 Protocol Amendments ........................................................................56
12.7 Change in Investigator........................................................................56
12.8 Retention of Study Documents ........................................................................56
12.9 Date Safety and Monitoring……………………………………………..56
13. APPENDIX I: ECOG PERFORMANCE STATUS ........................................................................58
14. APPENDIX II: LAB MANUAL ........................................................................59
15. REFERENCES………………………………………………………………71

## LIST OF ABBREVIATIONS

| | |
|---|---|
| AE | adverse event/experience |
| ALT | alanine aminotransferase |
| aPTT | activated partial thromboplastin time |
| AST | aspartate aminotransferase |
| BP | blood pressure |
| CFR | Code of Federal Regulations |
| CRO | contract research organization |
| CT | computed tomography |
| CTCAE | Common Terminology Criteria for Adverse Events |
| CYP | Cytochrome P450 |
| D5W | dextrose 5% in water |
| DEHP | di(2-ethylhexyl) phthalate |
| DCR | disease control rate |
| dL | Deciliter |
| DPD | Dihydropyrimidine dehydrogenase |
| ECG | Electrocardiogram |
| ECHO | Echocardiogram |
| ECOG | Eastern Cooperative Oncology Group |
| FDA | Food and Drug Administration |
| FFPE | Formalin-fixed paraffin-embedded |
| GI | Gastrointestinal |
| HIPAA | Health Insurance Portability Act |
| HIV | human immunodeficiency virus |
| HR | Heart rate |
| ICH | International Conference on Harmonisation |
| IEC | Independent Ethics Committee |
| INR | International normalized ratio |
| IRB | Institutional Review Board |
| IV | Intravenous |
| LC-MS/MS | liquid chromatography-mass spectrometry / mass spectrometry |
| LDH | lactate dehydrogenase |
| LMW | Low molecular weight |
| MDSC | Myeloid-derived suppressive cells |
| MedDRA | Medical Dictionary for Regulatory Activities |
| mg | Milligram |
| NCI | National Cancer Institute |
| PFS | Progression-free survival |

| | |
|---|---|
| PICCS | Peripherally-inserted central catheters |
| PK | Pharmacokinetic |
| PLXNBI | Plexin-B1 |
| PLXNB2 | Plexin-B2 |
| PT | Prothrombin time |
| PR | partial response |
| PVC | polyvinyl chloride |
| ORR | objective response rate |
| OS | overall survival |
| QT | cardiac interval from start of Q wave to end of T wave |
| RDC | remote data capture |
| RR | respiration rate |
| SAE | serious adverse event |
| SEMAD4 | Semaphorin-4 |
| SD | stable disease |
| TME | tumor microenviroment |
| ULN | upper limit of normal |
| VADs | Vascular access devices |
| WHO | World Health Organization |

## STUDY SYNOPSIS

| |
|---|
| **Title of Study**: Pilot integrated biomarker study of VX15/2503 in combination with ipilimumab or nivolumab in patients with resectable metastatic melanoma |
| **Study Center**: Emory University |
| **Study Phase**: Pilot integrated biomarker study |
| **Objectives**:<br>**Primary Objective:**<br>To evaluate the effect of VX15/2503 in combination with immune checkpoint inhibitors on T cell infiltration into the tumor microenvironment in involved and uninvolved lymph nodes and peripheral blood.<br><br>**Secondary Objectives:**<br>To evaluate the effect of VX15/2503 in combination with immune checkpoint inhibitors on the immune profile of involved and uninvolved lymph nodes and peripheral blood.<br><br>To assess safety profile and tolerability of the combination of VX15/2503 and immune checkpoint inhibitors in patients with resectable metastatic melanoma.<br><br>To document pathologic response rates of the combination of VX15/2503 and immune checkpoint inhibitors in patients with resectable melanoma.<br><br>To compare pathologic response to radiographic response using RECIST criteria in patients receiving single agent nivolumab and combination VX15/2503 and immune checkpoint inhibitors in patients with resectable melanoma. |
| **Trial Design/Methodology**: A neoadjuvant trial of VX15/2503 in combination with systemic immunotherapy (ipilimumab or nivolumab) in patients with resectable metastatic melanoma. Total number will be a maximum of 36 patients with melanoma. |

| Disease | Cohort | Treatment | Dose Frequency |
|---|---|---|---|
| Melanoma | A | VX15/2503 (15 mg/kg) | Q3W (days 1, 21) |
| | | Nivolumab (360 mg) | Q3W (days 1, 21) |
| Melanoma | B | VX15/2503 (15 mg/kg) | Q3W (days 1, 21) |
| | | Ipilimumab (3 mg/kg) | Q3W (days 1, 21) |
| Melanoma | C | VX15/2503 (15 mg/kg) | Q3W (days 1, 21) |
| | | Nivolumab (3 mg/kg) | Q3W (days 1, 21) |
| | | Ipilimumab (1 mg/kg) | Q3W (days 1, 21) |
| Melanoma | D | Nivolumab (360 mg) | Q3W (days 1, 21) |
| Melanoma | E | No treatment (SOC) | n/a |

All patients (except cohort E) will undergo surgery between day 35 and 49. Patients in cohort E will undergo surgery within 30 days of obtaining consent.

Eight patients will be enrolled into cohorts A, B, C and D; six patients will be enrolled into cohort E. Patients will be permitted to choose between one of two groups: no treatment (E) or treatment (A, B, C, or D) until the no treatment group is filled. Each treatment cohort will be enrolled to completion sequentially. In the event a patient experiences unacceptable toxicity unrelated to the known toxicity of immunotherapy (Grade 4 non-hematologic or persistent Grade 3 hematologic toxicity), treatment for that arm will be paused (pending further evaluation). Accrual to the trial will also be paused (pending further evaluation) if 2 patients in one treatment arm experience adverse events that delay their surgery (beyond day 49) or ≥ 25% of patients across treatment arms (after enrollment of at least 10 patients) experience adverse events that delay their surgery (beyond day 49). Continued accrual is permitted only if it is determined that the adverse events leading to delay in surgery were not related to treatment or that the delay caused by the event, regardless of attribution, did not result in previously resectable sites of disease to become unresectable.

Following surgery, patients in all cohorts will receive standard of care nivolumab adjuvant therapy for one year at the discretion of the treating physicians.

**Number of Subjects**:

45 patients

**Duration of treatment**:

Treatment will be given on days 1 and 21; surgery will take place between day 35 and 49.

**Main criteria for inclusion**:

*Patient-specific criteria*

- Informed consent
- Age > 18 years
- ECOG performance status of 0 or 1
- Adequate bone marrow function (leukocyte count ≥2,000/mm$^3$; platelet count ≥100,000/mm$^3$; and hemoglobin count ≥9.0gm/dL)
- Adequate renal function (creatinine clearance ≥ 50 mL/min; or serum creatinine level ≤1.5 mg/dL)
- Adequate hepatic function (bilirubin ≤ 1.5 x upper normal limit; AST, ALT, and alkaline phosphatase ≤2.5 x upper normal limit)
- INR ≤1.5. (Anticoagulation is allowed if target INR ≤ 1.5 on a stable dose of warfarin). Patient receiving LMW heparin on stable therapeutic dose for more than 2 weeks or with factor Xa level <1.1U/mL are allowed on the trial.

*Disease-specific criteria:*

- Biopsy-accessible surgically resectable stage IIIB/IIIC/IIID melanoma (AJCC 8th edition)
- No prior immunotherapy for melanoma. Prior interferon will be allowed as long as it was greater than 1 year prior to consent.

| |
|---|
| **Main criteria for exclusion**:<br>• Pregnancy<br>• Unequivocal visceral or brain metastatic disease on imaging<br>• Clinically significant comorbid conditions such as cardiovascular disease or significant peripheral vascular (e.g., uncontrolled hypertension, myocardial infarction, unstable angina) within 6 months of study entry, serious cardiac arrhythmia requiring medication, and uncontrolled infection.<br>• Pre-existing medical conditions that prevent patients from undergoing planned surgery.<br>• Unresectable disease at presentation |
| **Investigational product and administration**: VX15/2503 is formulated for intravenous (IV) administration as a sterile solution of 20 mg/mL antibody in 10 mL vials containing 10 mL at a concentration of 20 mg/mL; each vial is intended for single use. Eligible subjects will receive VX15/2503 as an IV infusion over 60 minutes. The amount of VX15/2503 administered is 15 mg/kg. |
| **Control therapy, dose and administration**: The inclusion of a cohort of patients who proceed directly to surgery without study drugs will serve as a control for comparison to those receiving the lead-in immunotherapy treatment. Patients receiving nivolumab alone will serve as a control group to assess the additive effects of VX15/2503 to immunotherapy combinations. |
| **Efficacy evaluation/Endpoints**:<br>**Primary Endpoint**: To determine if VX15/2503 in combination with immune checkpoint inhibitors modulates the phenotypic properties within the tumor microenvironment in a manner that enhances T effector cell infiltration.<br><br>**Secondary Endpoints**: To evaluate the effect of VX15/2503 in combination with immune checkpoint inhibitors on the immune profile of involved and uninvolved lymph nodes and peripheral blood. To determine if the combination of VX15/2503 and immune checkpoint inhibitors is safe with no added toxicity when administered for a brief, 6-week period prior to surgical resection. To determine pathologic response rate of immune checkpoint inhibitors with or without VX15/2503. To compare pathologic response to radiographic response using RECIST 1.1. |
| **Safety evaluation/Endpoints**:<br>NCI Common Toxicity Criteria Version 5.0 |
| **Rationale for number of subjects**: 8 patients in each of the combination treatment arms are to ensure that there can be meaningful biomarker comparison between the treatment arms: 1) VX15/2503 and nivolumab, 2) VX15/2503 and ipilimumab, and 3) VX15/2503 with nivolumab and ipilimumab. Given most preclinical data with VX15/2503 is in combination with checkpoint inhibitors, 8 patients will be enrolled on the nivolumab alone arm and 6 patients will be enrolled in the no treatment control. |
| **Statistical methods**: This study is a pilot to evaluate tissue biomarkers in the setting of VX15/2503 with checkpoint inhibitors. |

| |
|---|
| The two-sample t-test will be used to compare the change in expression of biomarker change after treatment between each experimental group (Cohort A, B, C, and D) and the control group (cohort E), respectively. A Bonferroni correction will be used to control type I error at 5% across 4 comparisons. Assuming a CV of 50%, n=8 patients in experiment group and 8 patients in nivolumab and 6 patients in no treatment control groups can achieve at least 80% power to detect a 3-fold difference in change of expression by treatment between the pairwise groups ($\alpha$=0.0125, 2-sided). A mixed model with random effect for intercept and group will be used to compare the overall change in expression of biomarker during the whole experiment among the different groups. If there is a significant difference overall, pairwise comparisons of overall change in expression of biomarker will be further conducted for each experimental group compared with the control group, respectively. ANOVA will be conducted to compare the change in biomarkers and other outcomes at the treatment endpoint from baseline between the different groups. Pairwise group differences will be further tested by using Tukey's method when the overall difference is significant at a level of 0.05. |
| **Translational Research**: Patient tumor biopsies prior to any therapy (diagnostic biopsy, if available) and at surgical resection will be processed to analyze for tumor biomarkers. Tissue will be analyzed for biomarkers related to activated tumor stroma, immune infiltrates, and targets related to drug activity (SEMA4D, plexin-B1) via immunohistochemical or immunofluorescence analysis or other assays as appropriate.<br><br>At baseline (day 1), day 21, prior to surgery (day 35 – 49), and at progression or at completion of adjuvant therapy, we plan to collect blood for correlative assays for all patients enrolled on the trial. Blood will be evaluated for: circulating percentage of immune suppressor subsets, phenotypic shifts function in T lymphocyte subsets. |

# 1. INTRODUCTION AND STUDY RATIONALE

## 1.1 Melanoma

Melanoma is an aggressive cutaneous malignancy with an estimated 87,000 new diagnoses and 9,700 deaths in 2017.[1] Prognosis and treatment for melanoma vary widely and depend on the stage of disease, with early stage patients often being cured by surgery alone. Historically survival rates for patients with regional or distant metastases are much worse, with 5-year survival rates less than 60% for patients with regional disease and less than 20% for patients with metastatic disease. This dismal survival has been attributed to the fact that melanoma is resistant to cytotoxic therapies, which formed the impetus to investigate the use of immunotherapies in melanoma.

The first breakthrough for immune therapy in melanoma came with the introduction of ipilimumab, a monoclonal antibody to CTLA-4, a member of the costimulatory B7 family of receptor signals that affects the development and regulation of T cell adaptive response. It was shown to improve overall survival in patients previously treated for unresectable stage III and IV melanoma compared to a gp100 peptide vaccine.[2] While these results provided significant impact on survival from melanoma, toxicities associated with this antibody were relatively significant and response rates were lower than anticipated.

The next class of immune therapy tested in unresectable metastatic melanoma targeted the PD-1 molecule, an inhibitory receptor expressed on activated T cells. Early studies of pembrolizumab (Merck) and nivolumab (BMS-936558) showed significant improvement in survival in ipilimumab-refractory patients compared to chemotherapy.[3,4] PD-1 inhibition with pembrolizumab was later shown to be superior to ipilimumab in treatment-naïve patients.[5] Recent data has shown a significant improvement in survival when PD-1 inhibition with nivolumab is combined with ipilimumab compared to patients treated with ipilimumab alone.[6]

Having shown efficacy in the unresectable metastatic setting, these agents were then tested in the adjuvant setting following resection of macrometastatic nodal or oligometastatic systemic disease. When compared to placebo, ipilimumab improved overall and distant metastasis-free survival for stage III patients undergoing curative-intent surgery.[7] This led to FDA approval of adjuvant ipilimumab in 2015, the first therapy approved for this patient population since the approval of interferon-alpha2b in 1996. Given the success of PD-1 inhibition in the metastatic setting, nivolumab was then compared to ipilimumab in the adjuvant setting. For patients undergoing surgery for stage IIIB, IIIC, or IV melanoma, adjuvant nivolumab significantly prolonged recurrence-free survival compared to ipilimumab, and nivolumab was recently FDA-approved for use in the adjuvant setting.[8]

Despite the resounding improvement in survival with these novel immune therapies, response rates are marginal. The response rate to single-agent ipilimumab in unresectable metastatic melanoma is approximately 20%, while the response rate to PD-1 inhibitors (nivolumab or pembrolizumab) is 40%. When combined, the response rates improve to 60% with over 60% overall survival at 2 years in Stage IV disease. A number of barriers may account for poor response or resistance following initiation of immunotherapy. These include expansion of immunosuppressive cell populations and cytokines, resistance of tumor cells to cytotoxic lymphocyte-mediated lysis, and inadequate T cell trafficking to tumors. Thus, therapeutic strategies to modulate the tumor microenvironment and to modulate the immune system may enhance the efficacy of immunotherapy.

### 1.2 Semaphorin 4D Background

Semaphorins are a family of soluble and membrane-bound proteins that were originally defined as axonal-guidance factors. Semaphorins have also been shown to have important functions in other physiological processes such as vascular growth, tumor progression, and immune cell regulation.[9]

Semaphorin 4D (SEMA4D) is a 150 kDa transmembrane protein that is expressed abundantly on the surface of resting T cells, and to a lesser extent on resting B cells, monocytes, and antigen-presenting cells, such as dendritic cells. Cellular activation upregulates surface expression of SEMA4D on B cells and dendritic cells, as well as the generation of soluble SEMA4D. SEMA4D has also been reported to be present on tumors, oligodendrocytes and the surface of platelets. SEMA4D surface expression increases with platelet activation and is followed by a gradual shedding of the extracellular domain.[10] Normal human serum contains between 5 and 20 ng/mL of SEMA4D.

Three cellular receptors have been identified for SEMA4D: Plexin-B1 (PLXNB1), Plexin-B2 (PLXNB2), and CD72. PLXNB1, the highest-affinity SEMA4D receptor (KD = 1 nM) is expressed on antigen presenting cells, as well as in non-lymphoid tissues, including some tumor cells, endothelial cells, and neural cells.[11,12] SEMA4D engagement with PLXNB1 has been shown to induce activation and migration of endothelial cells, and to regulate migration of tumor cells and leukocytes.[13,14,15] Association of PLXNB1 with oncogenic tyrosine kinase signaling partners, such as cMET and ERBB2, can promote invasive tumor growth.[12,13,16,17] PLXNB2, the best characterized ligand of which is SEMA4C, also binds SEMA4D with approximately 5 nM affinity. A recent report indicates that PLXNB2 is expressed on keratinocytes and can activate SEMA4D-positive γδ T cells to contribute to epithelial wound repair.[18] CD72 is a relatively low-affinity (KD = 300 nM) SEMA4D receptor, and is found in lymphoid tissues where it is expressed primarily on B cells, APCs and platelets.[19]

Several lines of preclinical evidence support an immunomodulatory function of SEMA4D, in which SEMA4D retards the recruitment of effector leukocytes into a tumor, which in turn allows for unchecked tumor growth (Investigator Brochure for additional details)[20,21]:

- In preclinical models using immunohistochemical staining of murine syngeneic tumors, a striking gradient of SEMA4D expression was revealed at the margin where the tumor interfaces with the stroma. (Figure 1) Treatment with an anti-SEMA4D antibody disperses SEMA4D and eliminates the SEMA4D barrier. (Figure 2)

- Antibody blockade of SEMA4D regulates the balance and activity of inflammatory and tolerance-inducing cells and cytokines. Specifically, there is an increase in the presence of infiltrating cytotoxic T cells, dendritic cells and macrophages within the tumor. (Figure 3) Other infiltrating T cells secrete cytokines to produce a Th1 environment within the tumor. Furthermore, immunosuppressive cells, including T-regulatory cells and myeloid derived suppressor cells (MDSC), and associated chemokines are reduced. These changes are associated with decreased tumor growth and the promotion of durable immune-mediated tumor rejection in syngeneic murine tumor models.

- In preclinical models, the combination of anti-SEMA4D antibody with antibodies targeting CTLA-4 and PD-1 significantly improves complete tumor rejection and survival, compared to the activity of single agent antibodies.

**Figure 1 Immunohistochemical Staining of Murine Syngeneic Tumors**

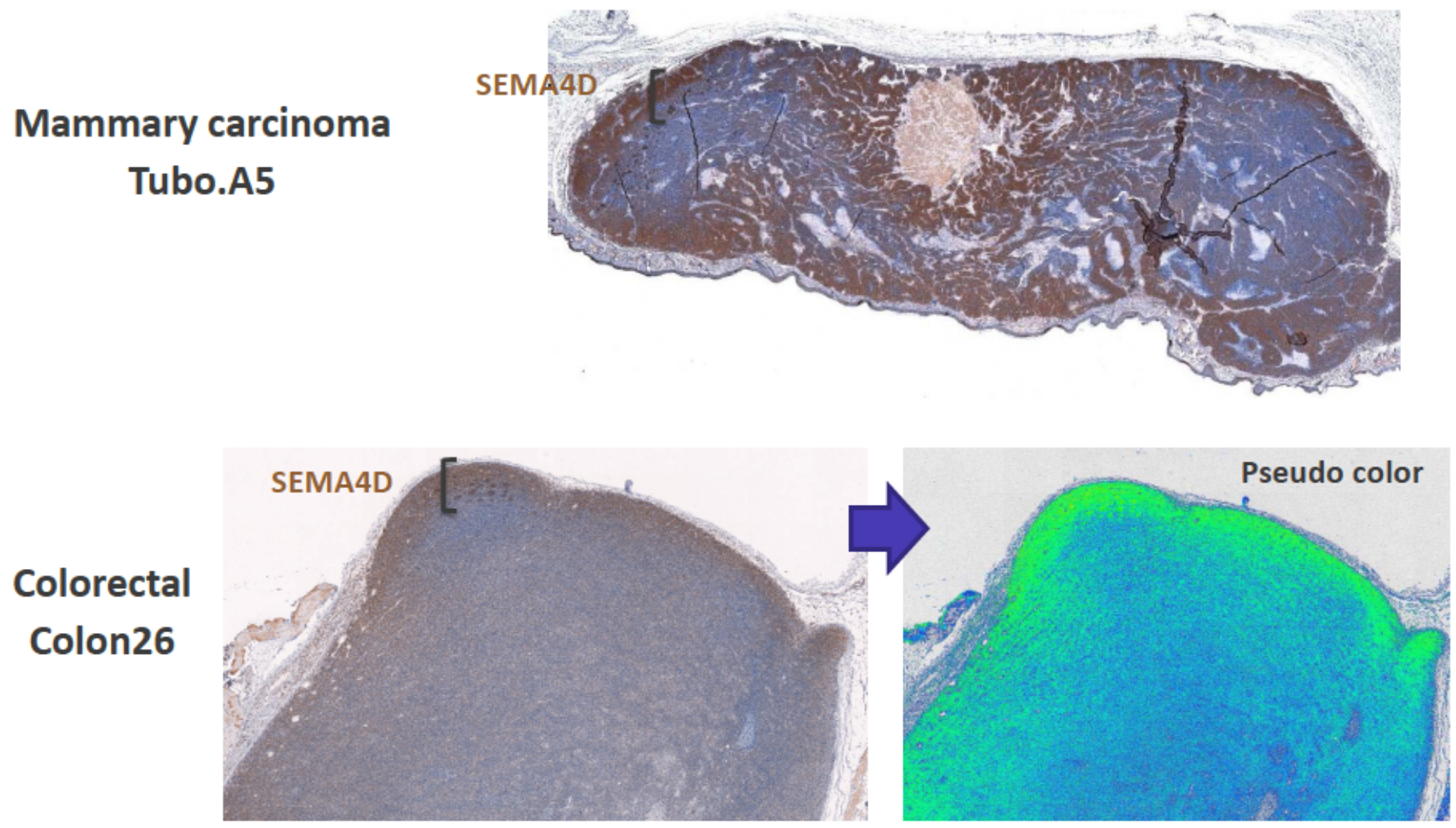


**Figure 1 Legend:** Low magnification images show strong SEMA4D staining at the invasive tumor margins (brackets) of murine colorectal and breast tumors. [18]

**Figure 2 Effect of Anti SEMA4D on the Presence of Infiltrating Immune Cells**

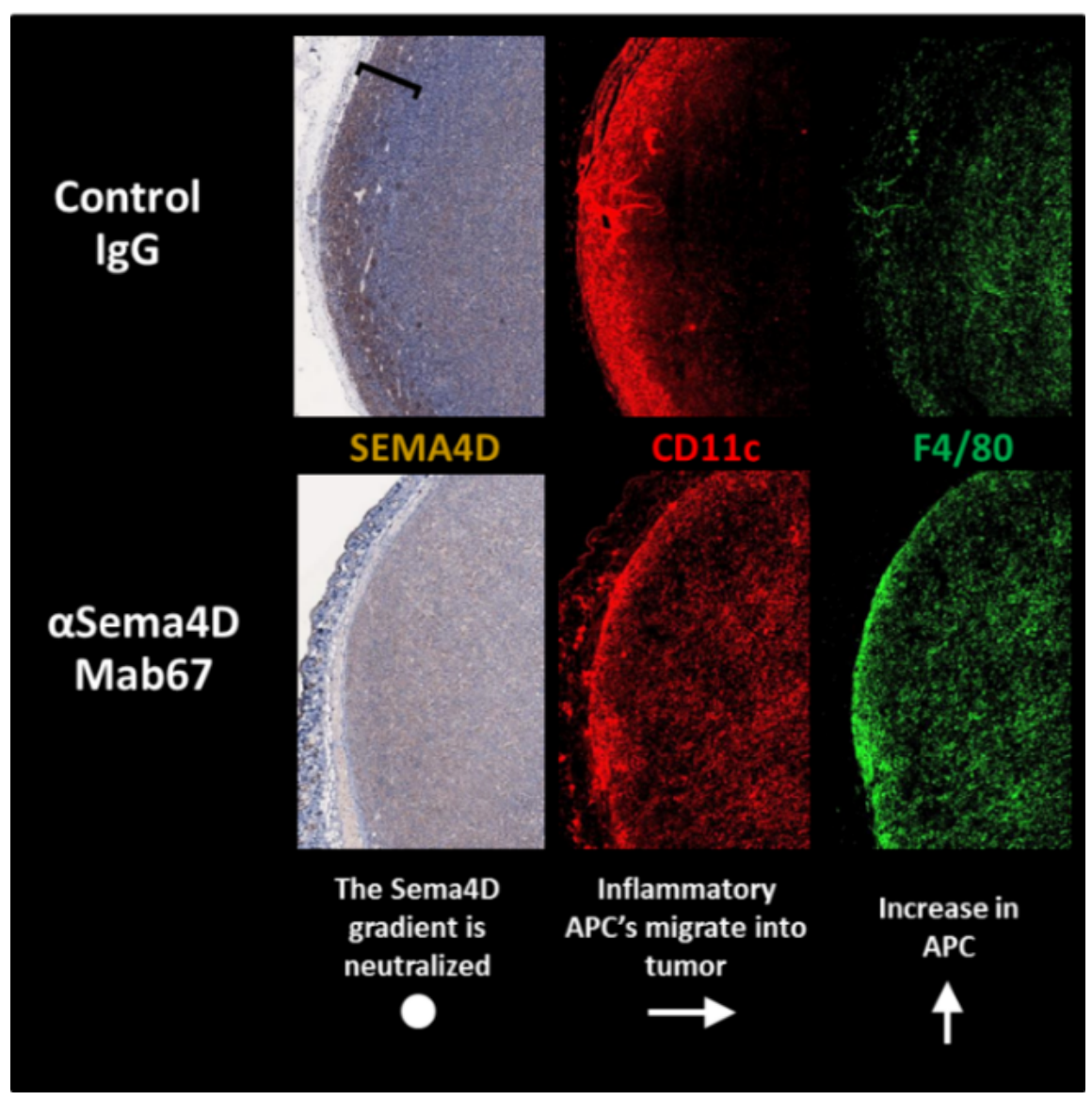


**Figure 2 Legend:** This immunohistological specimen from a Colon26 tumor shows a peri-tumoral SEMA4D zone in the control specimen on the top. Treatment with an anti-SEMA4D antibody disperses SEMA4D and eliminates the SEMA4D barrier as shown in the specimen on the bottom. Treatment also enhances penetration of activated antigen presenting cells, including CD11c+ dendritic cells and F480+ macrophages, into the tumor.[18]

**Figure 3 Cytotoxic T cell (CD8+) Infiltration Following Anti-SEMA4D Antibody**

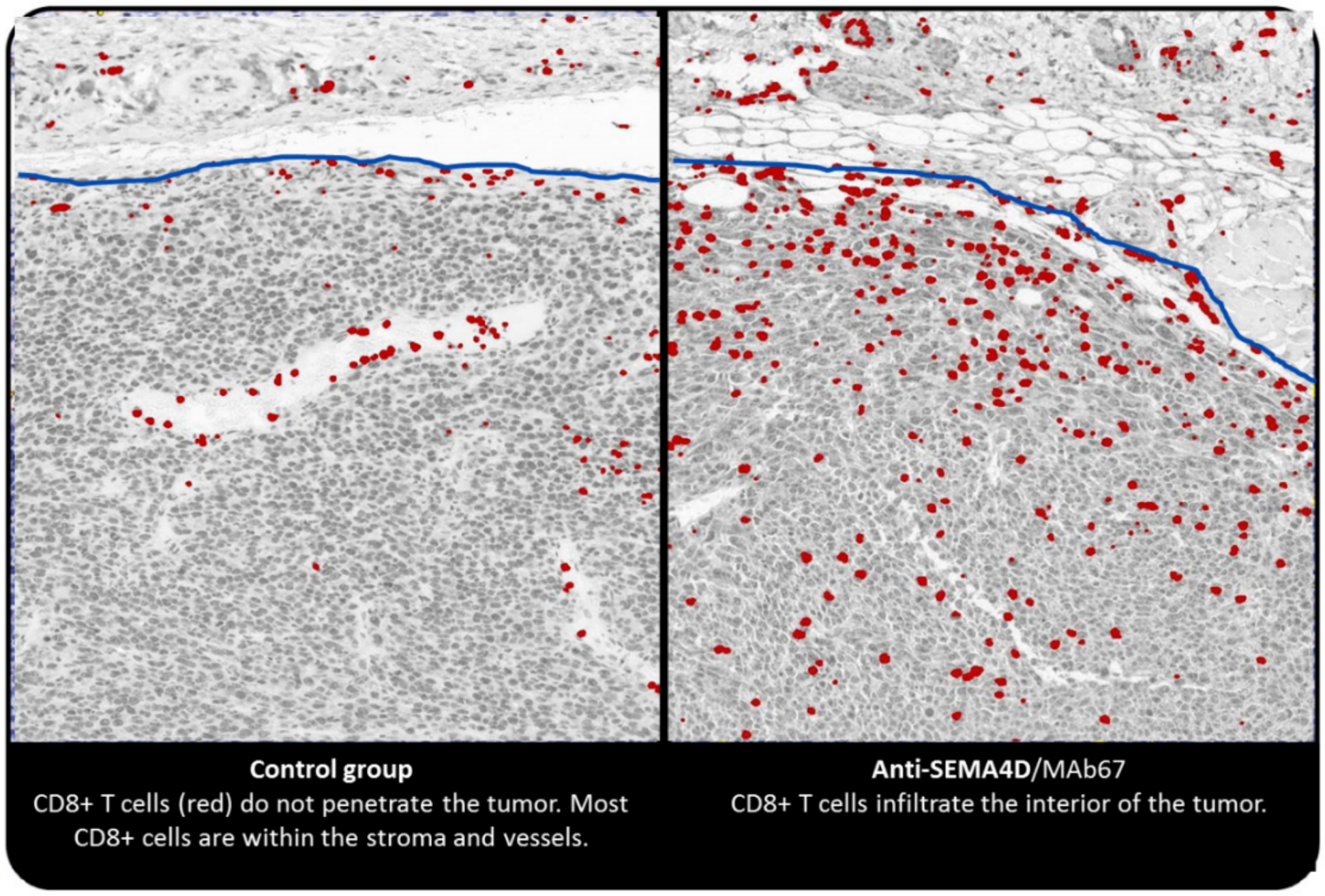


**Figure 3 Legend:** This murine immunohistological specimen shows staining for cytotoxic T cells (red) prior to treatment with an anti-SEMA4D antibody (negative control on the left) and following treatment with anti-SEMA4D antibody on the right. The red staining indicates that treatment with the anti-SEMA4D antibody increases the infiltration of CD8+ T cells into the tumor. The blue line indicates the tumor invasive margin.[41]

In addition, immunohistochemical analysis of SEMA4D and PLXNB1 expression by several human tumor types revealed that SEMA4D and PLXNB1 are broadly expressed in human tumors.[18] Furthermore, elevated expression of SEMA4D and its receptor PLXNB1 correlates with invasive disease and poor prognosis in human cancer, including breast, ovarian, pancreatic, and soft-tissue sarcomas. [18] (Investigator brochure).

### 1.3 Study Rationale

Pre-clinical studies with murine colon cancer models have shown that tumors have high expression of SEMA4D at tumor invasive margins and, when treated with an antibody to SEMA4D, neutralization of the SEMA4D gradient at the invasive border enhances recruitment of monocytes and lymphocytes into the tumor. In addition, when VX15/2503, an anti-SEMA4D antibody, was combined with either anti-CTLA4 or anti-PDL-1 treatments, there was an additive effect in tumor volume reduction. **Together, the available data suggest that SEMA4D blockade will modulate the contexture of immune cells within the tumor microenvironment, thereby enhancing the efficacy of immune checkpoint inhibitors.**

The standard of care for resectable melanoma is to undergo surgery followed by adjuvant immune therapy. The outcome of patients undergoing resection for metastatic melanoma is marginal, with median 5-year survival of roughly 50%. **New therapies are needed in this setting and the preoperative period is an ideal time to test the effect of new treatments in this population.** Testing novel agents in the pre-operative period allows us to obtain tissue at time of surgery in

order to better understand the mechanism of action and resistance to our novel treatments **without impacting the standard of care for these patients**.

We propose a 6-week neoadjuvant window of opportunity trial of VX15/2503 in combination with immunotherapy (ipilimumab or nivolumab) in patients who are undergoing curative-intent surgery. There will be 5 treatment arms:

A) VX15/2503 and nivolumab (n=8)
B) VX15/2503 and ipilimumab (n=8)
C) VX15/2503, nivolumab and ipilimumab (n=8)
D) nivolumab alone (n=6)
E) No treatment/direct to surgery (n=6)

We ***hypothesize*** that neutralization of SEMA4D with VX15/2503 will allow for greater access of immune effector cells into the tumor microenvironment, and thereby improve the efficacy of immunotherapy as seen in pre-clinical models of disease. We expect to see increase in immune effector cells within the tumor and at the tumor margin with VX15/2503 treatment as compared to no treatment or to treatment with PD-1 inhibitor alone. This clinical trial will fill an important gap in our understanding of how blockade of SEMA4D by VX15/2503 can shape the stroma, and the impact of targeting it to augment immunotherapy. **This study will provide data to justify a larger trial evaluating the therapeutic benefit of such an intervention in resectable regional melanoma metastases.**

### 1.4 VX15/2503

VX15/2503 is a humanized IgG4 monoclonal antibody that binds with an approximately 1 to 5 nM affinity specifically to SEMA4D (CD100).[22]

**Preclinical Data Summary**

A program of preclinical investigation was conducted using in vitro and in vivo methods to evaluate the pharmacology, toxicology, and pharmacokinetics of VX15/2503 and identify a dose at which to initiate clinical testing.
A summary of preclinical data is presented below. The VX15/2503 Investigator's Brochure contains additional details regarding the preclinical evaluation program.

**In vitro studies**

In vitro studies were performed to evaluate the ability of VX15/2503 to interfere with the binding of SEMA4D to its receptor PLXNB1. These studies confirmed that VX15/2503 blocks the binding of SEMA4D to the PLXNB1 receptor and blocks the functional activity of SEMA4D in a variety of cell-based assays. The parental antibody of VX15/2503, murine MAb 67-2, increased survival in various murine oncology models. Tissue cross reactivity studies performed using a panel of fresh-frozen human, cynomolgus macaque and rat tissues showed concordance of the antibody reactivity profile across species. Specific positive reactivity was seen with individual lymphoid cells in human brain (cortex), lung, and spleen. A more comprehensive immunohistology study using a large panel of fresh frozen human tissues demonstrated similar specific reactivity with lymphoid cells in various tissues.

### Absorption, Metabolism and Excretion Studies

VX15/2503 is administered intravenously, and therefore absorption is 100%. No distribution, metabolism, or excretion studies have been performed with VX15/2503 in humans. However, pharmacokinetics and pharmacodynamics analyses have been performed on plasma concentration vs. time data collected in CSJL mice, Sprague Dawley rats, and Cynomolgus macaques. In general, the pharmacokinetics of VX15/2503 are similar across species. VX15/2503 half-life increases with dose and, as expected, saturation of T cell-associated SEMA4D is also dose dependent. SEMA4D saturation declines once the serum concentration of VX15/2503 falls below approximately 5 to 10 mcg/mL.

### Pharmacokinetics

No studies designed specifically to examine the pharmacokinetics of VX15/2503 were conducted in mice; however, estimates of various pharmacokinetics parameters were made based on serum level data collected during various murine research experiments conducted with related murine anti-SEMA4D antibodies (i.e., MAb 67-2, the murine homolog of VX15/2503; and MAb 76 1, a mouse anti-SEMA4D antibody recognizing a different epitope than MAb 67-2).

More formal pharmacokinetics analyses were conducted with Sprague Dawley rats in a single dose non-GLP study and with Cynomolgus macaques in a GLP single dose study. Pharmacokinetics data were also collected from repeat dose GLP studies using these same species.[23]

In general, longer half-lives were estimated for higher doses and the apparent volume of distribution approximated the estimated serum volume for each species. The half-life at 100 mg/kg was about 10 days; that at 10 mg/kg was about 4 days. AUC did not increase linearly with dose but rather increased more rapidly than dose.

### Drug-Drug Interactions

No drug-drug interaction studies have been conducted with VX15/2503.

### Toxicology Studies

Two single dose, two one-month, and two six month repeat dose toxicology studies were performed with VX15/2503 at doses from 0.01 mg/kg to 200 mg/kg, using Sprague Dawley rats and cynomolgus macaques.[24]

No significant toxicological findings were observed; however, a 33 to 80% reduction in NK cell levels was observed in all treated rats. No similar effect was noted for other lymphocyte subsets, and no similar NK cell reduction was noted in the Cynomolgus macaque or in humans. In each of the chronic toxicology studies, the highest dose administered (100 mg/kg or 200 mg/kg) was determined to be the no observed adverse effect level (NOAEL). Finally, a host-resistance study using rats challenged with flu virus after five weekly doses of VX15/2503 at dose levels including 200 mg/kg showed that antibody treatment was not immunosuppressive.

### Other Toxicology-Related Findings

**Platelet Function:** Platelets express SEMA4D on their surface and demonstrate a mild defect in collagen-induced platelet aggregation in platelets from SEMA4D knock-out mice.[8,25] VX15/2503 binds to platelets but does not activate them on its own and does not inhibit collagen-induced platelet aggregation.

**Peripheral Blood Mononuclear Cell Proliferation:** VX15/2503 antibody does not induce proliferation or cytokine release of human peripheral blood mononuclear cells.

**Host Resistance:** Host resistance to influenza infection was not diminished by pretreatment with VX15/2503 in a rat model of viral resistance, compared to a positive control group pretreated with dexamethasone.[24]

No carcinogenicity, reproductive or genotoxicity/mutagenicity studies have been conducted with VX15/2503.

### Clinical Data

VX15/2503 is currently under clinical investigation in various cancer indications and Huntington's disease.

#### 1.4.1.1 Study VX15/2503-01

VX15/2503-01 was a phase 1, non-randomized, open-label, dose-escalation safety study in adult subjects with advanced resistant or refractory solid tumors.[26] The starting dose was 0.3 mg/kg, with subsequent dosing levels of 1, 3, 6, 9, 15 and 20mg/kg. Dose escalation proceeded on a standard 3 + 3 dose escalation scheme. Enrollment for this study began in January 2011 and last patient last visit (LPLV) occurred in February 2014. Total VX15/2503 exposure in this phase 1 oncology study included 42 subjects who received a total of 460 weekly doses with no reported treatment-related SAEs.

### Safety Findings

Forty-two subjects were enrolled into seven sequential cohorts and an expansion cohort (20 mg/kg). VX15/2503 was well tolerated. Treatment-related adverse events were primarily grade 1 or 2 and included nausea (14.3%) and fatigue (11.9%); arthralgia, decreased appetite, infusion-related reaction, and pyrexia were each 7.3%. One pancreatic cancer subject (15 mg/kg) experienced a Grade 3 dose-limiting toxicity; elevated g-glutamyl transferase. Complete cSEMA4D saturation was generally observed at serum antibody concentrations 0.3 mg/mL, resulting in decreased cSEMA4D expression. Soluble SEMA4D levels increased with dose and infusion number. Neutralizing anti-VX15/2503 antibodies led to treatment discontinuation for 1 subject. VX15/2503 $C_{max}$ and AUC generally increased with dose and dose number. One subject (20 mg/kg) experienced a partial response, 19 subjects (45.2%) exhibited SD for 8 weeks, and 8 (19%) had SD for 16 weeks. Subjects with elevated B/T lymphocytes exhibited longer progression-free survival. VX15/2503 was well tolerated and produced expected PD effects. The correlation between immune cell levels at baseline and progression-free survival is consistent with an immune-mediated mechanism of action. Future investigations will be in combination with immunomodulatory agents.

### Pharmacokinetics

Table 1 below provides an integrated summary comparing PK parameters measured for similar doses in the two VX15/2503 clinical trials performed to date. PK parameters could only be calculated for the 0.3 mg/kg and 1.0 mg/kg doses due to the serum sampling times available. Half-life appears to increase with dose and clearance appears to decrease with dose. AUC values suggest nonlinear kinetics for systemic exposure, and $t_{1/2}$, CL, Vz, and Vss support this interpretation. The nonlinear increase in $t_{1/2}$ with dose and other PK parameters may be related to target-mediated drug disposition, which is commonly observed with monoclonal antibodies (MAbs). The values for VSS suggest the antibody is distributed within the circulatory system.

Table 1 Integrated Summary of PK Parameters for VX15/2503 Clinical Trials

| Parameter | MS 1 mg/kg (N=8) | Onc 1 mg/kg (N=1-4) | MS 3 mg/kg (N=8) | MS 6 mg/kg (N=8) | MS10 mg/kg (N=8) | MS20 mg/kg (N=8) |
|---|---|---|---|---|---|---|
| Cmax (mcg/mL) | | | | | | |
| Mean | **19.29** | **13.88** | **45.44** | **97.00** | **168.9** | **274.28** |
| SD | 8.140 | 4.820 | 4.553 | 15.46 | 39.72 | **65.227** |
| $t_{1/2}$ (days) | | | | | | |
| Mean | **3.86** | **3.98** | **5.55** | **10.23** | **15.40** | **20.39** |
| SD | 1.65 | 0.77 | 1.70 | 3.08 | 5.621 | 5.00 |
| $V_{ss}$ (L) | | | | | | |
| Mean | **5.36** | **13.4** | **6.15** | **8.89** | **10.42** | **12.16** |
| SD | 1.12 | NC[1] | 1.13 | 2.87 | 3.00 | 1.89 |
| Cl (L/hr) | | | | | | |
| Mean | **0.0530** | **0.11** | **0.0404** | **0.0358** | **0.0251** | **0.0201** |
| SD | 0.0126 | NC | 0.0121 | 0.0141 | 0.0072 | 0.0053 |
| $AUC_{0\text{-infinity}}$ | | | | | | |
| Mean | **1,490** | **870.8** | **5,687** | **13,903** | **33,765** | **87,178** |
| SD | 468.1 | NC | 1,115 | 2,986 | 8,720 | 32,628 |

[1] NC = not calculated
MS=VX15/2503-N-101
Onc=VX15/2503-01

Please refer to the Investigator's Brochure for additional information.

### 1.5 Ipilimumab

CTLA-4 is a member of the costimulatory B7 family of receptor signals and affects the development and regulation of T cell adaptive response.[27] CTLA-4 is expressed on T-regulatory cells, CD4+ and CD8+ T cells. CTLA-4 suppresses the immune system by downregulating effector T cells and modulating T regulatory cells, and this has been especially described in the tumor microenvironment of PDAC. CTLA-4 blockade enhances the immune response.

Ipilimumab is a humanized anti-monoclonal IgG1 antibody to CTLA-4 that is approved by the Food and Drug Administration (FDA) for the treatment of metastatic, unresectable melanoma at the dose of 3mg/kg IV over 90 minutes every 3 weeks. It is also FDA-approved for treatment of resected stage III melanoma at a dose of 10mg/kg every three weeks for up to three years.

### 1.6 Nivolumab

PD-1 is an immune checkpoint molecule expressed on activated T cells, NK cells, B cells, monocytes, and dendritic cells.[28] PD-1 is critical for T cell development and regulation in adaptive immune response. PD-1 binds to its ligands PD-L1 and PD-L2, and this leads to immunosuppression, limiting T cell activity in peripheral tissues.

Nivolumab is a fully human, IgG4 (kappa) isotype, monoclonal antibody that binds to programmed death-1 (PD-1) and is approved by the FDA for unresectable or metastatic melanoma, non-small cell lung cancer, renal cell cancer, Hodgkin lymphoma, squamous cell cancer of the head and neck, and urothelial cancer.[29,30,31,32,33] For solid tumors, including melanoma, the approved dose is 240mg IV every 2 weeks or 480mg IV every 4 weeks.

## 2. STUDY OBJECTIVES

### 2.1 Primary Objectives

Evaluate the effect of VX15/2503 in combination with immune checkpoint inhibitors on T cell infiltrate into the tumor microenvironment in involved and uninvolved lymph nodes and peripheral blood.

### 2.2 Secondary Objectives

Evaluate the effect of VX15/2503 in combination with immune checkpoint inhibitors on the immune profile of involved and uninvolved lymph nodes and peripheral blood.

Assess safety and tolerability of profile and tolerability of single agent VX15/2503 to the combination of VX15/2503 and immune checkpoint inhibitors in patients with resectable metastatic melanoma.

Document pathologic response rates of VX15/2503 and immune checkpoint inhibitors in patients with resectable melanoma.

Compare pathologic response to radiographic response using RECIST criteria in patients receiving single agent VX15/2503 and combination VX15/2503 and immune checkpoint inhibitors in patients with resectable melanoma.

## 3. STUDY DESIGN

### 3.1 Pilot integrated biomarker study

This is a neoadjuvant, window of opportunity, trial of VX15/2503 in combination with either ipilimumab or nivolumab. A separate cohort will proceed directly to surgery.

| Disease | Cohort | Treatment | Dose Frequency |
|---|---|---|---|
| Melanoma | A | VX15/2503 (15 mg/kg) | Q3W (days 1, 21) |
| | | Nivolumab (360 mg) | Q3W (days 1, 21) |
| Melanoma | B | VX15/2503 (15 mg/kg) | Q3W (days 1, 21) |
| | | Ipilimumab (3 mg/kg) | Q3W (days 1, 21) |
| Melanoma | C | VX15/2503 (15 mg/kg) | Q3W (days 1, 21) |
| | | Nivolumab (3 mg/kg) | Q3W (days 1, 21) |
| | | Ipilimumab (1 mg/kg) | Q3W (days 1, 21) |
| Melanoma | D | Nivolumab (360 mg) | Q3W (days 1, 21) |
| Melanoma | E | No treatment (SOC) | n/a |

All patients (except cohort E) will undergo surgery between day 35 and 49. Patients in cohort E undergo surgery within 30 days of obtaining consent.

Eight patients will be enrolled into cohorts A, B, C, and D; six patients will be enrolled into cohorts E. Patients will be permitted to choose between one of two groups: no treatment (E) or treatment (A, B, C, or D) until the no treatment group is filled. Each treatment cohort will be enrolled to completion sequentially.

All infusions will have a 3-business window for treatment days.

In the event a patient experiences unacceptable toxicity unrelated to the known toxicity of immunotherapy (Grade 4 non-hematologic or persistent Grade 3 hematologic toxicity), treatment for that arm will be paused (pending further evaluation). Accrual to the trial will also be paused (pending further evaluation) if 2 patients in one treatment arm experience adverse events that delay their surgery (beyond day 49) or ≥ 25% of patients across treatment arms (after enrollment of at least 10 patients) experience adverse events that delay their surgery (beyond day 49). Continued accrual is permitted only if it is determined that the adverse events leading to delay in surgery were not related to treatment or that the delay caused by the event, regardless of attribution, did not result in previously resectable sites of disease to become unresectable.

Following surgery, patients in all cohorts will be offered standard of care nivolumab adjuvant therapy for one year at the discretion of the treating physicians.

### 3.2 Correlative biomarker collection

**Correlative Studies:**
The primary objective for our laboratory correlative studies will be to determine whether treatment with VX15/2503 in combination with immune checkpoint inhibitors (ipilimumab or nivolumab) modulates the phenotypic properties within the tumor microenvironment. We plan to analyze biomarkers guided by data from pre-clinical studies in murine tumor models that demonstrate SEMA4D blockade can synergize with checkpoint inhibition to recruit immune cells into the tumor microenvironment. Patient biopsies obtained at baseline and following surgical resection will be processed and utilized in a series of exploratory correlative laboratory studies to assess potential biomarkers of activity, response, and inform mechanism of action of these treatment regimens.

#### 3.2.1. Collection of Specimens.

Tumor specimens from patients enrolled in the study will be obtained from either baseline diagnostic biopsies or archival tissue obtained <6 months prior to the time of patient enrollment in the trial. A new biopsy will not be required strictly for purposes of this correlative study. Paraffin-embedded tissue will be obtained from the Emory Department of Pathology via Tissue Procurement Services. Surgical tissue will be obtained from all patients following surgery in coordination with Tissue Procurement Services.

Archived (<6 months old) or freshly obtained paraffin embedded and formalin fixed (FFPE) tissues of biopsied or resected specimens are needed for the purposes of:

- Pathological examination (for conventional pathology examination, such as H&E stain)
- Examination of immune and inflammatory fibroblast biomarkers via IHC or IF analysis, as appropriate.
- Future analysis of genomic and transcriptome features within the tumor microenvironment.
- Flow cytometry-based validation of immunohistochemical biomarkers from fresh tissues.

Prioritization of correlative studies is unlikely to be necessary since the quantity of tissue required for study will not be a limiting factor following surgical resection.

**3.2.2. Handling of Specimens.**
These tissues will be prepared as formalin fixed paraffin embedded (FFPE) blocks and used to produce 1 H&E cut at 3micrometers, 30 unstained sections, cut at 4 micrometers and mounted in plus (+) slides, and 30 sections containing at least 50% tumor cut at 10 micrometers and mounted on glass slides. If the tissue is small (< $1cm^2$), 20 unstained sections cut at 10 micrometers of thickness will be required. These latter slides will be air dried (not oven-dried). The slides will be stored at room temperature (>16°C). The sectioning of FFPE tissue for the immunohistochemical studies, and molecular study will be done at the same time as the H&E section and the immunohistochemical sections to minimize tissue loss. The tissue blocks will be stored and fresh sections cut in batches at the end of the study.

Remaining tissue will be archived by the Tissue Procurement Services in the Pathology Department.

It is anticipated that most patients will have the diagnosis of melanoma either by a prior biopsy or excision. Outside available material will be retrieved from the original institution and reviewed for confirmation. This material will be reviewed at Emory University by the Department of Pathology or Dermatopathology. An assessment of the H&E sections will be done to establish the presence of tumor in specific blocks. For patients without a previous diagnosis of melanoma, confirmation of nodal or oligometasatic disease will be performed by fine or core needle biopsy and subject to the same histologic processing.
<u>Histology confirmation requirements.</u> 1 unstained section cut at 3 micrometers for H&E of each block to be tested.
<u>Immunohistochemical stains requirements.</u> A minimum of 30 slides, cut at 3microns unstained sections in plus (+) slides, will be required for the 20 stains.
This outside available material will be received from the original institution to the address below. The research slides and material will be reviewed at the Emory University Pathology laboratories by a Board-Certified Pathologist in the Department of Pathology or Dermatopathology at Emory University.

**3.2.3. Immunohistochemical analysis of tumor tissue.**
Paired tumor biopsies will be used to assess the effect of treatment arm on changes in the melanoma TME. This analysis will be performed on paired samples in n=36 melanoma patients enrolled in the trial. Baseline diagnostic biopsies, when available, will be collected. In addition, specimens from surgical resection will be collected under the guidance of the collaborating pathologist and tissue procurement staff at the time of surgery to capture tissue in excess of that needed for clinical staging and diagnostic purposes.

This design will enable us to isolate the effect of VX15/2503 containing treatment combinations on immune cells, PSC, and stromal components (i.e. Sirius Red to detect collagen) in the TME in the context of anti-PD-1 or anti-CTLA4 antibody. Our primary biomarker of interest will be the extent of CD8+ T cell infiltration between treatment groups following treatment. However, additional assays will be conducted to gather data on changes in other cell subsets that may be relevant to activity of these treatments. Examples of high priority immune and stromal markers are listed in **Table 1**, however additional markers may be added as needed to capture the rapidly evolving understanding of biomarkers relevant to the melanoma stroma and cancer immunology. For instance, this panel can also be expanded for other co-stimulatory or immune checkpoint molecules on cell subsets (i.e. CD8, Tim3, LAG3).

**Table I. Examples of Immunohistochemical (IHC) and Immunofluorescence (IF) biomarkers of interest.**

| IHC Stains | IF Stains |
|---|---|
| CD4 | CD4+FoxP3+ |
| CD8 | CD8+Ki67+ |
| CD11c | CD33+S100+ |
| CD19 | CD4+IL-17+ |
| CD68 | aSMA+FAP+ |
| CD163 | aSMA+IL-6+ |
| PD1 | FAP+IL-6+ |
| CTLA4 | PDGRFβ+IL-6+ |
| PD-L1 | |
| PD-L2 | |
| SEMA4D | |
| Plexin-B1 | |

**3.2.4. Analysis of IHC/IF data.**

After staining, slides will be analyzed in the Integrated Cellular Imaging (ICI) Core Lab to quantitate the percentage and intensity of cells for each marker(s) in melanoma tissue. These data will be compiled for samples, using state-of-the-art Imaris 3D analysis software in the ICI Core Lab. This system allows for true quantitative data on dual staining in patient samples. Given recent literature, and the potential for VX15/2503 to modulate the tumor stroma, this analysis will also help assess if treatment alters cell distribution in TME, and proximity of IL-$6^+$ fibroblast populations to T cells or melanoma. To ensure the rigor of IHC and IF analysis, samples will be batch run when possible, with appropriate positive (i.e. tonsil tissue for immunostains) and negative controls (secondary Ab only) along with serial sections from a single archival pancreatic or colorectal tumor specimen as a qualitative control to for intensity and any batch-to-batch variability between reagents used in IHC or IF assays.

**3.2.5. Analysis of T cell receptor diversity and clonality.**

Formalin-fixed paraffin embedded tissues can be utilized to assess T cell receptor diversity and clonality via survey level immunoseq assay. For these studies, total DNA will be isolated from FFPE tumor tissues and sent to Adaptive Biotechnologies, Inc. for Illumina sequencing of the TCRβ gene in each sample as previously described. Data will be analyzed using the ImmunoSEQ Analyzer (Adaptive Biotechnologies, Inc.).

**3.2.6. Flow cytometric analysis of tumor biopsy tissue.**

When fresh tissue from initial diagnostic biopsy is available (and in all surgical specimens at the study endpoint), IHC/IF will be complemented by a detailed flow-based phenotyping of biopsy tissue to gather more information on immune subsets. Our lab has optimized a protocol to simultaneously analyze multiple immune subsets and obtain quantitative data that correlates with IHC. For these studies, freshly isolated tissue will be dissociated using enzymatic degradation and the GentleMACS system. Resulting homogenates will be assessed and multi-parameter flow cytometry to quantify individual immune subsets present within the tumor microenvironment. A basic flow cytometry panel to phenotype individual cell subsets co-labeled with ZombieViolet Dye and anti-CD45-APC/Cy7 will be utilized to date upon live/dead lymphocytes from the resulting homogenates.

**3.2.7. Excess tumor tissue.**

When sufficient quantity permits, a section of fresh tumor samples will be split and snap frozen in liquid nitrogen either (1) alone for future genomic analysis or (2) in RNALater solution to facilitate future high throughput analysis of gene expression profile. Samples will be stored immediately in cryovials at -80°C for future analysis.

**3.2.8. Blood Specimen Processing.**
Blood samples will be collected prior to treatment at baseline (day 1), day 21, and the day of surgery (day 36 - 49). Three 10mL purple top tubes of blood containing EDTA as an anti-coagulant will be obtained for correlative studies at each of these time points. Blood samples must be processed within 4 hours of the blood draw by courier to the central site (Winship Cancer Institute, Lesinski lab). Blood samples will be centrifuged at room temperature at 805 x *g* for 10 minutes and the plasma layer will be aspirated with a sterile pipet, aliquoted, snap frozen (dry ice or liquid nitrogen) and stored at ≤-70°C until analysis. Plasma samples from individual patient cohorts will be batched and analyzed simultaneously. Serum will also be collected for PK, sSEMA4D, and ADA.

Peripheral blood mononuclear cells will be isolated from these same blood samples following removal of the plasma layer via standard methods using density gradient centrifugation with ficoll-paque plus as described in the laboratory manual. Following isolation PBMCs will undergo phenotypic and functional analysis via flow cytometry staining with excess cells cryopreserved.

**3.2.9. Phenotypic analysis of immunomodulatory cytokines and immune suppressor cell populations.** Elevated levels of pro-inflammatory cytokines and immunosuppressive cell populations are present in patients with cancer. Our group has previously conducted pilot studies and demonstrated that this profile is also operative in peripheral blood from patients with pancreatic cancer. For example, plasma IL-6, IL-10 and MDSC were elevated in patients as compared to normal donors.[34] Furthermore, in a cohort of n=73 treatment naïve patients with metastatic PDAC, plasma IL-6 and IL-10 were correlated with worse overall survival.[35] These factors may reflect an altered cellular milieu present in the tumor microenvironment or systemically that favors reduced anti-tumor immune responses mediated by T lymphocytes.

***3.2.9.a. Cytokine analyses:*** Cytokines in patient plasma will be analyzed in samples obtained at baseline, day 21 and day of surgery using a high-throughput bioplex analysis as described by our group.[38] Our analysis will focus on a panel of cytokines and chemokines that represent the following functional classes: 1) analytes shown by our group as expressed from pancreatic stellate cells; 2) cytokines/chemokines that are documented to promote MDSC or T regulatory cell expansion or migration; 3) cytokines involved in Th1/Th2/Th17 differentiation. All patient samples will be run in duplicate and compared to pre-specified standard curves to obtain quantitative data. Duplicate samples displaying a CV>10% will be re-analyzed with additional replicates for greater accuracy. Given its relevance in both progression of melanoma, a separate aliquot of plasma will be utilized to assess expression of TGF-β via standard ELISA assay as this analyte is not compatible with the proposed bioplex analysis.

***3.2.9.b. Immune suppressor cell analyses:*** The expansion of immune suppressive cytokines and particular cell populations represent important barriers that likely limit the full potential of immune-based therapies or endogenous host responses to tumors. We hypothesize that this state of elevated immune suppressor cells in cancer patients can be manipulated via SEMA4D blockade to provide an advantage for the maximal therapeutic effect of immune checkpoint blockade. For these experiments, multiparameter flow cytometry will be utilized to phenotype immune effector cells to determine whether VX15/2503 containing regimens downregulate T regulatory cells (based on expression of CD4, CD25, CD127, CD49d and FoxP3), IL-10-producing B regulatory cells (based on expression of CD5, CD19, CD1d, CD24, CD25, CD27, CD38 and IL-10) and myeloid derived

suppressor cells ($CD33^{+}HLADR^{-}CD11b^{+}$ plus $CD14^{+}$ or $CD15^{+}$) as these populations can neutralize host anti-tumor responses and could be modulated in response to VX15/2503. Namely, it is possible that VX15/2503 could directly interact with these cells, or the ability of this agent to limit the tumor stroma could result in reduced expansion of these cell populations. Indeed, our group has previously data demonstrating that pancreatic stellate cells are a prominent source of IL-6 in the TME, and that its secretion from these cells can facilitate expansion of suppressive myeloid cell populations.[36] The phenotypes chosen represent the most well-accepted and published phenotypic definitions to date, and will be modified as concordance among the tumor immunology community becomes more apparent.

***3.2.9.c. Analysis of T cell phenotype and function:*** We will also analyze blood from the clinical trial by multi-parameter flow to identify systemic phenotypic shifts mediated by VX15/2503 in combination with immune checkpoint inhibition. This approach has been published by our group, and is continually refined with our collaborator, Dr. Rafi Ahmed, to incorporate the most up-to-date markers of relevance to anti-PD-1 therapy.[38,37] Data obtained in tumor biopsies including proliferative $CD8^{+}CD28^{+}Ki67^{+}$ T cell subsets will be compared to that measured in blood. For analysis of this subset in blood, flow cytometry will be used to assess cells with a more detailed phenotype ($CD8^{+}Ki67^{+}CD28^{+}Bcl2^{lo}CD38^{+}PD\text{-}1^{+}$) or ($CD8^{+}Ki67^{+}CD28^{+}Bcl2^{lo}CD38^{+}CTLA4^{+}$). In addition, we will study how VX15/2503 influences shifts in Th1/Th2/Th17 or Th subsets with phenotypic properties of naïve, central and effector memory cells. These data will be complemented by *ex vivo* analysis of function (intracellular cytokine staining, $Ki67^{+}$ for proliferation) or functional markers (CD107a) in aliquots of freshly isolated T cells stimulated with PMA/Ionomycin or CD3/CD28-beads as per standard protocol.

# 4. SELECTION OF STUDY POPULATION

## 4.1 Inclusion Criteria

Each subject will be counseled by a study investigator about the planned study procedures, risk and benefits of participating in the study, and alternative therapeutic options. Each subject must sign an Institutional Review Board (IRB)/ Independent Ethics Committee (IEC)-approved consent form and written authorization to use protected health information as outlined in the Health Insurance Portability Act (HIPAA) of 1996, prior to the initiation of any study related procedures.

Subjects must meet all of the following inclusion criteria to be eligible for enrollment into the study:

1. Must be at least ≥ 18 years of age
2. Stage IIIB, IIIC, IIID histologically-proven melanoma
    a. Cancer confirmed to be surgically resectable, with surgery evaluation with planned prior to resection.
    b. No prior immunotherapy with CTLA-4, anti PD-1 or VX15/2503. Prior interferon (at least 1 year prior to consent) will be allowed.
3. Eastern Cooperative Oncology Group (ECOG) Performance Status 0 or 1 (Appendix I)
4. Adequate hematologic function as defined by:
    a. Absolute neutrophil count ≥ 1,500 cells/µL
    b. Platelets ≥ 100,000/µL
    c. Hemoglobin ≥ 9.0g/dL (may receive PRBC transfusion)
5. Adequate hepatic function as defined by:

a. Total bilirubin ≤ 1.5 x the upper limit of normal (ULN)
b. AST and alanine aminotransferase (ALT) ≤ 2.5 x ULN
c. Albumin ≥3.0 g/dL

6. Adequate renal function as defined by:
   a. Serum creatinine ≤ 1.5 x ULN OR
   b. Calculated creatinine clearance of ≥ 50 mL/min using Cockcroft-Gault formula

7. INR ≤1.5. Anticoagulation is allowed only with low molecular weight heparin (LMWH). Patient receiving LMW heparin on stable therapeutic dose for more than 2 weeks or with factor Xa level <1.1U/mL are allowed on the trial.

8. Willingness and ability to comply with scheduled visits, treatment plans, laboratory tests, and other study procedures

9. Ability to understand and willingness to sign a written informed consent document

10. Female subjects of childbearing potential must agree to use adequate contraception (at least one highly effective method and one additional method of birth control at the same time or complete abstinence) prior to study entry, for the duration of study treatment and 5 months after last dose of study treatment

11. Male subjects must agree to use adequate contraception (at least one highly effective method and one additional method of birth control at the same time or complete abstinence) prior to study entry, for the duration of study treatment and 7 months after last dose of study treatment

12. Female subjects of childbearing age must have a negative serum pregnancy test at study entry.

Patients in cohort E may proceed directly to surgery without meeting the eligibility criteria 4, 5, 6, and 7 assuming they are deemed candidates and undergo appropriate testing for the planned operation.

### 4.2 Exclusion Criteria

Subjects will be ineligible for enrollment into the study if they meet any of the following criteria:

1. Determined not to be a surgical candidate due to medical co-morbidities
2. Treatment with chronic immunosuppressants (e.g., cyclosporine following transplantation).
3. Prior organ allograft or allogeneic bone marrow transplantation.
4. Subjects with active or history of immune mediated pneumonitis, colitis, hepatitis, endocrinopathy, nephritis, or skin reactions as these patients may be at increased risk for developing immune therapy-induced exacerbation or recurrence of their immune mediated disease, potentially delaying surgery.
5. Subjects with a condition requiring systemic treatment with either corticosteroids (> 10mg daily prednisone equivalents) or other immunosuppressive medications within 14 days of study drug administration. Inhaled or topical steroids and adrenal replacement doses > 10mg daily prednisone equivalents are permitted in the absence of active autoimmune disease.

6. Women who are pregnant or lactating
7. Uncontrolled intercurrent illness including, but not limited to, human immunodeficiency virus (HIV)-positive subjects receiving combination antiretroviral therapy, ongoing or active infection, symptomatic congestive heart failure (NYHA Class III or IV), unstable angina pectoris, ventricular arrhythmia, or psychiatric illness/social situations that would limit compliance with study requirements
8. Other medications, or severe acute/chronic medical or psychiatric condition, or laboratory abnormality that may increase the risk associated with study participation or study drug administration, or may interfere with the interpretation of study results, and in the judgment of the investigator would make the subject inappropriate for entry into this study
9. Clinical evidence of bleeding diathesis or coagulopathy
10. Patients with prior malignancies, including pelvic cancer, are eligible if they have been disease free for > 5 years. Patients with prior non-melanoma skin cancers and in situ carcinomas are eligible provided there was complete removal.
11. Active bacterial or fungal infections requiring systemic treatment within 7 days of treatment.
12. Use of other investigational drugs (drugs not marked for any indication) within 28 days or at least 5 half-lives (whichever is longer) before study drug administration.
13. History of severe hypersensitivity reactions to other monoclonal antibodies
14. Non-oncology vaccines within 28 days prior to or after any dose of ipilumumab.
15. Prisoners and subjects who are compulsory detained
16. Patients with rapidly progressive disease

Screen Failures

Minimal data for subjects who fail screening will be collected such as demographic information and the reason for screen failure. Such subjects may be re-screened at the discretion of the investigator after approval by the Data and Safety Monitoring Committee (DSMC). The reason for the need to re-screen a subject will be documented in the subject's source documents.

# 5. TREATMENT OF SUBJECTS

## 5.1 Study Procedures

**Screening Visit**

All subjects must sign an informed consent document prior to the initiation of any study related procedures. The informed consent document must be signed within 28 days of day 1 of treatment. The screening procedures (with the exception of the scans and consent) are to be conducted within 14 days of treatment start date.

- Review of study eligibility criteria

- Medical History
- Record concomitant medications taken up to 14 days prior to day 1
- Vitals [temperature, heart rate (HR), blood pressure (BP) and respiratory rate (RR), oxygen saturation]
- Physical Examination, including height and weight
- ECOG Performance Status evaluation
- Laboratory Assessments (not required for patients in cohort E)
    - Hematology: hemoglobin, hematocrit, white blood cell count with differential and platelet count
    - Serum chemistry: sodium, potassium, chloride, bicarbonate, magnesium, calcium, phosphorus, blood urea nitrogen, creatinine, glucose (random), total bilirubin, LDH, total protein, ALP, ALT, AST, amylase, lipase, and albumin
    - Thyroid function panel: TSH, free T3, free T4.

      (Subjects suspected to have autoimmune thyroid disorders will also need to be tested for thyroglobulin, and thyroid peroxidase antibodies and thyroid stimulating immunoglobulin.)
    - Serum pregnancy test for women of childbearing potential
    - Prothrombin time (PT) and activated partial thromboplastin time (aPTT)
    - Urinalysis

- 12-lead ECG (not required for patients in cohort E)
- Radiologic imaging studies to evaluate tumor status: Brain MRI and positron emission tomography/computed tomography (PET-CT) or contrast computed tomography (CT) of the chest and abdomen and pelvis. Additional imaging may be obtained as clinically indicated. Baseline scans must be done within 4 weeks prior to the first dose of study drug, with the exception of cohort E.

- Tumor tissue from diagnostic biopsy, if available, is provided for biomarker analysis.

**Treatment and toxicity follow-up phase**

#### 5.1.1.1 Days 1 and 21 (Visits can occur ± 3 business days of scheduled visits)

- Record concomitant medications
- Vitals (temperature, HR, BP and RR)
- Physical Examination and ECOG evaluation
- Laboratory Assessments
    - Hematology hemoglobin, hematocrit, white blood cell count with differential and platelet count
    - PT, aPTT (day 1 only)

- Chemistry sodium, potassium, chloride, bicarbonate, magnesium, calcium, phosphorus, blood urea nitrogen, creatinine, glucose (random), total bilirubin, LDH, total protein, ALP, ALT, AST, amylase, lipase, and albumin
- TSH, Free T4, free T3

- Treatment with VX15/2503 and nivolumab, VX15/2503 and ipilimumab, VX15/2503/nivolumab/ipilimumab, or nivolumab
- 1 hour post-transfusion monitoring period
- Record AEs
- Blood samples (prior to treatment on days 1 and 21)
    - 2 x 10ml collected in purple top (EDTA) tubes (total 20ml)
    - 3 x 2.5ml collected in gold top SST tubes (total 7.5ml)
    - 2 x 5ml collected in cytochex tubes (total 10ml)
- Blood samples for correlative work (days 1 and 21 after treatment)
    - 3 x 2.5ml collected in gold top SST tubes (total 7.5ml)
    - 2 x 5ml collected in cytochex tubes (total 10ml)

#### 5.1.1.2 Within 7 days prior to surgery (day 35-49)

- Brain MRI and positron emission tomography/computed tomography (PET-CT) or contrast computed tomography (CT) of the chest and abdomen and pelvis. The imaging should be consistent with baseline imaging. Imaging will be interpreted for radiographic response using RECIST 1.1. Repeat imaging is not required for patients in cohort E.
- Blood sample for correlative work:
    - 2 x 10ml collected in purple top (EDTA) tubes (total 20ml)
    - 3 x 2.5ml collected in gold top SST tubes (total 7.5ml)
    - 2 x 5ml collected in cytochex tubes (total 10ml)

#### 5.1.1.3 Surgery day (day 35-49)

- Surgical specimen collection

### 5.2 End of Treatment Visit

End of treatment visit should be completed 4 to 8 weeks after surgery. This visit is not required for patients in cohort E.

- Record concomitant medications
- Vitals (temperature, HR, BP and RR)
- Physical Examination, including weight. Specifically, monitor for any autoimmune side effects that may have occurred in the following organ sites:
    - Gastrointestinal

- Renal
- Pulmonary
- Hepatic
- Endocrinopathies
- Skin
- Neurological

- ECOG Performance Status evaluation
- Laboratory Assessments
  - Hematology hemoglobin, hematocrit, white blood cell count with differential and platelet count
  - Chemistry sodium, potassium, chloride, bicarbonate, magnesium, calcium, phosphorus, blood urea nitrogen, creatinine, glucose (random), total bilirubin, LDH, total protein, ALP, ALT, AST, and albumin
  - Thyroid function panel: TSH with reflex to free T3 and T4 if TSH abnormal.

- Record AEs, CT or MRI of chest, abdomen, and pelvis
- Blood sample for correlative work:
  - 3 x 2.5ml collected in gold top SST tubes (total 7.5ml)
  - 2 x 5ml collected in cytochex tubes (total 10ml)

### 5.3 Survival Follow-up Assessment

Subject survival information will be collected, preferably via office visit or telephone contact, from end of treatment on day 90 (± 7 days), and then every 12 weeks (± 3 weeks) for 2 years, then every 6 months for next 3 years, then annually until the subject's death or until the subject is lost to follow-up up to 10 years. No survival follow-up data will be collected for Cohort E subjects.

A subject will be determined to be lost to follow-up only after at least 3 attempts to contact him/her have been made over a 4-month period. At least two of these attempts to contact must be made through certified letters.

The following information will be collected:

- Disease status as: 1. No evidence of disease on treatment (document treatment type if available), 2. No evidence of disease on follow up, 3. Active disease on treatment (document treatment type if available), 4. Active disease not on treatment
- The subject's survival status, and if deceased, the date of death. If known, cause of death.
- The method by which the survival status was assessed and the date it was assessed.
- Any subsequent anti-cancer therapy received or imaging performed after completion of study.
- Blood sample for correlative work (to be collected if within 100 days from last treatment):
  - 3 x 2.5ml collected in gold top SST tubes (total 7.5ml)

    - 2 x 5ml collected in cytochex tubes (total 10ml)
- Specifically, monitor for any autoimmune side effects that may have occurred in the following organ sites if within the 100 days from last treatment.
    - Gastrointestinal
    - Renal
    - Pulmonary
    - Hepatic
    - Endocrinopathies
    - Skin
    - Neurological

### 5.4 Schedule of Events

| Study Day[1] | Screen[2] | Days 1, 21 | Days 35-49 | EOT[3] | Survival Follow-up[5] |
|---|---|---|---|---|---|
| Informed Consent | X | | | | |
| Review of eligibility | X | | | | |
| Medical History | X | X | | X | X |
| Concomitant Meds | X | X | | X | |
| Vital signs | X | X | | X | |
| Physical Exam/ ECOG PS | X | X | | X | |
| Toxicity Monitoring | X | X | | X | |
| Hematology & Chemistry and LFT | X | X | | X | |
| Lipase, Amylase | X | X | | X | |
| Thyroid function panel | X | | | X | |
| Serum pregnancy test for females of child-bearing potential | X | X (DAY 21)[13] | | | |
| PT, aPTT | X | X | | X | |
| Urinalysis | X | | | | |
| ECG[4] | X | | | | |
| BRI MRI and PET/CT or CT of Neck, Chest, Abdomen & Pelvis | X | | X[9] | X | |
| Treatment | | X[10] | | | |
| Surgery | | | X[11] | | |
| Tissue Biopsy | X | | | | X[12] |
| Surgical specimen collection | | | X[11] | | |
| Blood for correlative assays[6] | | X | X | | X[12] |
| Serum (PK, sSEMA4D, ADA)[7] | | X | X | X | X[12] |
| Whole blood for SEMA4D saturation[8] | | X | X | X | X[12] |

1. +/- 3 business day visit window can be applied to all scheduled visits (with the exception of the screening visit).
2. All screening procedures should be completed within 14 days of Day 1 unless specified otherwise. Labs and ECG are not required for patients in cohort E. Baseline imaging may be more than 28 days from surgery for patients in cohort E.
3. End of treatment (EOT) visit should be completed 4 to 8 weeks after surgery. This visit is not necessary for patients in cohort E.
4. Additional ECG(s) to be performed as clinically indicated at any point during or after treatment.
5. Subject survival information will be collected, preferably via office visit or telephone contact, on day 90 (± 7 days), and then every 12 weeks (± 3 weeks) for 2 years, then every 6 months for next 3 years, then annually for up to 10 years, or until the subject dies or is lost to follow-up.
6. Blood collection should be prior to receiving treatment on day 1, day 21, and 7 days before surgery: 2x10 ml collected in purple top EDTA tubes at each timepoint
7. Serum for PK, sSEMA4D, and ADA will be collected pre-dose and end of all infusions on Day 1 and 21, within 7 days before surgery and at the EOT and survival follow up visit: 3x 2.5 ml collected in gold top SST tubes at each timepoint

8. Blood for cellular SEMA4D saturation will be collected pre-dose and end of infusions on Day 1 and 21, within 7 days before surgery, at the EOT and survival follow up visit. 2x 5ml collected in cytochex tubes at each timepoint
9. Repeat imaging should be obtained within 7 days of surgery. Imaging will be evaluated for radiographic response using RECIST 1.1. Imaging should include target lesion with either PET scan or CT neck/chest/abd/pelvis, whichever is necessary.
10. Infusion window of +/-3days
11. Surgery would be on days 35-49
12. Only required if patient is doing survival follow up in clinic and for the first year post treatment. If survival follow up is from outside notes and telephone, the blood samples are not required. Or if the patient is outside the 1-year post-treatment window, then blood samples are not required. Tissue biopsy will only be performed if there is suspicion of recurrence within the first year post-surgery. A new biopsy will not be required strictly for the purposes of correlative studies.
13. For patients that proceed to standard of care adjuvant therapy, pregnancy tests for patients' of child bearing potential should be tested every four weeks.

## 5.5 Pre- and Post-Treatment Tumor Sample collection

For baseline, pre-treatment tumor samples, archived tumor samples that were obtained for diagnostic purposes will be collected. New biopsies will not be necessary for enrollment to the study. Tumor specimens collected at surgery (day 35-49) will be the post-treatment tumor samples. Tumor samples will be formalin-fixed, paraffin-embedded, and sectioned for preparation of slides that will be stained with H&E for confirmation of tumor. Fresh tissue of the post treatment tumor samples will be preserved as fresh frozen tissue placed in buffers that contain phosphatase inhibitors as described in Section 3.2.

## 5.6 Dose Modifications and Stopping treatment

**The second treatment may be held if patients experience any of the following adverse events. These patients will proceed directly to surgery at the discretion of the PI and treating surgeon. If 2 patients on one treatment arm experience adverse events that delay their surgery (beyond day 60), we will halt accrual of that treatment arm. Continued accrual is only permitted if it is determined that the adverse events leading to delay in surgery were not related to treatment.** In past experience, toxicities have been observed after >6-month treatment with immunotherapy agents, and thus toxicity risk is low in this short study in which patients will be receiving only 2 doses of the immunotherapy agents. In the setting of grade 3 or higher AE during neoadjuvant treatment, adjuvant therapy will be determined by the treating investigator and approved by the PI.

VX15/2503, Nivolumab or ipilimumab should be stopped or delayed up to one week for the following:

- Any Grade ≥2 drug-related uveitis, eye pain, or reduction in visual acuity that does not respond to topical therapy or requires systemic treatment
- Any Grade ≥ 2 non-skin, drug-related adverse event, with the following exceptions:
  - Grade 2 drug-related fatigue or laboratory abnormalities
- Any Grade ≥3 non-skin, drug-related adverse events including but not limited to:
  - Drug-related pneumonitis, bronchospasm, neurological toxicity, hypersensitivity reaction, infection reaction
  - Drug-related endocrinopathies not adequately controlled with physiologic hormone replacement
  - Drug-related thrombocytopenia > 7 days or associated with bleeding
- Any drug-related liver function test abnormality that meets the following criteria
  CTACE v4.0 Grade ≥ 3 AST, ALT, or Total Bilirubin
- Any Grade ≥ 3 drug-related laboratory abnormality, with the following exceptions:
  - Grade 3 amylase or lipase abnormalities that are not associated with symptoms or clinical manifestations of pancreatitis do not require stopping treatment
  - Grade 3 lymphopenia does not require stopping treatment
- Any Grade 3-4 skin reactions.
- Any adverse event, laboratory abnormality, or intercurrent illness which, in the judgement of the investigator, warrants interrupting the dose of study medication.

## 5.7 Management of adverse effects

**Treatment of infusion reactions**

Infusion reactions should be graded according to CTCAE Version 4.0 allergic reaction/hypersensitivity. Since nivolumab and ipilimumab contain only human immunoglobulin protein sequences, it is unlikely to be immunogenic and induce infusion or hypersensitivity reactions. However, if such a reaction were to occur, these reactions may manifest with signs and symptoms that may include, but are not limited to fever, chills, headache, rash, pruritus, arthralgias, hypotension, hypertension, bronchospasm or other symptoms. Severe infusion reactions require the immediate interruption of study drug therapy and permanent discontinuation from further treatment. Appropriate medical therapy including epinephrine, corticosteroids, intravenous antihistamines, bronchodilators, and oxygen should be available for use in the treatment of such reactions. Subjects should be carefully observed until the complete resolution of all signs and symptoms. Following an infusion reaction, subjects should be premedicated with acetaminophen and diphenhydramine for future treatments.

All Grade 3 or 4 infusion reactions should be reported within 24 hour and reported as SAE if criteria are met.

Treatment recommendations are provided below and may be modified based on local treatment standards and guidelines, as appropriate:

**For Grade 1 symptoms: (Mild reaction; infusion interruption not indicated; intervention not indicated)**
Remain at bedside and monitor subject until recovery from symptoms.

**For Grade 2 symptoms: (Moderate reaction requires therapy or infusion interruption but responds promptly to symptomatic treatment [eg, antihistamines, non-steroidal anti-inflammatory drugs, narcotics, corticosteroids, IV fluids]; prophylactic medications indicated for ≤ 24 hours)**
Stop the nivolumab or ipilimumab infusion, begin an IV infusion of normal saline, and treat the subject with diphenhydramine 50 mg IV (or equivalent) and/or acetaminophen 325 to 1000 mg remain at bedside and monitor subject until resolution of symptoms.

Corticosteroid therapy may also be administered as appropriate. If the infusion is interrupted, then restart the infusion at 50% of the original infusion rate when symptoms resolve; if no further complications ensue after 30 minutes, the rate may be increased to 100% of the original infusion rate. Monitor subject closely. If symptoms recur then no further nivolumab or ipilimumab will be administered at that visit. Administer diphenhydramine 50 mg IV, and remain at bedside and monitor the subject until resolution of symptoms. The amount of drug infused must be recorded on the case report form (CRF). If necessary, corticosteroids (up to 25 mg of SoluCortef or equivalent) may be used.

**For Grade 3 or Grade 4 symptoms: (Severe reaction, Grade 3: prolonged [ie, not rapidly responsive to symptomatic medication and/or brief interruption of infusion]; recurrence of symptoms following initial improvement; hospitalization indicated for other clinical sequelae [eg, renal impairment, pulmonary infiltrates]. Grade 4: life-threatening; pressor or ventilatory support indicated).**

Immediately discontinue infusion of nivolumab or ipilimumab. Begin an IV infusion of normal saline, and treat the subject as follows: Recommend bronchodilators, epinephrine 0.2 to 1 mg of a 1:1,000 solution for subcutaneous administration or 0.1 to 0.25 mg of a 1:10,000 solution injected slowly for IV administration, and/or diphenhydramine 50 mg IV with methylprednisolone 100 mg IV (or equivalent), as needed. Subject should be monitored until the investigator is comfortable that the symptoms will not recur. Nivolumab or ipilimumab will be permanently discontinued. Investigators should follow their institutional guidelines for the treatment of anaphylaxis. Remain at bedside and monitor subject until recovery from symptoms. In the case of late-occurring hypersensitivity symptoms (eg, appearance of a localized or generalized pruritus within 1 week after treatment), symptomatic treatment may be given (eg, oral antihistamine, or corticosteroids).

**5.72 Management of side effects**

Immunotherapy agents are associated with adverse events that can differ in severity and duration than adverse events caused by other therapeutic classes. In addition, VX15/2503 and ipilimumumab and nivolumab may have overlapping autoimmune toxicities. Management algorithms have been developed to assist investigators in assessing and managing the following groups of adverse events:

- Gastrointestinal
- Renal
- Pulmonary
- Hepatic
- Endocrinopathies
- Skin
- Neurological

**Gastrointestinal AE Management:**

| | | |
|---|---|---|
| **Grade 1:**<br>Diarrhea: <4 stools/day over baseline<br><br>Colitis: asymptomatic | **Management**:<br>Symptomatic treatment, if worsen treat as Grade 2 or 3/4 | **Follow-up:**<br>If worsen or persists for more than 3-5 days with steroids, treat as Grade 3-4. |
| **Grade 2:**<br>Diarrhea: 4-6 stools per day over baseline, IV fluids indicated <24 hours, not interfering with activities of daily living (ADL)<br><br>Colitis: abdominal pain; blood in stool | **Management:**<br>Symptomatic treatment. | **Follow-up:**<br>If persists for more than 5 days or recurs, start 0.5-1.0 mg/kg/day methylprednisolone or oral equivalent<br>When symptoms improve to grade 1, taper steroids over at least 1 month, and consider prophylactic antibiotics for opportunistic infections |

| | | |
|---|---|---|
| **Grade 3-4:**<br>Diarrhea (G3) $\geq$ 7 stools per day over baseline, incontinence, $\geq$24 hours, interfering with ADL<br><br>Colitis (G3): severe abdominal pain, medical intervention indicated, peritoneal signs<br><br>G4: life-threatening, perforation | **Management:** 1-2mg/kg/day methylprednisolone IV or IV equivalent.<br>Add prophylactic antibiotics for opportunistic infections.<br>Consider lower endoscopy | **Follow-up:**<br>If improves: continue steroids, then taper over at least 1 month<br>If persists for more than 3-5 days or recurs after improvement: Add infliximab 5mg/kg (if no contraindication). Infliximab should not be used in the cases of perforation or sepsis |

**Renal AE Management:**

| | | |
|---|---|---|
| **Grade 1:**<br>Creatinine > ULN and > than baseline but ≤ 1.5x baseline | **Management**:<br>Monitor creatinine weekly | **Follow-up:**<br>If worsen treat as Grade 2 or Grade 3-4. |
| **Grade 2-3:**<br>Creatinine > 1.5x baseline to ≤ 6x ULN | **Management:**<br>Monitor creatinine every 2-3 days<br>0.5-1.0 mg/kg/day methylprednisolone IV or oral equivalent<br>Consider renal biopsy | **Follow-up:**<br>If returns to Grade 1:<br>Taper steroids over at least 1 month, consider prophylactic antibiotics for opportunistic infections.<br>If elevations persist for more than 7 days or worsen:<br>Treat as Grade 4 |
| **Grade 4:**<br><br>Creatinine >6x ULN | **Management:**<br>Monitor creatinine daily<br>1-2mg/kg/day methylprednisolone IV or IV equivalent.<br>Consult nephrologist<br>Consider renal biopsy | **Follow-up:**<br>If returns to grade 1: Taper steroids over at least 1 month and add prophylactic antibiotics for opportunistic infections. |

**Pulmonary AE Management:**

| | | |
|---|---|---|
| **Grade 1:**<br><br>Radiographic changes only | **Management**:<br>Monitor symptoms for 2-3 days<br>Consider Pulmonary and Infectious Disease (ID) consults. | **Follow-up:**<br>Re-image at least every 3 weeks<br>If worsens: Treat as Grade 2 or Grade 3-4 |
| **Grade 2:**<br><br>Mild to moderate new symptoms | **Management:**<br>Pulmonary and ID consults<br>Monitor symptoms daily, consider hospitalization<br>0.5-1.0 mg/kg/day methylprednisolone IV or oral equivalent<br>Consider bronchoscopy, lung biopsy | **Follow-up:**<br>Re-image every 1-3 days<br>If improves: When symptoms return to near baseline, taper steroids over at least 1 month and consider prophylactic antibiotics.<br>If not improving after 2 weeks or worsening: Treat as Grade 3-4 |
| **Grade 3-4:**<br><br>Severe new symptoms; New/worsening hypoxia; Life-threatening | **Management:**<br>Hospitalize<br>Pulmonary and ID consults<br>2-4mg/kg/day methylprednisolone IV or IV equivalent.<br>Add prophylactic antibiotics for opportunistic infections<br>Consider bronchoscopy, lung biopsy | **Follow-up:**<br>If returns to baseline: Taper steroids over at least 6 weeks<br>If not improving after 48 hours or worsening: Add additional immunosuppression (eg infliximab, cyclophosphamide, intravenous immunoglobulin, or mycophenolate mofetil. |

**Hepatic AE Management**

| | | |
|---|---|---|
| **Grade 1:**<br><br>AST or ALT > ULN to 3x ULN and/or Total bilirubin (Tbili) >ULN to 1.5X ULN | **Management**: No intervention. | **Follow-up:**<br>Continue LFT monitoring<br>If worsens: Treat as Grade 2 or Grade 3-4 |
| **Grade 2:**<br><br>AST or ALT >3.0 to ≤5 x ULN and/or Tbili >1.5 to 3 ≤ ULN | **Management:**<br>Increase frequency of monitoring to every 3 days | **Follow-up:**<br>If returns to baseline: Resume monitoring<br><br>If elevations persist for more than 5-7 days or worsen: 0.5-1.0 mg/kg/day methylprednisolone or oral equivalent and when LEFT returns to grade 1 or baseline, taper steroids over at least 1 month, consider prophylactic antibiotics for opportunistic infections |
| **Grade 3-4:**<br><br>AST or ALT > 5x ULN and/or Tbili >3x ULN | **Management:**<br>Increase frequency of monitoring to every 1-2 days<br>1-2mg/kg/day methylprednisolone IV or IV equivalent.<br>Add prophylactic antibiotics for opportunistic infections<br>Consult gastroenterologist | **Follow-up:**<br>If returns to grade 2: Taper steroids over at least 4 weeks<br><br>If not improving in >3-5 days, worsens, or rebounds: Add mycophenolate mofetil 1 gram twice daily.<br>If no response within an additional 3-5 days, consider other immunosuppressants per local guidelines. |

## Endocrinopathy Management

| | | |
|---|---|---|
| Asymptomatic TSH elevation | **Management**:<br>If TSH <0.5 x LLN or TSH >2 ULN, or consistently out of range in 2 subsequent measurements: include fT4 at subsequent cycles as clinically indicated; consider endocrinology consult | |
| Symptomatic endocrinopathy | **Management:**<br>Evaluate endocrine function<br>Consider pituitary scan<br><br>Symptomatic with abnormal lab/pituitary scan:<br>Initiate appropriate hormone therapy<br>Use of high dose steroids will be at the discretion of the treating investigators with discussion with the PI.<br><br>No abnormal lab/pituitary MRI scan but symptoms persist:<br>Repeat labs in 1-3 weeks/ MR in 1 month | **Follow-up:**<br>If improves (with or without hormone replacement):<br>Taper steroids over at least 1 month and consider prophylactic antibiotics for opportunistic infections.<br><br>Patients with adrenal insufficiency may need to continue steroids with mineralocorticoid component. |
| Suspicion of adrenal crisis (eg severe dehydration, hypotension, shock out of proportion to current illness) | **Management:**<br>Rule out sepsis<br>Stress dose of IV steroids with mineralocorticoid activity<br>IV fluids<br>Consult endocrinologist<br>If adrenal crisis ruled out, then treat as above for symptomatic endocrinopathy | |

**Skin AE Management**

| | | |
|---|---|---|
| Grade 1-2<br><br>Covering ≤ 30% body surface area (BSA) | **Management**: Symptomatic therapy eg antihistamines, topical steroids | If persists > 1-2 weeks or recurs:<br>Consider skin biopsy<br>Consider 0.5-1mg/kg/day methylprednisolone IV or oral equivalent. Once improving, taper steroids over at least 1 month, consider prophylactic antibiotics for opportunistic infections.<br>If worsens: Treat as Grade 3-4 |
| Grade 3-4<br><br>Covering >30% BSA; Life threatening consequences | **Management:**<br>Consider skin biopsy<br>Dermatology consult<br>1-2mg/kg/day IV methylprednisolone IV or IV equivalent | **Follow-up:**<br>If improves to Grade 1:<br>Taper steroids over at least 1 month and add prophylactic antibiotics for opportunistic infections |

## **Neurological AE Management**

Rule out non-inflammatory causes. If non-inflammatory cause, treat accordingly and continue immunotherapy.

| | | |
|---|---|---|
| Grade 1<br><br>Asymptomatic or mild symptoms; Intervention not indicated | **Management**: No intervention | Continue to monitor the patient<br>If worsens:<br>Treat as Grade 2 or 3-4 |
| Grade 2<br><br>Moderate symptoms; Limiting instrumental ADL | **Management:**<br>Treat symptoms per local guidelines<br>Consider 0.5-1.0 mg/kg/day methylprednisolone IV or PO equivalent | **Follow-up:**<br>If improves to baseline:<br>No intervention<br>If worsens:<br>Treat as Grade 3-4 |
| Grade 3-4<br><br>Severe symptoms; Limiting self-care ADL; Life-threatening | **Management:**<br>Obtain neurology consult<br>Treat symptoms per local guidelines<br>1-2mg/kg/day IV methylprednisolone or IV equivalent<br>Add prophylactic antibiotics for opportunistic infection | If improves to Grade 2:<br>Taper steroids over at least 1 month<br><br>If worsens or atypical presentation:<br>Consider IVIG or other immunosuppressive therapies per guidelines |

### 5.8 Duration of Treatment

A subject's duration of participation will include a 2-week screening period and 6-week treatment period immediately followed by surgery (day 35-49) except for patients in cohort E who will proceed to surgery within 28 days of signing consent.

### 5.9 Subject Replacements

Patients who do not complete the intended course of immunotherapy due to reasons other than toxicity or who do not undergo surgery will be considered as in-evaluable for the primary endpoint and will be replaced.

### 5.10 Removal of Subjects from Therapy or Assessment

A subject may discontinue or be discontinued from study drug for any of the following reasons:

- Subject deceased
- Progressive disease based on RECIST
- Symptomatic deterioration
- AE

- Protocol violation / non-compliance
- Withdrew consent from treatment
- Investigator's decision
- Lost to follow-up

The reason for withdrawal shall be recorded by the Investigator on the relevant page of the case report form. All subjects who withdraw from study drug prematurely will undergo end-of-treatment assessments within 20 to 30 days after the last dose, if possible. Regardless of the reason for withdrawal, effort shall be made to follow safety events from the time of withdrawal through resolution or until the event stabilizes. Subjects who discontinue study drug will continue to be followed for survival every 12 weeks (± 3 weeks) from the date of last dose of study drug until the subject's death, or until the subject is lost to follow-up, or until study closure (approximately 6 months after the last subject terminates treatment).

### 5.11 Method of Assigning Subjects to Treatment Groups

This is an open-label Phase I, integrated biomarker study; no blinding will be performed. The patients will be assigned to treatment groups via process described above until all arms are filled.

# 6 TEST ARTICLE AND ADMINISTRATION

## 6.1 VX15/2503

VX15/2503 will be supplied by Vaccinex, the sponsor. VX15/2503 is formulated for intravenous administration as a sterile solution of 20 mg/mL antibody in 20 mM sodium acetate, pH 5.4, containing 130 mM sodium chloride and 0.02% polysorbate 80; the formulation contains no preservatives. The product is supplied in 10 mL vials containing 10 mL at a concentration of 20 mg/mL; each vial is intended for single use. The product packaging consists of 10 mL USP Type I Schott glass vials with West Pharmaceutical Services FluroTec®greybutyl rubber stoppers and a blue plastic cap with aluminum overseal. Product vials are packaged in cardboard boxes and should be protected from light. The drug product is a clear to slightly opalescent, colorless to faintly yellow solution, essentially free from visible particulates; the solution is a preservative-free liquid concentrate for intravenous administration.

<u>Storage</u>
Stability data indicate that both VX15/2503 formulated bulk drug substance and drug product are stable for at least 36 months when stored at 5 ± 3°C.
VX15/2503 should be stored at 5 ± 3°C and protected from light.

<u>Preparation for Administration</u>
Using aseptic technique, prepare the solution for infusion as follows:
The vialed drug should be carefully inspected for discoloration and for particulates. The solution of VX15/2503 and its placebo are normally clear to slightly opalescent, colorless or faintly yellow, and may contain a small amount of visible particulates. If the solution is discolored or otherwise does not meet this description the drug should not be administered and instead the study sponsor should be contacted. The vialed solution contains no preservatives. Do not shake the vialed solution.

To prepare infusion solution, withdraw the required amount of VX15/2503 and dilute the solution with 0.9% sodium chloride injection, USP, as needed. Doses of 2000 mg and above should be diluted to a total volume of 250mL with 0.9% sodium chloride injection, USP. Do not exceed a final protein concentration of 10 mg/mL and do not prepare solutions with a final protein concentration of less than 1 mg/mL. Mix the diluted solution by gentle inversion; do not shake. Inspect the prepared solution visually for particulates prior to administration.

Destroy any unused vialed material according to organizational procedures for the destruction of biologic agents. If no appropriate procedure exists, return the unused vialed solution to the study drug distributor for destruction.

<u>Administration</u>
Administration of VX15/2503 should occur as soon as possible following dilution. If a delay is anticipated, the prepared solution may be kept at room temperature for no more than 8 hours prior to infusion, or for not more than 24 hours after dilution if stored at 5 ± 3ºC (36 to 46ºF). Do not freeze the diluted solution.
*VX15/2503 should be administered prior to either nivolumab or ipilimumab. 30 minutes should lapse between administration of VX15/2503 and either nivolumab or ipilimumab.
* Patients should remain 1 hour after all drugs are infused for monitoring with vital signs q15min.

*Use one of the following (or comparable) infusion sets:*

- *Hospira product number 12030-12 (polyvinylchloride composition)* (with or without in-line filter)
- Baxter product number 2C8875 (*polyvinylchloridecomposition* with polyethylene lined tubing) (with or without in-line filter)

Administer the prepared intravenous solutions using an infusion pump. Infuse all doses of VX15/2503 over 60 minutes (± 5 minutes) using a peripheral intravenous line or indwelling intravenous catheter. Flush the line with 0.9% sodium chloride injection, USP, after study drug infusion.

Do not administer VX15/2503 an infusion with intravenous solutions of other medicinal products. Do not add other medications to solutions containing VX15/2503 or placebo.

Precautions for anaphylaxis should be observed during VX15/2503 administration. Due to the possibility that allergic or infusion reactions may occur, emergency resuscitation equipment (eg, a "crash cart") and medications should be present for immediate use in the immediate area where subjects are receiving their infusions. Additional supportive measures should be available and may include, but are not limited to: epinephrine, antihistamines, corticosteroids, IV fluids, vasopressors, oxygen, bronchodilators, diphenhydramine, and acetaminophen.

*Management of Infusion Reactions and Cytokine Release Syndrome*

An acute infusion reaction may occur with an agent that causes cytokine release (eg, monoclonal antibodies or other biological agents). Signs and symptoms usually develop during or shortly after drug infusion and generally resolve completely within 24 hours of completion of infusion. Signs and symptoms may include: allergic reaction/hypersensitivity (including drug fever); arthralgia, bronchospasm, cough, dizziness, dyspnea, fatigue, headache, hypertension, hypotension, myalgia, nausea, pruritus, rash/desquamation, rigors/chills, diaphoresis, tachycardia, tumor pain, urticaria, and vomiting.

The following are treatment guidelines for VX15/2503 treatment-related infusion reactions:

**Grade 1 or 2:**

- Discontinue the infusion and administer acetaminophen and/or diphenhydramine and/or dexamethasone to treat signs and symptoms if clinically indicated.
- Once symptoms have resolved, slow the infusion rate by 50% of the baseline rate.
- If, after 1 hour, the subject's symptoms do not return and vital signs are stable, the infusion rate of VX15/2503 may be increased every 30 minutes as tolerated to the baseline rate.
- Vital signs, including hear rate, blood pressure, pulse oximetry and temperature, should be measured every 15 minutes or less as clinically indicated. For subjects who are able to tolerate an increase in the infusion rate back to Baseline and maintain normal blood pressure for 30 minutes after the rate increase, then, at the discretion of the Investigator, the frequency of vital sign assessment may be reduced to every 30 minutes during the infusion.

- Monitor the subject for worsening of condition; if severity of event increases to a higher Grade (Grade 3 or 4), then stop the infusion, administer appropriate treatment, and refer to guidelines below for Grades 3 or 4 infusion reactions.

- Subjects with maximum Grade 2 infusion reaction may continue on study and should receive prophylactic premedication with oral acetaminophen 650 mg and diphenhydramine hydrochloride 25 to 50 mg IV (or with a premedication regimen according to the investigational site standard of care) prior to all subsequent VX15/2503 infusions.

**Grade 3 or 4:**

- Stop the infusion and disconnect the infusion tubing from the subject. Do not flush the infusion line.

- Administer diphenhydramine hydrochloride 50 mg IV, dexamethasone 10 to 20 mg IV (or equivalent), and other medications/treatment as medically indicated.

- Give epinephrine or bronchodilators as indicated.

- Hospital admission for observation may be indicated.

- Subjects with Grade 3 or 4 infusion reactions or Grade 3 or 4 anaphylaxis should not receive further VX15/2503 treatment, but will continue to be followed on the protocol.

- Obtain blood sample for cytokine analysis during the event and approximately 24 hours later.

- The event should be reported as an SAE.

- An ADA (anti-drug antibody) blood sample should be taken as close to the onset of the event as possible, at the resolution of the event, and 28 days following the event.

### 6.2 Nivolumab

Nivolumab, supplied by BMS.

Preparation for Administration

Visually inspect drug product solution for particulate matter and discoloration prior to administration. Nivolumab is a clear to opalescent, colorless to pale-yellow solution. Discard the vial if the solution is cloudy, discolored, or contains extraneous particulate matter other than a few translucent-to-white, proteinaceous particles. Do not shake the vial.

• Withdraw the required volume of nivolumab and transfer into an intravenous container.

• Dilute nivolumab with either 0.9% Sodium Chloride Injection, USP or 5% Dextrose Injection, USP to prepare an infusion with a final concentration ranging from 1 mg/mL to 10 mg/mL.

• Mix diluted solution by gentle inversion. Do not shake.

• Discard partially used vials or empty vials of nivolumab.

Storage of Infusion

The product does not contain a preservative. After preparation, store the nivolumab infusion either:

• at room temperature for no more than 8 hours from the time of preparation. This includes room temperature storage of the infusion in the IV container and time for administration of the infusion or

• under refrigeration at 2°C to 8°C (36°F to 46°F) for no more than 24 hours from the time of infusion preparation. Do not freeze.

Administration

Administer the infusion over 30 minutes (± 5 minutes) through an intravenous line containing a sterile, non-pyrogenic, low protein binding in-line filter (pore size of 0.2 micrometer to 1.2 micrometer). Do not coadminister other drugs through the same intravenous line. Flush the intravenous line at end of infusion. *VX15/2503 should be administered prior to nivolumab. * Patients should remain 1 hour after all drugs are infused for monitoring.

Dosage forms

Injection: 100 mg/10 mL (10 mg/mL) solution in a single-dose vial.

## 6.3 Ipiliumumab

Ipilimumab, supplied by BMS.

Preparation for administration

Do not shake product.

• Inspect parenteral drug products visually for particulate matter and discoloration prior to administration. Discard vial if solution is cloudy, there is pronounced discoloration (solution may have pale-yellow color), or there is foreign particulate matter other than translucent-to-white, amorphous particles.

• Allow the vials to stand at room temperature for approximately 5 minutes prior to preparation of infusion.

• Withdraw the required volume of ipilimumab and transfer into an intravenous bag.

• Dilute with 0.9% Sodium Chloride Injection, USP or 5% Dextrose Injection, USP to prepare a diluted solution with a final concentration ranging from 1 mg/mL to 2 mg/mL. Mix diluted solution by gentle inversion.

Storage of infusion

• Store the diluted solution for no more than 24 hours under refrigeration (2°C to 8°C, 36°F to 46°F) or at room temperature (20°C to 25°C, 68°F to 77°F).

• Discard partially used vials or empty vials of ipilimumab.

Administration Instructions

• Do not mix ipilimumab with, or administer as an infusion with, other medicinal products.

• Flush the intravenous line with 0.9% Sodium Chloride Injection, USP or 5% Dextrose Injection, USP after each dose.

• Administer diluted solution over 30 minutes (± 5 minutes) through an intravenous line containing a sterile, non-pyrogenic, low-protein-binding in-line filter.

*VX15/2503 should be administered prior to ipilimumab.

*For patients receiving both nivolumab and ipilimumab no wait time is needed between the end of nivolumab and the start of ipilimumab.

*Patients should remain 1 hour after all drugs are infused for monitoring.

Dosage forms
Injection: 50 mg/10 mL (5 mg/mL)
Injection: 200 mg/40 mL (5 mg/mL)

### 6.4 Treatment Compliance

VX15/2503 will be administered via IV infusion at the study site by trained staff. Treatment compliance will be monitored through documentation of any infusion-related issues in the subject's source documents and case report form.

### 6.5 Drug Accountability

The FDA requires accounting for the disposition of all investigational drugs received by each clinical site. Information on drug disposition required by Federal law consists of the date received, date administered, quantity administered, and the subject to whom the drug was administered. The Investigator is responsible for the accounting for all used and unused study drug. After the inventory is reconciled by the Investigational Drug Service, used drug may be disposed of on-site following all local and federal regulations.

The site will use a standard form to document study drug disposition. This form must be maintained either in the pharmacy or in another appropriate area. Each time a dose is prepared for a subject, the following information should be recorded: the date of dispensation, subject's initials, the subject study number, the total dose prepared, the number of vials used, the lot number, and the initials of the person dispensing the study drug. At the termination of the study, a copy of the Drug Disposition Forms must be provided to the Sponsor. In addition, a copy of all Drug Disposition Forms must be retained in the Investigator's Study Files.

### 6.6 Contraindications, Precautions and Warnings

Subjects with a history of severe allergic reactions to excipients should not participate in this clinical trial.

### 6.7 Concomitant Treatment

Medications taken within 14 days prior to Day 1 and during the study will be collected on the subject's case report form. The following are the supportive care guidelines for the study:

- No other antineoplastic agents will be permitted during this study
- No treatment with chronic immunosuppressants (e.g., cyclosporine following transplantation or systemic steroids for treatment of autoimmune disease) will be permitted during this study.
- The use of erythropoietin or other specific red blood cell growth factors and red blood cell transfusions will be permitted as clinically indicated during the study
- Coumadin: Patients taking coumadin-derived anticoagulants while participating in this study should be monitored regularly for alteration of anticoagulation parameters (PT/INR). Subcutaneous heparin or fractionated heparin products are permitted. A daily dose of aspirin, 81 mg or 325 mg for cardiovascular prophylaxis, is permitted.
- Other concomitant medications may be given as clinically indicated

The above information is precautionary only. Investigators are encouraged to discuss the use of individual medications with the Sponsor on a case-by-case basis.

# 7 CORRELATIVE ASSESSMENTS

## 7.1 Pre-treatment Tumor Samples (Biopsy and/or Archived Tumor Specimens)

Pre-treatment biopsies or archived tumor specimens from the original diagnostic biopsy will be collected. This is anticipated to be in the form of paraffin blocks or unstained slides. If possible, at least 15 unstained slides with mounted tissue will be requested.

## 7.2 Post- Treatment Tumor Biopsy

Post-treatment tumor samples will be collected on the day of surgery (day 35-49). Specimens from surgical resection will be collected under the guidance of the collaborating pathologist and tissue procurement staff at the time of surgery to capture tissue in excess of that needed for clinical staging and diagnostic purposes.

1) Formalin-fixed paraffin block with one cut H&E stained slide
2) Liquid nitrogen frozen tissue in cryo-preservation vials
3) RNAlater for RNA stabilization and tissue storage

All collected tissues are stabilized and stored in -80°C freezers (RNAlater stabilized, short term storage) or the vapor phase of a liquid nitrogen freezer (long term storage) in single use aliquots. All FFPE tissue blocks are stored in a climate-controlled storage room that is temperature (less than 27ºC) and humidity controlled.

<u>Biomarker analysis</u>

These tissues will be prioritized and divided as follows:

1. Tumor specimens will be fixed in 10% formalin, embedded in paraffin and biomarkers related to activated pancreatic stroma, immune infiltrates will be assessed via immunohistochemical analysis.
   Finally, formalin-fixed paraffin embedded tissues can be utilized to assess T cell receptor diversity via immunoseq assay (Adaptive Biotechnologies) if meaningful phenotypic shifts are observed following therapy.

2. Freshly isolated tissue will be dissociated using enzymatic degradation and the GentleMACS system. Resulting homogenates will be assessed and multi-parameter flow cytometry will be used to quantify individual immune subsets present within the tumor microenvironment.3. When sufficient quantity permits, a section of fresh tumor samples will be split and snap frozen either (1) alone for future genomic analysis or (2) in RNALater solution to facilitate future high throughput analysis of gene expression profile.

<u>Handling instructions</u>

After appropriate processing, the samples will be sent to:

Gregory Lesinski PhD
Winship Cancer Institute
Building C, Room C3090
1365 Clifton Road NE
Atlanta, GA 30322

Tel: 404-778-3072
Email: Gregory.B.Lesinski@emory.edu

Dr. Lesinski's lab should be alerted before specimens are collected by calling:
(404) 778-6150.

Methods and materials per Lab Manual (APPENDIX III)

### 7.3 Blood Sample Analysis

Peripheral blood samples will be collected prior to initiation of study therapy, on day 1 and 21, after therapy on day 1, 7 days prior to the day of surgery (day 35-49). The peripheral blood mononuclear cells will be isolated from these samples and stored in -80°C freezers until analysis.

Blood will be evaluated for:

- The impact of treatment on the circulating percentage of immune suppressor subsets that may serve as predictors of response to immune checkpoint inhibition. Examples include: myeloid derived suppressor cells, T regulatory cells, and IL-10 producing B regulatory cells.
- The impact of treatment on phenotypic shifts and function in T lymphocyte subsets. In particular, Th1/Th2/Th17 or Th subsets with characteristics consistent with naïve, central and effector memory phenotypes. Phenotypic data will be complemented by *ex vivo* analysis of function (intracellular cytokine staining, proliferation) in aliquots of freshly isolated T cells.

Methods and materials per Lab Manual (APPENDIX II)

## 8 SAFETY ASSESSMENTS

### 8.1 Clinical Safety Assessments

Clinical safety assessments will include the number, severity, and treatment association of AEs (serious and non-serious), vital sign measurements, and concomitant medications.

### 8.2 Laboratory Safety Assessments

Clinical laboratory safety assessments will include changes from baseline in serum chemistry, hematology, coagulation and urinalysis.

### 8.3 Adverse Events/Experiences

#### 8.3.1 Definitions

**Adverse Events**

An AE is defined as any untoward medical occurrence in a subject administered a pharmaceutical product, which does not have to have a causal relationship with the treatment.

An AE can therefore be any unfavorable and unintended sign (including an abnormal laboratory finding, for example), symptom, or disease temporally associated with the use of a medicinal product, whether or not considered related to the medicinal product.

**Adverse Drug Reaction**

An Adverse Drug Reaction (ADR) is defined as all noxious and unintended responses to a medicinal product, related to any dose.

A causal relationship between the medicinal product and an AE is at least a reasonable possibility, e.g. the relationship cannot be ruled out.

**Unexpected AE**

An unexpected AE is one that is not identified in nature, severity or frequency as described in the current Investigator's Brochure or package insert.

**Unexpected Adverse Drug Reaction**

An unexpected adverse drug reaction (UADR) is defined as an adverse reaction, the nature or severity of which is not consistent with the applicable product information (e.g. Investigator's Brochure). ADRs which are more specific or more severe than described in the Investigator's Brochure should also be considered unexpected.

Any clinically significant abnormality determined by the investigator found on an ECG, laboratory test, or physical examination will also be considered an AE if it occurs or worsens in severity or frequency after the first dose of study drug, (this includes worsening of signs, symptoms, or diagnoses present prior to the first dose of study drug) whether or not it is considered related to study drug. All AEs will be followed until they stabilize or resolve.

A **serious adverse event (SAE)** is any AE occurring at any dose that meets one or more of the following criterion.

- Results in death
- Is life-threatening event - A **life-threatening AE** is any AE that places the subject at immediate risk of death from the event as it occurred; a life-threatening event does not include an event that, had it occurred in a more severe form, might have caused death, but as it actually occurred, did not create an immediate risk of death.
- Requires inpatient hospitalization or prolongation of an existing hospitalization - **Hospitalization** is defined as an admission to hospital. In this study, any pre-planned hospitalization for an elective procedure that is unrelated to participation in this study will not be considered an SAE.
- Results in a persistent or significant disability or incapacity **- Disability** is defined as a substantial disruption in a person's ability to conduct normal life functions.
- Results in a congenital anomaly or birth defect
- Any event that does not meet the above criteria, but that in the judgment of the investigator jeopardizes the subject, will be considered for reporting on a serious AE form, when based on appropriate medical judgment. The event can require medical or surgical intervention to prevent one of the outcomes listed in the definition of "SAE."

**Relationship** of an AE to the study drug is as follows:

- **Definitely Related**: An AE that follows a reasonable temporal sequence from administration of the study drug; follows a known response pattern to the drug; and, when appropriate to the protocol, is confirmed by improvement on stopping the drug (dechallenge) and reappearance of the reaction after repeat exposure (rechallenge); and cannot be reasonably explained by the known characteristics of the subject's clinical state or by other therapies.

- **Probably Related**: The event follows a reasonable temporal sequence from the time of drug administration, and follows a known response pattern to the study drug, and cannot be reasonably explained by other factors such as the subject's clinical condition, therapeutic interventions, or concomitant drugs administered to the subject. .
- **Possibly Related**: The event follows a reasonable temporal sequence from the time of drug administration, and/or follows a known response pattern to the study drug, but could have been produced by other factors such as the subject's clinical condition, therapeutic interventions, or concomitant drugs administered to the subject.
- **Unlikely to be Related**: The event is most likely produced by other factors such as the subject's clinical condition, therapeutic interventions, or concomitant drugs administered to the subject, and does not follow a known response pattern to the study drug.
- **Not Related**: An AE for which sufficient information exists to indicate that the etiology is unrelated to the study drug. Another etiology must be specified.

**Definitions of the severity of AEs** are listed in the NCI CTCAE version 4.0 (https://evs.nci.nih.gov/ftp1/CTCAE/CTCAE_4.03_2010-06-14_QuickReference_8.5x11.pdf). For the AEs not listed in the CTCAE scale, the following definitions should be used:

- **Mild (Grade 1)**: The AE is noticeable to the subject but does not disrupt normal daily activities; it does not require discontinuation of the study drug, but may require additional therapy.
- **Moderate (Grade 2)**: The AE interferes with the subject's daily activities; it does not require discontinuation of the study drug but may require additional therapy.
- **Severe (Grade 3)**: The AE is intolerable and disrupts normal daily activities, may require additional therapy or hospitalization, and/or discontinuation of the study drug.
- **Life-threatening (Grade 4)**: The AE exposes the subject to risk of death at the time of the event; it does not refer to an event that may have caused death if the event was more severe.
- **Death related to AE (Grade 5):** An event that results in death is considered a grade 5 AE, and by definition, is an SAE.

Any event with a fatal outcome is considered to be serious, and requires completion of a SAE form.

Note: Cohort E patients are being enrolled as a control sample for biospecimen collection purposes only. Due to the fact that Cohort E patients will not be receiving any drugs prior to their Standard of Care surgical procedures, no adverse event data will be collected for this cohort of subjects.

# 9 REPORTABLE EVENTS/INFORMATION

## 9.1 Reporting of Serious Adverse Events Associated with VX15/2503

All serious adverse events that are considered related or **unrelated** to VX15/2503

treatment must be recorded on a MedWatch 3500 Form and emailed to pv@vaccinex.com within 1 business day to:

Vaccinex : John Leonard, jleonard@vaccinex.com

Telephone number: 760 815 5969

Fax number: 585 271 2765

MedWatch 3500 Reporting Guidelines:

*Note: MedWatch 3500 forms and other information related to MedWatch reporting are available at http://www.fda.gov/medwatch/index.html.*

In addition to completing appropriate patient demographic, suspect medication and reporter information, the report should include the following information within the Event Description of the MedWatch 3500 form:

- Protocol description (and number, if assigned);
- Description of event, severity, treatment, causality and outcome if known;
- Supportive laboratory results and diagnostics; and
- Investigator's assessment of the relationship of the adverse event to each investigational product and suspect medication

## 9.2 Reporting of Serious Adverse Events Associated with Nivolumab/ipilimumab or study drug

Report SAEs to BMS, related or not **related** to study drug, within 24 hours\1 business day of becoming aware of the event.

SAE Emailing Address: Worldwide.Safety@BMS.com

SAE Facsimile Number: 1-609-818-3804

All SAEs must be collected that occur during the screening period and within 100 days of the last dose of study treatment.

### 9.3 Reporting Requirements for IND holders

For Investigator-sponsored IND studies, reporting requirements for the FDA apply in accordance with the guidance set forth in 21 CFR, Part 312.32. Events meeting the following criteria need to be submitted to the FDA as Expedited IND Safety Reports.

<u>7 Calendar-Day Telephone or Fax Report</u>

The Sponsor-Investigator is required to notify the FDA of a fatal or life-threatening adverse event that is unexpected and assessed by the investigator to be possibly related to the use of *VX15/2503*. An unexpected adverse event is one that is not already described in the most recent Guidance for Investigator section of the Investigator's Brochure. Such reports are to be telephoned or faxed to the FDA and Vaccinex within 7 calendar days of the first learning of the event.

<u>15 Calendar-Day Written Report</u>

The Sponsor-Investigator is also required to notify the FDA and all participating investigators, in a written IND Safety Report, of any serious unexpected adverse event that is considered reasonably or possibly related to the use of VX15/2503.

Written IND Safety Reports with analysis of similar events are to be submitted to the FDA, Vaccinex, and all participating investigators within 15 calendar days of first learning of the event. The FDA prefers these reports on a MedWatch 3500 Form but alternative formats (eg, summary letter) are acceptable. The IND sponsor will also make an assessment of whether the event constitutes an unanticipated problem posing risks to subjects or others (UP). This assessment will be provided to the Emory University IRB, which, in turn will make a final determination. If the Emory IRB determines an event is a UP it will notify the appropriate regulatory agencies and institutional officials.

FDA Fax number of IND Safety Reports: 1-(800)-FDA-1078

All written IND Safety Reports submitted to the FDA by the Sponsor-Investigator must also be faxed to:

Vaccinex Drug Safety

Fax number: 585 271 2765

For questions related to safety reporting, contact Vaccinex Drug Safety at.

Vaccinex : John Leonard, jleonard@vaccinex.com

Telephone number: 760 815 5969

Fax number: 585 271 2765

### 9.4 Pregnancy

All **pregnancies** occurring during this study are reported in the same timeframe as SAEs. If a pregnancy is reported during the study, the continued use of the study drug must be immediately evaluated. A repeat pregnancy test should be completed as soon as possible. When possible, all reports of pregnancy must be followed for information regarding the course of the pregnancy and delivery, as well as the condition of the newborn. Follow-up information concerning the outcome of the pregnancy should be provided by the Investigator to the study medical monitor/ Drug Safety department in a timely manner and regardless of whether the subject has discontinued participation in the study. When the newborn is healthy, additional follow-up is not needed. This includes both female patients enrolled on the study and pregnancy of a female partner of a male patient enrolled on the study. Any pregnancy that occurs in a female partner of a male study participant should be reported to BMS. Information on this pregnancy will be collected on the Pregnancy Surveillance Form. In order for Sponsor or designee to collect any pregnancy surveillance information from the female partner, the female partner must sign an informed consent form for disclosure of this information

## 10 Data Quality Assurance

Winship Cancer Institute's Clinical Trials Office will perform the quality assurance and quality control checks on this clinical trial. Before enrollment of any subject in this study, CTO personnel and the Investigator will review the protocol; the Investigator's Brochure; the CRFs/electronic CRFs and instructions for completion; the informed consent process; and the procedure for reporting AEs and SAEs.

## 11 STATISTICAL CONSIDERATIONS

### 11.1 Determination of Sample Size

This is an integrated pilot biomarker study design in patients with resectable metastatic melanoma. Each treatment cohort will have 8 evaluable patients enrolled; the control cohort (no treatment) will have 6 evaluable patients enrolled. 45 patients will be enrolled on the trial; up to 42 patients will undergo treatment, thus allowing for screen failures or consent withdrawals as well as potential for unevaluable patients receiving treatment. There will be no dose-escalation or dose-modifications in the trial. Stopping Rules: Not Applicable

## 11.2 Statistical and Analytical Plans

### 11.2.1 General Analysis

Summary statistics will be presented for all safety and biomarker parameter analyses. The purpose of these analyses is hypothesis generating. Various exploratory statistical tests may be applied to data generated from this trial to generate hypotheses to be tested in subsequent trials. In general, all descriptive statistical analyses will be performed using the most recently released and available SAS statistical software, unless otherwise noted. For categorical variables, the number and percent of each category within a parameter will be calculated. For continuous variables, the sample size (n), mean, median, standard deviation, and coefficient of variation, as well as the minimum and maximum values, will be presented. Missing data will not be imputed unless otherwise stated. There will be a detailed description of subject disposition; subject demographics and baseline characteristics will be summarized. Toxicities will be presented as worst toxicity per patient and will be reported as percent toxicity.

### 11.2.2 Biomarker Analysis

The two-sample t-test will be used to compare the change in expression of biomarker change after treatment between each experimental group (Cohort A, B, C, and D) and the control group (cohort E), respectively. A Bonferroni correction will be used to control type I error at 5% across 4 comparisons. A mixed model with random effect for intercept and group will be used to compare the overall change in expression of biomarker during the whole experiment among the different groups. If there is a significant difference overall, pairwise comparisons of overall change in expression of biomarker will be further conducted for each experimental group compared with the control group, respectively. ANOVA will be conducted to compare the change in biomarkers and other outcomes at the treatment endpoint from baseline between the different groups. Pairwise group differences will be further tested by using Tukey's method when the overall difference is significant at a level of 0.05.

### 11.2.3. Analysis of Response

For patients to be considered evaluable for efficacy they must have completed the treatment and have a baseline tumor assessment. Response rate (ORR or CBR) will be calculated as proportion (Responders/Total patients) along with 95% confidence intervals using the Clopper-Pearson method. Chi-square test or Fisher's exact test will be used to compare the efficacy in term of response rate between the different groups stratified by dose level or other factors, respectively. Logistics regression model will be further employed to test the adjusted effect of dosage on the response rate after adjusting for other clinical factors and demographic factors.

### 11.2.4. Analysis of Other Endpoints

For progression free survival, progression or death from any cause will be defined as the event. Patients will be censored at time of last follow-up. For overall survival, death from any cause will be defined as the event. Patients will be censored at time of last follow-up. Overall survival (OS) and progression free survival (PFS) rates of two patient groups stratified clinicopathologic factors will be estimated with the Kaplan-Meier method and compared between different groups using the log-rank test, respectively. The PFS and OS of each patient group at specific time points, such as 6 moths, 1 year, 3 year, and 5 year, etc. will be also estimated alone with 95% CI. Cox proportional hazards models will be further used in the multivariable analyses to assess adjusted effect of dose levels on the patients' OS and PFS after adjusting for other factors. Interaction terms between these

factors will also be tested for statistical significance. The proportional hazards assumption will be evaluated graphically and analytically with regression diagnostics. Violations of the proportional hazards assumptions will be addressed by use of time-dependent covariates or extended Cox regression models.

#### 11.2.5 Power and sample size calculation

Assuming a CV of 50%, n=8 patients the in treatment groups and 6 patients in the control group can achieve at least 80% power to detect a 3-fold difference in change of expression by treatment between the pairwise groups ($\alpha$=0.0125, 2-sided).

### 11.3 Analysis Sets

All subjects who receive any amount of study drug will be included in the evaluation of safety, except for patients who take less than 80% of their prescribed doses of VX15/2503 since they will be considered unevaluable for the primary endpoint of toxicity.

### 11.4 Subject Disposition and Baseline Characteristics

The number and percentage of subjects who enrolled, were treated, and who discontinued will be tabulated. The reasons for treatment and study discontinuation will be presented. Demographic and other baseline characteristics will be summarized using descriptive statistics or counts and percentages, as appropriate.

### 11.5 Safety Analysis

##### 11.5.1.1 Adverse Events

Adverse events will be classified using MedDRA System Organ Classes and Preferred Terms. Furthermore, SAEs, AEs with a severity grade of 3 or above using NCI CTCAE version 4.0, AEs deemed related to study drug, AEs leading to discontinuation of study drug, and AEs leading to death will also be summarized in preferred term by system organ class and listed on an individual subject basis.

##### 11.5.1.2 Laboratory Data

Descriptive statistics for worst grade of each laboratory parameter by the NCI CTCAE scale version4.0 at baseline and follow-up will be presented along with change from Baseline. Additionally, laboratory values ≥ Grade 3 severity will be tabulated and listed on an individual subject basis.

##### 11.5.1.3 Dose Modifications and Reasons

The number of subjects with skipped doses and dose delays will be summarized.

## 12 INVESTIGATOR REGULATORY OBLIGATIONS

### 12.1 IRB/IEC Approval

The protocol and informed consent document must have the approval of a properly constituted committee (IRB or IEC) responsible for approving clinical trials. The signed IRB/IEC approval letter must identify the documents approved (i.e., list the Investigator's name, the protocol number and title, the date of the protocol, and the date of approval of the protocol and the informed consent document). Any advertisements used to recruit subjects must also be reviewed by the IRB/IEC.

### 12.2 Prestudy Documentation

The Investigator must provide Vaccinex with the following documents prior to the enrollment of any subjects:

- Copy of the IRB/IEC approval letter for protocol, informed consent, Investigator and site
- Signed and dated current curricula vitae of the investigator
- Copy of approved informed consent document
- Copy of the FDA letter and IND receipt and number assignment
- Signed Clinical Trial Agreement

### 12.3 Informed Consent

The informed consent document must be reviewed and approved by the investigative site IRB/IEC prior to the initiation of the study.

Prior to the start of any protocol-specific evaluations or screening procedures, the Investigator (or designated staff) will explain the nature of the study and its risks and benefits to the subject (or the subject's legal representative). Each subject will receive an informed consent document with subject information. Subjects should be given ample time to read the information and the opportunity to ask questions. Informed consent must be obtained from each subject prior to performing any protocol-specific evaluations. One copy of the signed informed consent document will be given to the subject, and another will be retained by the Investigator.

### 12.4 Protocol Adherence

By signing the Form FDA 1572, the Investigator agrees to conduct the study according to the protocol and the FDA regulations set forth in 21 CFR Parts 50, 54, 56, and 312.

### 12.5 Permission to Review Subjects' Source Records

The Investigator agrees that Vaccinex, its employees or agents, and the FDA and parallel international authorities will have the right from time to time, both during and after this trial, to audit and review pertinent medical records related to the clinical trial. By signing the informed consent, each subject who participates in the trial permits the release of his medical records as necessary for inspection by authorized personnel from Vaccinex and/or the FDA and parallel international authorities. Subjects will not be identified by name, and confidentiality of information in medical records will be preserved by Vaccinex, to the extent required by law.

### 12.6 Protocol Amendments

All amendments to the study protocol must be submitted to the IRB/IEC for approval according to institution standards. The approval letter, signed by the IRB/IEC Chairperson or by designated reviewer/qualified IRB staff, must refer specifically to the Investigator, the protocol number and protocol title, the protocol amendment number, and the date of the protocol amendment. A copy of the approval letter and revised informed consent document (if appropriate) must be sent to Vaccinex. A protocol amendment may be implemented only after it has been approved by the IRB/IEC and has been submitted to the FDA. In the case of a protocol change intended to eliminate an apparent immediate hazard to subjects, the change may be implemented immediately, but the change must then be documented in a protocol amendment and approved as described above.

### 12.7 Change in Investigator

If, during the conduct of the study, any Investigator retires, relocates, or withdraws from an investigation, the responsibility for conduct of the study may be transferred to another appropriately qualified Investigator at the investigative site. Vaccinex must be notified and agree to the change. An updated signed and dated CV must be submitted to Vaccinex. The sponsor-investigator will also submit an IND Amendment to the FDA.

### 12.8 Retention of Study Documents

All CRFs, as well as supporting documentation, administrative records, and drug inventory, must be retained by the Investigator for a minimum of 2 years after the last approval of a marketing application in an International Conference on Harmonisation (ICH) region and until there are no pending or contemplated marketing applications in an ICH region or at least 2 years have elapsed since the formal discontinuation of clinical development of the investigational product. At the end of the 2-year period, Vaccinex must be consulted prior to the destruction of study documents. OnCore will be used to store patient and study data.

If any investigator retires, relocates, or withdraws from an investigation after the conduct of the study, the responsibility for maintenance of study records may be transferred to another person (Vaccinex, IRB/IEC, other investigators). Vaccinex must be notified and agree to the change.

### 12.9 Data Safety and Monitoring

- This study will be monitored by the Winship Cancer Institute Data and Safety Monitoring Committee. As an investigator-initiated study, initial study monitoring will occur within 6 months from when the first subject accrued to the study. At that time, two of the first 5 patients enrolled to the study will be monitored. Thereafter, subsequent monitoring will occur in six-month i ntervals if any subjects were accrued. The population continuing to receive intervention will be monitored on a study-by-study basis.
  - At minimum, 10% of subjects accrued since previous monitoring will be reviewed.
  - An additional subject (or subjects) may be selected based on previously noted monitoring deficiencies or at DSMC discretion.

Continued monitoring will occur in six-month intervals for the population continuing to receive intervention on a study-by-study basis. In addition, the Gastrointestinal Working Group will review the status of every patient enrolled to the study in the fortnightly meetings. Reporting requirements will be in accordance with the policies of the Institutional Review Board of Emory University.

## 13. APPENDIX I: ECOG PERFORMANCE STATUS

| Grade | ECOG |
|---|---|
| 0 | Fully active, able to carry on all predisease performance without restriction |
| 1 | Restricted in physically strenuous activity but ambulatory and able to carry out work of a light or sedentary nature, e.g., light house work, office work |
| 2 | Ambulatory and capable of all self-care but unable to carry out any work activities. Up and about more than 50% of waking hours |
| 3 | Capable of only limited self-care, confined to bed or chair more than 50% of waking hours |
| 4 | Completely disabled. Cannot carry on any self-care. Totally confined to bed or chair |
| 5 | Dead |

## 14 APPENDIX II: LAB MANUAL

### LABORATORY MANUAL

**Clinical Study:** Phase I integrated biomarker trial of VX15/2503 in combination with ipilimumab or nivolumab in patients with resectable metastatic melanoma.

**Clinical Trial PI:** Michael Lowe, MD, MA

**Correlative Studies PI:** Gregory B. Lesinski, PhD, MPH; Brian Olson, PhD

**Correlative Study Site:** Lesinski Laboratory, Emory University

**Co-Investigators**
Ragini Kudchadkar, MD
Benjamin Stoff, MD
David Lawson, MD
Melinda Yushak, MD
Keith Delman, MD
Maria Russell, MD

**Biostatistician**
Subir Goyal Ph.D
**Study Coordinators**
Agnes Harutyunyan
**Laboratory Address:**
Lesinski Laboratory
Suite C3038, Bay 34
1365-C Clifton Rd. NE
Winship Cancer Institute of Emory University
Atlanta, GA 30322

**Contact Information:**
Matthew Farren, Ph.D. 404-778-8230
Michael Lowe 404-778-3738

**<u>Table of Contents:</u>**


- **A. Overview of Correlative Studies**
- **B. Collection and Handling of Specimens**
- **C. Immunohistochemical analysis of tumor tissue**
- **D. Analysis of T cell receptor diversity and clonality**
- **E. Flow cytometric analysis of tumor biopsy tissue**
- **F. Lesinski Lab SOP (Tumor Homogenization and FACS analysis)**
- **G. Excess tumor tissue**
- **H. Blood Specimen Processing**
- **I. Cytokine or Soluble Factor Analyses**
- **J. Immune suppressor cell analyses**
- **K. Analysis of T cell phenotype and function**

***A. Overview of Correlative Studies:***
The primary objective for our laboratory correlative studies will be to determine whether treatment with VX15/2503 in combination with immune checkpoint inhibitors (ipilimumab or nivolumab) modulates the phenotypic properties within the tumor microenvironment. The particular biomarkers selected were derived from pre-clinical studies in murine tumor models that demonstrate SEMA4D blockade can synergize with checkpoint inhibition to recruit immune cells into the tumor microenvironment. Based on independent studies in murine models of liver inflammation, it is also possible that SEMA4D blockade will break down the fibrotic stroma that may limit access to the tumor. Patient biopsies and peripheral blood samples obtained during the course of this study will be processed utilizing the protocols listed below.

***B. Collection and Handling of Specimens:***
Collection and handling of tumor biopsy and resection specimens will proceed as described in Section 3.2.1 and 3.2.2 of the clinical protocol.

***C. Immunohistochemical analysis of tumor tissue:***
Paired tumor biopsies obtained at baseline (when available) and tumor resection specimens will be used to assess the effect of treatment arm on changes in the TME. This analysis will be performed on paired samples in n=16 melanoma patients enrolled in the trial. Immunohistochemical (IHC) and immunofluorescence (IF) analysis of patient samples will proceed in the Winship Core Pathology Laboratory, or the Lesinski Laboratory, respectively, using standardized protocols. The immune and stromal markers in **Table 1** will be the initial basis of investigation, while other markers can be assessed in an exploratory manner as data becomes available in the scientific literature. For example, this panel can also be expanded for other co-stimulatory or immune checkpoint molecules on cell subsets (i.e. CD8, Tim3, LAG3). De-identified tissue will be shipped to Vaccinex, Inc. for analysis of SEMA4D and Plexin-B1 via an optimized IHC assay. Tissues will be analyzed in the Integrated Cellular Imaging (ICI) Core Lab as described in section 3.2.4 of the clinical protocol.

**Table I. Planned Immunohistochemical (IHC) and Immunofluorescence (IF) analysis**

| IHC Stains | IF Stains |
|---|---|
| CD4 | CD4+FoxP3+ |
| CD8 | CD8+Ki67+ |
| CD11c | CD33+S100+ |
| CD19 | CD4+IL-17+ |
| CD68 | aSMA+FAP+ |
| CD163 | aSMA+IL-6+ |
| PD-L1 | FAP+IL-6+ |
| PD-L1 | PDGRFβ+IL-6+ |
| Sirius Red | |
| PD-L1 | |
| PD-L2 | |
| SEMA4D | |
| Plexin-B1 | |
| LAG3 | |

***D. Analysis of T cell receptor diversity and clonality.***
Formalin-fixed paraffin embedded tissues can be utilized to assess T cell receptor diversity and clonality via survey level immunoseq assay. For these studies, total DNA will be isolated from

FFPE tumor tissues and sent to Adaptive Biotechnologies, Inc. for Illumina sequencing of the TCRβ gene in each sample as previously described.[38] Data will be analyzed using the ImmunoSEQ Analyzer (Adaptive Biotechnologies, Inc.).

***E. Flow cytometric analysis of tumor biopsy tissue.***

When fresh tissue from initial biopsy is available (and in all surgical specimens at the study endpoint), IHC/IF will be complemented by a detailed flow-based phenotyping of biopsy tissue to gather more information on immune subsets and to determine if tumor infiltrating T cells are saturated with VX15/2503. Our lab has optimized a protocol to simultaneously analyze multiple immune subsets, and obtain quantitative data that correlates with IHC. For these studies, freshly isolated tissue will be dissociated using enzymatic degradation and the GentleMACS system per manufacturer's protocol with modifications (see Lesinski Laboratory Standard Operation Procedure Below). Resulting homogenates will be assessed and multi-parameter flow cytometry as described above to quantify individual immune subsets present within the tumor microenvironment. A basic flow cytometry panel to phenotype individual cell subsets co-labeled with ZombieViolet Dye and anti-CD45-APC/Cy7 will be utilized to date upon live/dead lymphocytes from the resulting homogenates.

***F. Lesinski Lab SOP (Tumor Homogenization and FACS analysis):***

**Protocol for human tumor homogenization and subsequent FACS of infiltrating immune cells**

Note, this protocol as written is optimized for "tougher", more fibrotic tumors such as pancreatic ductal adenocarcinoma (PDAC) tumors. Protocol may need modified for the homogenization of "soft" (e.g. melanoma, colorectal) or "medium" (e.g. lung, prostate) tumors.

**Mixed Reagents**

FACS Buffer: PBS + 5% heat-inactivated fetal bovine serum (FBS)
1% Paraformaldehyde: Paraformaldehyde diluted in PBS
Working concentration ZombieViolet dye: lyophilized ZombieViolet from Biolegend diluted in DMSO (diluted at 1 µL per "Test"; e.g. a 100 test vial of ZombieViolet is diluted to working concentration with 100 µL DMSO)

**Dissociation Procedure**

*Preliminary*

1. Obtain tumor specimen(s) through appropriate IRB and trial-protocol approved channels. This will vary from trial to trial, and is not specified in this general purpose protocol.
2. Prepare enzyme mix: add 4.7 mL RPMI 1640 (without supplemental FBS, anti/anti-, or L-Glut), 200 µL Enzyme H, 100 µL Enzyme R, and 25 µL Enzyme A into a gentleMACS "C Tube". If digesting multiple tumors, prepare one tube (with this buffer/enzyme mix) for EACH tumor sample.
3. If using some of the tumor for IHC, cut off that bit now and place it in the appropriate place (e.g. formalin, OCT media)
4. Weigh tumor(s), record mass in appropriate notebook.
5. In biosafety cabinet, cut tumor into small pieces (2-4 mm x 2-4 mm) using small scissors, scalpels, or razor blades, following appropriate safe working conditions.

*Dissociation of "tough" tumors (e.g. pancreatic ductal carcinoma (PDAC) tumors)*

6. Put tumor pieces into "C tube" containing buffer/enzyme mix prepared in step 2
7. Tightly close tube, attach it upside down into one of the two sleeves on the gentleMACS dissociator (either sleeve may be used). Make sure tumor samples are near the rotor/stator

and not adhered to the upper walls of the tube. Ensure tube is seated properly. You should hear and feel a "click" when the tube is properly attached.

8. Run gentleMACS program "h_tumor_01".
9. Detach tube. Incubate for 30 minutes at 37º C with continuous rotation. If tube rotator is not available, manually rotate/agitate tube every ~5 minutes.
10. Attach tube to gentleMACS dissociator, run program "h_tumor_01" (second time doing this).
11. Detach tube. Incubate for 30 minutes at 37º C with continuous rotation. If tube rotator is not available, manually rotate/agitate tube every ~5 minutes.
12. Attach tube to gentleMACS dissociator, run program "h_tumor_01" (third time doing this).
13. Using sterile RPMI 1640, resuspend sample/wash out of C tube and cap, strain through a 70 µm filter and wash with ~20ml media.
14. Should have a (mostly) single cell suspension free of chunks of tissue. Centrifuge ~300g (1500 RPM on Eppendorf 5810R centrifuge) for 5 minutes. Resuspension buffer/media and volume will depend on downstream application.

*Dissociation of "medium" tumors (e.g. lung, prostate tumors)*

5. Put tumor pieces into "C tube" containing buffer/enzyme mix prepared in step 2
6. Tightly close tube, attach it upside down into one of the two sleeves on the gentleMACS dissociator (either sleeve may be used). Make sure tumor samples are near the rotor/stator and not adhered to the upper walls of the tube. Ensure tube is seated properly. You should hear and feel a "click" when the tube is properly attached.
7. Run gentleMACS program "h_tumor_01".
8. Detach tube. Incubate for 30 minutes at 37º C with continuous rotation. If tube rotator is not available, manually rotate/agitate tube every ~5 minutes.
9. Attach tube to gentleMACS dissociator, run program "h_tumor_02".
10. Detach tube. Incubate for 30 minutes at 37º C with continuous rotation. If tube rotator is not available, manually rotate/agitate tube every ~5 minutes.
11. Attach tube to gentleMACS dissociator, run program "h_tumor_02".
12. Using sterile RPMI 1640, resuspend sample/wash out of C tube and cap, strain through a 70 µm filter and wash with ~20ml media.
13. Should have a (mostly) single cell suspension free of chunks of tissue. Centrifuge ~300g (1500 RPM on Eppendorf 5810R centrifuge) for 5 minutes. Resuspension buffer/media and volume will depend on downstream application.

*Dissociation of "soft" tumors (e.g. melanoma, colon)*

5. Put tumor pieces into "C tube" containing buffer/enzyme mix prepared in step 2
6. Tightly close tube, attach it upside down into one of the two sleeves on the gentleMACS dissociator (either sleeve may be used). Make sure tumor samples are near the rotor/stator and not adhered to the upper walls of the tube. Ensure tube is seated properly. You should hear and feel a "click" when the tube is properly attached.
7. Run gentleMACS program "h_tumor_01".
8. Detach tube. Incubate for 30 minutes at 37º C with continuous rotation. If tube rotator is not available, manually rotate/agitate tube every ~5 minutes.
9. Attach tube to gentleMACS dissociator, run program "h_tumor_02".
10. Detach tube. Incubate for 30 minutes at 37º C with continuous rotation. If tube rotator is not available, manually rotate/agitate tube every ~5 minutes.
11. Attach tube to gentleMACS dissociator, run program "h_tumor_03".
12. Using sterile RPMI 1640, resuspend sample/wash out of C tube and cap, strain through a 70 µm filter and wash with ~20ml media.

13. Should have a (mostly) single cell suspension free of chunks of tissue. Centrifuge ~300g (1500 RPM on Eppendorf 5810R centrifuge) for 5 minutes. Resuspension buffer/media and volume will depend on downstream application.

**Staining for flow cytometry**

Note: choice of antibodies (and therefore antibody targets) and fluorophores will be dependent on project/trial objectives and the flow cytometer that one is planning on using. Any antibodies or dyes listed here are in an example and may need modified depending specific experimental objectives and conditions.

*Staining with fixable live/dead dye*

Note: While not strictly necessary, staining with a live/dead dye, such as ZombieViolet (Biolegend), greatly reduces noise/background in subsequent flow cytometry.

1. Resuspend cells in PBS and count cells. Aliquot cells into FACS tubes for subsequent flow cytometry, including appropriate control tubes. Ideally, aliquot 1 x $10^6$ cells/tube.
2. Leave 1 tube COMPLETELY UNSTAINED, DO NOT ADD ZOMBIE-VIOLET TO THIS TUBE. VERY IMPORTANT.
3. Add 1 mL PBS/tube, centrifuge 5 minutes at 1500 RPM (in Eppendorf 5810R centrifuge, approximately 300g). After spin, decant supernatant and dab on fresh paper towel.
4. Add 1 mL PBS/tube, centrifuge 5 minutes at 1500 RPM (in Eppendorf 5810R centrifuge, approximately 300g). After spin, decant supernatant and dab on fresh paper towel.
5. Resuspend cells in 100uL PBS. Add 1 µL of working concentration Zombie Violet dye.
6. Vortex
7. Incubate 30 minutes at room temperature, in the dark.
8. Wash twice in FACS buffer

*Staining for cell surface markers*

1. Add 100 µL of appropriate antibody mastermix(s) to flow tube(s). (Example common master mixes used in lab are included at end)
2. Vortex.
3. Incubate 30-45 minutes at 4ºC in the dark/rack wrapped in aluminum foil.
4. Wash twice (add 1 ml FACS buffer, spin 5min at 1500 RPM, decant, dab dry on paper towel)
5. Vortex
6. Analyzing cells immediately, may go to flow cytometer now. Otherwise, fix cells/antibody by adding 300-1000 µL of 1% paraformaldehyde/PBS. Store at 4ºC wrapped in aluminum foil until analyzed on flow cytometer. Try to analyze within 7 days of initial staining.

**Example antibody panel from a recent immunophenotyping of tumor infiltrating lymphocytes**

Tube 1: Unstained
Tube 2: ZombieViolet stained only
Tube 3: (ZombieViolet) + anti-CD45-APC/Cy7
Tube 4: (ZombieViolet) + anti-CD45-APC/Cy7 + anti-CD19-APC
Tube 5: (ZombieViolet) + anti-CD45-APC/Cy7 + anti-CD4-APC
Tube 6: (ZombieViolet) + anti-CD45-APC/Cy7 + anti-CD8-APC
Tube 7 : (ZombieViolet) + anti-CD45-APC/Cy7+anti-CD4-FITC+Anti-CD25-APC+anti-FoxPe-PE

Tube 8: (ZombieViolet) + anti-CD45-APC/Cy7 + anti-CD11b-PE
Tube 9: (ZombieViolet) + anti-CD45-APC/Cy7 + anti-CD11b-PE + anti-CD33-PE/Cy7 + anti-CD14-FITC

Tubes 10-12 will be utilized to determine the % saturation of T cells by VX15/2503.
Tube 10 : Anti CD45 V500 / Anti CD3 Alexa488 + Isotype Anti human IgG4 Biotin + SA-APC (Isotype)
Tube 11 : Anti CD45 V500 / Anti CD3 Alexa488 + Anti human IgG4 Biotin + SA-APC (Bound Drug)
Tube 12 : Anti CD45 V500 / Anti CD3 Alexa488 + Anti human IgG4 Biotin + Excess VX15/2503 + SA-APC (Max Binding Control)

Percent Saturation = $\left| [(B-A) \div (C-A)] \times 100 \right|$

Where :

A = FL4 GMFI generated from assay panel Tube 9 which represents the background signal

B = FL4 GMFI generated from assay panel Tube 10 which represents the unknown sample

signal

C = FL4 GMFI generated from assay panel Tube 11 which represents the VX15/2503 mAb

saturated (maximum) signal

Note: Mastermixes for each tube/antibody combination are pre-made and brought up to 100 µL with FACS buffer

***G. Excess tumor tissue.***

When sufficient quantity permits, a section of fresh tumor samples will be split and snap frozen in liquid nitrogen either (1) alone for future genomic analysis or (2) in RNALater solution to facilitate future high throughput analysis of gene expression profile. Samples will be stored immediately in cryovials at -80°C for future analysis.

***H. Blood Specimen Processing.***

Blood samples will be collected prior to treatment at baseline (day 1), day 15, and the day of surgery (day 22 – 36), Three 10mL purple top tubes of blood containing EDTA as an anti-coagulant will be obtained for correlative studies at each of these time points. Blood samples will be processed within 4 hours of the blood draw using standard methods developed by the Chronic Pancreatitis, Diabetes, and Pancreatic Cancer (CPDPC) Consortium which is used as a standard operating procedure in the Lesinski Laboratory (see below). For the saturation assay on peripheral cells, whole blood will be collected in cytochex tubes and for PK, sSEMA4D, and ADA, serum will be collected in SST tubes. [39] Please refer to the Covance Lab Manual for the specifics of this blood collection / processing.

***H.1. Blood procurement for plasma and PBMCs.***

## 1. COLLECTION

1.1 Blood collection should be performed by a licensed phlebotomist, nurse, anesthesiologist, medical doctor, or a clinical research coordinator trained in phlebotomy.
1.2 Collection should be performed in an adequate setting, e.g. in the phlebotomy room, or on the ward. Blood collection in the operating theater should be avoided, if possible.

## 2. Processing

2.1 Appropriate precaution should be taken for all patient blood samples as the infectious status of specimens cannot always be determined.
2.2 All centrifuge spins should be performed using lids that prevent aerosol release from blood samples.

**Notes**

- This SOP applies to the collection of serum, plasma, buffy coat, and whole blood.
- This SOP does not cover blood withdrawal techniques/procedures (qualified personnel should follow standard withdrawal procedures).
- This SOP does not cover safety procedures for the collection and processing of these samples and personnel must follow institutional biosafety guidelines.
- This SOP does not cover informed consent procedures.
- Definition: g force (G) = relative centrifugal force (RCF)

### Plasma (EDTA tube)

1. Collect approximately 7mL blood in each of the two (2) EDTA blood collection tubes
2. Mix blood thoroughly after draw by inverting the tube 8 to 10 times.
3. Store vacutainer tubes upright at 4ºC (refrigerated or on wet ice) until centrifugation.
4. **Blood samples should be centrifuged, processed and in -80°C freezer within four hours of blood collection.**
5. Centrifuge at 1200g for 10 minutes at room temperature with the **BRAKE ON**. After centrifugation, the sample should separate into 2 layers: top layer is the plasma, and the bottom layer is the white (buffy coat) and red blood cells.
6. Carefully collect the plasma layer with an appropriate transfer pipette without disturbing the buffy coat from both EDTA tubes into a 15 mL conical tube.
7. Ensure sample homogeneity.
8. Aliquot using a pipet into 250 µL aliquots. After collecting (10) 250 µl aliquots divide the remainder of the samples into large volume, 1.8 mL aliquots (up to 6) and freeze. When requested, the 1.8 mL tubes should be completely thawed, vortexed and processed into 250 µL aliquots, labelled and logged into system.
9. Care should be taken to prevent disturbing the bottom layer which could result in contamination.

Notes

- Plasma should be free of hemolysis (reddish in color). If the sample is hemolyzed (reddish in color), aliquot the non-hemolyzed sample first, and then finish with the hemolyzed sample.

- Freeze plasma specimens below –80ºC no more than 4 hours after the blood draw. Plasma aliquots can be temporarily (< 4 hours) refrigerated or stored on wet ice (4ºC) until able to freeze at –80ºC. Samples should not be thawed prior to shipping. (Will be shipped on dry ice. Refer to Shipping and Receiving SOP.)

**Warning**: Excessive centrifuge speed (over 2000 g) may cause tube breakage and exposure to blood and possible injury. If needed, RCF for a centrifuge can be calculated. For an on-line calculator tool, please refer to:
http://www.changbioscience.com/cell/rcf.html or
http://insilico.ehu.es/mini_tools/rcf_rpm.php

## ***H.2. Buffy Coat***

1. Collect 7 mL of blood in each of the two (2) EDTA blood collection tubes.
2. Mix blood thoroughly after draw by inverting the tube 8 to 10 times.
3. Store vacutainer tubes horizontally at room temperature until centrifugation.
4. **Blood samples should be centrifuged, processed and in -80°C freezer within four hours of blood collection.**
    a. Centrifuge at 580g for 10 minutes with on the **BRAKE ON** at room temperature.
5. After centrifugation, the sample should separate into 2 layers: top layer is the plasma, and the bottom layer is the white and red blood cells.
    a. Using a transfer pipet, transfer the top plasma layer into a 50 mL conical tube and divide into (up to 10) 250 µL aliquots (label as plasma from buffy coat SOP).
    b. After collecting (10) 250 µl aliquots divide the remainder of the samples into large volume, 1.8 mL aliquots (up to 6) and freeze. When requested, the 1.8 mL tubes should be completely thawed, vortexed and processed into 250 µL aliquots, labelled and logged into system.
    c. Using a transfer pipet, transfer the entire bottom layer into a 50 mL conical tube with appropriate label.
6. Rinse the blood collection tube (EDTA) with (10-15 mL) of PBS using a pipet in order to collect all possible cells and transfer this PBS to the 50 mL tube containing the remaining blood.
    a. Fill the 50ml tube with PBS to a volume of 35 mL.
    b. Re-cap the tube, and mix well by inversion 3-4 times.
7. Underlay 13 mL of Ficoll in the 50 mL conical tube using a sterile 10 mL plastic pipette placed beneath the blood at the bottom of the tube, and releasing very slowly. A clear Ficoll layer should form at the bottom of the tube.
8. Carefully cap the tubes and centrifuge them for 30 minutes at 800g with the **BRAKE OFF** to prevent mixing of the layers.
9. Following the spin, the layers will be separated into 3 layers as follows: TOP – PBS, MIDDLE – buffy layer containing PBMCs, BOTTOM – Ficoll, RBC, Granulocytes. (See figure to the right) Using a sterile 10 mL plastic pipette, carefully collect the Buffy coat layer and put in a fresh 50 mL tube. It is OK to collect all of the top PBS layer to maximize cell yield. After the cells are collected, dilute the sample to a total of 45-50 mL by adding PBS and centrifuge the sample at 580g for 10 minutes with the **BRAKE ON**.

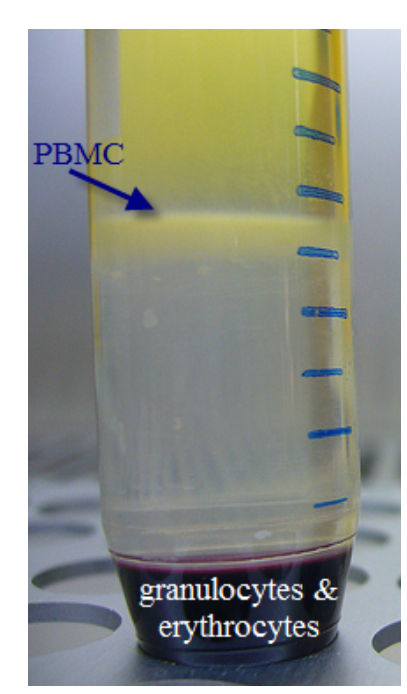


10. After the spin, gently pour off the supernatant taking care not to disturb the cell pellet. Re- suspend the pellet in 10 mL of AKC Lysis Buffer and incubate for 2 minutes. Fill the 50mL tube with PBS to a volume of 50mL and centrifuge the sample at 580g for 5 minutes with the **BRAKE ON.**

11. Aspirate the supernatant from the pellet. Resuspend the pellet in PBS. For a small pellet, use 5 mL PBS so you don't have to reconcentrate after counting. For large pellets, use up to 10 mL PBS for counting. Count and aliquot the re-suspended cells using a suitable method. This may include an automated coulter counter, or manually via trypan blue exclusion on a hemocytometer. An example of this method is presented as follows
    a. Take 10 µl of suspended pellet and mix with 90 µl of Trypan Blue (1:10) in a 0.5 mL microcentrifuge tube and count viable cells.
    b. Calculations: { [# of cells * $10^4$ * dilution factor (in this case 10)] / 4 }* volume of diluted PBMCs
    c. Add enough PBS to cell suspension above to bring volume up to 25 mL.
    d. Centrifuge the sample at 580 g for 5 minutes with the **BRAKE ON**.
    e. After centrifugation, discard PBS by gently pouring it off and re-suspend the pellet (this is your buffy coat containing PBMCs) with a suitable volume of Cell Freezing Media (Sigma) and transfer into a labeled cryovial. The volume is usually ~2 mL, however cells should ideally be in a concentration range of 3 x $10^6$ – 5 x $10^6$ cells per mL of cell freezing media. Multiple frozen aliquots of cells may be necessary.
    f. Place the tube into a pre-cooled (4°C) "Mr. Frosty" freezing container (Nalgene) and place in -80°C freezer.
    g. Remove Mr. Frosty from -80°C after 12-24hrs, place the cryovials in a cardboard freezer storage box, and then place in vapor phase of liquid

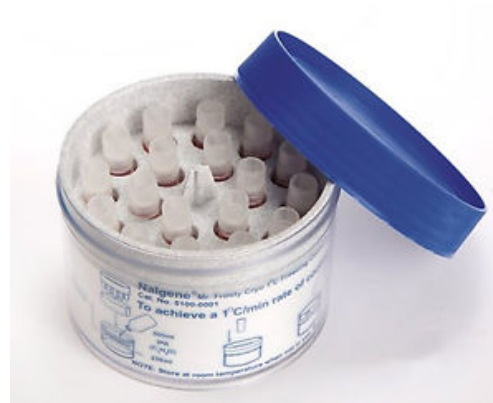

nitrogen freezer for long-term storage.

**Mr. Frosty Nalgene 5100-0001**

12. All samples (plasma, serum and buffy coat) should remain frozen until analysis.

***I. Cytokine or Soluble Factor Analyses:***

Cytokines in patient plasma will be analyzed using a high-throughput bioplex analysis as described by our group.[36] Our analysis will focus on a panel of cytokines and chemokines representing the following functional classes: 1) analytes shown by our group as expressed from pancreatic stellate cells; 2) cytokines/chemokines that are documented to promote MDSC or T regulatory cell expansion or migration; 3) cytokines involved in Th1/Th2/Th17 differentiation. All patient samples will be run in duplicate, and compared to pre-specified standard curves to obtain quantitative data. Duplicate samples displaying a CV>10% will be re-analyzed with additional replicates for greater accuracy. Given its relevance in both progression of PDAC and CRC, a separate aliquot of plasma will be utilized to assess expression of TGF-β via standard ELISA assay as this analyte is not compatible with the proposed bioplex analysis. Plasma samples from individual patient cohorts will be batched and analyzed simultaneously. Serum will also be collected for PK, sSEMA4D, and ADA that will be run at Vaccinex, Inc., using a standard protocol.

***J. Immune suppressor cell analyses:***

The expansion of immune suppressive cytokines and particular cell populations represent important barriers that likely limit the full potential of immune-based therapies or endogenous host responses to tumors. We hypothesize that this state of elevated immune suppressor cells in cancer patients can be manipulated via SEMA4D blockade to provide an advantage for the maximal therapeutic effect of immune checkpoint blockade. For these experiments, multiparameter flow cytometry will be utilized to phenotype immune effector cells to determine whether VX15/2503 containing regimens downregulate T regulatory cells (based on expression of CD4, CD25, CD127, CD49d and FoxP3), IL-10-producing B regulatory cells (based on expression of CD5, CD19, CD1d, CD24, CD25, CD27, CD38 and IL-10) and myeloid derived suppressor cells ($CD33^{+}HLADR^{-}CD11b^{+}$ plus $CD14^{+}$ or $CD15^{+}$) as these populations can neutralize host anti-tumor responses and could be modulated in response to VX15/2503. Namely, it is possible that VX15/2503 could directly interact with these cells, or the ability of this agent to limit the desmoplastic PDAC stroma could result in reduced expansion of these cell populations. Indeed, our group has previously data demonstrating that pancreatic stellate cells are a prominent source of IL-6 in the TME, and that its secretion from these cells can facilitate expansion of suppressive myeloid cell populations.[36] Briefly, these freshly procured cells will be aliquoted at $5 \times 10^{5} – 1 \times 10^{6}$ cells/flow tube in PBS containing 5% FBS (flow buffer). Cells will be incubated at 4°C with the following fluorochrome conjugated Ab for 45 minutes. Cells will then be washed twice with 2 mL flow buffer and pelleted via centrifugation at 805 *x g* for 10 minutes. Following decanting of flow buffer from the final wash, cells will be resuspended in 300 uL 1% formalin and stored in the dark at 4°C. Samples will be analyzed on a BD LSR II flow cytometer in the Emory University School of Medicine Flow Cytometry Core Facility. All data will be expressed as the percentage of total circulating peripheral blood cells with each respective phenotype. Appropriate fluorochrome labeled isotype control antibodies will be utilized for determination of background staining and for compensation. The phenotypes chosen represent the most well-accepted and published phenotypic definitions to date, and will be modified as concordance among the tumor immunology community becomes more apparent

***K. Analysis of T cell phenotype and function:***

We will analyze blood from the clinical trial by multi-parameter flow to identify systemic phenotypic shifts mediated by VX15/2503 in combination with immune checkpoint inhibition. This approach has been published by our group, and is continually refined with our collaborator, Dr. Ahmed to incorporate the most up-to-date markers of relevance to anti-PD-1 therapy.[36] Briefly, fresh peripheral blood samples will be obtained at baseline, 2 weeks into cycle 1 (day of second biopsy), and on the day of surgery. Using this method, we will characterize phenotypic shifts and function in T cell subsets. Data obtained in tumor biopsies including proliferative $CD8^{+}CD28^{+}Ki67^{+}$ T cell subsets will be compared to that measured in blood. For analysis of this subset in blood, flow cytometry will be used to assess cells with a more detailed phenotype ($CD8^{+}Ki67^{+}CD28^{+}Bcl2^{lo}CD38^{+}PD\text{-}1^{+}$) or ($CD8^{+}Ki67^{+}CD28^{+}Bcl2^{lo}CD38^{+}CTLA4^{+}$). In addition, we will study how VX15/2503 influences shifts in Th1/Th2/Th17 or Th subsets with phenotypic properties of naïve, central and effector memory cells. These data will be complemented by *ex vivo* analysis of function (intracellular cytokine staining, $Ki67^{+}$ for proliferation) or functional markers (CD107a) in aliquots of freshly isolated T cells stimulated with PMA/Ionomycin or CD3/CD28-beads as per standard protocol. For CD107a staining, the BD FastImmuneTM CD107a assay will be utilized per manufacturer's instructions. For intracellular cytokine stains, following re-stimulation of PBMCs, cells will be incubated with Golgi-stop (BD Biosciences) for 4 hours and different immune cell populations will be assessed for intracellular IFN-□□or TNF-α (BD Biosciences) production. Specific antibodies include CD3-AlexaFluor 488, CD56-AlexaFluor 657, CD11c-APC, CD14-AlexaFluor 488, CD66b-AlexaFluor 647, IFN-γ-PE or TNF-α-PE (BD Biosciences). Appropriate isotype control antibodies for each fluorochrome will be used as negative

controls. All samples will be run on a BD LSR II flow cytometer, and analyzed with FlowJo (Tree Star, Inc.). Finally, VX15 saturation on T cells will be completed as described above.

## 15 REFERENCES

---


[1] Siegel R, Miller K, et al. Cancer Statistics, 2017. *Cancer: A Cancer Journal for Clinicians*. 2016;N/A.
[2] Hodi FS, O'Day SJ, McDermott DF, et at. Improved Survival with Ipilimumab in Patients with Metastatic Melanoma. *NEJM*. 2010;363:711-723.
[3] Ribas A, Puzanov I, Dummer R, et al. Pembrolizumab versus investigator-choice chemotherapy for ipilimumab-refractory melanoma (KEYNOTE-002): a randomized, controlled, phase 2 trial. *Lancet Oncol*. 2015;16:908-918.
[4] Schadendorf D, Hodi FS, Robert C, et al. Pooled analysis of long-term survival data from Phase II and III trials of ipilimumab in unresectable or metastatic melanoma. J Clin Oncol. 2015;33:1889-1894.
[5] Robert C, Schachter J, Long G, et al. Pemborlizumab versus ipilimumab in advanced melanoma. *NEJM*. 2015;372:2521-2532.
[6] Wolchok JD, CHiarion-Sileni V, Gonzalez R, et al. Overall Survival with Combined Nivolumab and Ipilimumab in Advanced Melanoma. *NEJM*. 2017;377:1345-1356.
[7] Eggermont AM, Chiarion-Sileni V, Grob JJ, et al. Adjuvant ipilimumab versus placebo after complete resection of high-risk stage III melanoma (EROTC 18071): a randomized double-blind, phade 3 trial. *Lancet Oncol*. 2015;16:522-530.
[8] Weber J, Mandala M, Del Vecchio HJ, et al. Adjuvant nivolumab versus ipilimumab in resected stage III or IV melanoma. *NEJM*. 2017;377:1824-35.
[9]Wu M, Li J, Gao Q, et al. The role of Sema4/CD100 as a therapeutic target for tumor microenvironments and for autoimmune, neuroimmune and bone disease. *Expert Opin Ther Targets* 2017;20(7):885-901.
[10] Zhu L, Bergmeier W, Wu J, et al. Regulated surface expression and shedding support a dual role for semaphoring 4D in platelet response to vascular injury. *Proc Natl Acad Sci* 2007;104:1621-6.
[11] Conrotto P, Valdembri D, Corso S, et al. Sema4D induces angiogenesis through Met recruitment by Plexin B1. *Blood* 2005;105: 4321-4329.
[12] Tamagnone L, Artigiani S, Chen H, et al. Plexins are a large family of receptors for transmembrane, secreted, and GPI-anchored semaphorins in vertebrates. *Cell* 1999;99: 71-80.
[13] Takamatsu H, Takegahara N, Nakagawa Y, et al. Semaphorins guide the entry of dendritic cells into the lymphatics by activating myosin II. Nat. *Immunol* 2010; 11: 594-600.
[14] Conrotto P, Corso S, Gamberini S, et al. Interplay between scatter factor receptors and B plexins controls invasive growth. *Oncogene* 2004;23: 5131-5137.
[15] Giordano S, Corso S, Conrotto P, et al. The semaphorin 4D receptor controls invasive growth by coupling with Met. *Nat. Cell Biol*. 2002;4: 720-724.
[16] Valente G, Nicotra G, Arrondini M, et al. Co-expression of plexin-B1 and Met in human breast and ovary tumours enhances the risk of progression. *Cell Oncol*. 2009;31: 423-436.
[17] Worzfeld T, Swiercz JM, Looso M, et al. ErbB-2 signals through Plexin-B1 to promote breast cancer metastasis. *J Clin. Invest* 2012;122: 1296-1305.
[18] Witherden DA, Watanabe M, Garijo O, et al. The CD100 Receptor Interacts with Its Plexin B2 Ligand to Regulate Epidermal gammadelta T Cell Function. *Immunity* 2012; 37: 314-325.
[19] Kumanogoh A, Watanabe C, Lee I, et al. Identification of CD72 as a lymphocyte receptor for the class IV semaphorin CD100: a novel mechanism for regulating B cell signaling. *Immunity* 2000; 13: 621-631.

[20] Evans EE, Jonason, Jr. AA, Bussler H, et al. Antibody blockade of semaphorin 4D promotes immune infiltration into tumor and enhances response to other immunomodulatory therapies. *Cancer Immunol. Res*. 2015;3: 689-701.
[21] Evans EE, Paris M, Smith ES, and M. Zauderer. Immunomodulation of the tumor microenvironment by neutralization of Semaphorin 4D. *Oncoimmunology* 2015; 4: e1054599.
[22] Fisher TL, Reilly CA, Winter LA, et al. Generation and preclinical characterization of an antibody specific for SEMA4D. *MAbs*.2015; 1-13.
[23] Wannemacher KM, Wang L, Zhu L, Brass LF.The role of semaphorins and their receptors in platelets: Lessons learned from neuronal and immune synapses. *Platelets*. 2011;22: 461-465.
[24] Leonard JE, Fisher TE, Winter L, et al. Nonclinical Safety Evaluation of VX15/2503: a Humanized IgG4 Anti-SEMA4D Antibody. *Molec Can Therap* 2015; doi: 10.1158/1535-7163.MCT-14-0924
[25] Romer PS, Berr S, Avota E, et al. Preculture of PBMC at high cell density increases sensitivity of T-cell responses, revealing cytokine release by CD28 superagonist TGN1412. *Blood* 2011;118: 6772-6782.
[26] Patnaik, A, et al., Phase 1 Study of VX15/2503, a humanized IgG4 anti-SEMA4D antibody, in advanced cancer patients, *Clin Can Research* 2015, DOI: 10.1158/1078-0432.CCR-15-0431
[27] Chambers CA, Kuhns MS, Egen JG, et al. CTLA-4-mediated inhibition in regulation of T cell responses: Mechanisms and manipulation in tumor immunotherapy. *Annu Rev Immuno* 2001;19:565-594.
[28] Zou W, Wolchok JDD, and Chen L. PD-L1 (B7-H1) and PD-1 Pathway Blockade for Cancer Therapy: Mechanisms, Response Biomarkers and Combinations *Sci Transl Med* 2016;8:328rv4
[29] Lark J, Chiaron-Sileni V, Gonzalez R, et al. Combined Nivolumab and Ipilimumab or Monotherapy in Untreated Melanoma. *N Engl J Med* 2015;373:23-34.
[30] Motzer RJ, Escudier B, McDermott DF, et al. Nivolumab versus Everolimus in Advanced Renal Cell Carcinoma. *N Engl J Med* 2015;373:1803-1813.
[31] Ferrs RL, Blumenschein G, Fayette J, et al. Nivolumab for Recurrent Squamous-Cell Carcinoma of the Head and Neck. *N Engl J Med* 2016;375:1856-1867.
[32] Brahmer J, Reckamp KL, Baas P, et al. Nivolumab versus Docetaxel in Advanced Squamous-Cell Non-Small Cell Lung Cancer. *N Engl J Med* 2015;373-123-135.
[33] Sharma P, Retz M, Siefker-Radtke A, et al. Nivolumab in metastatic urothelial carcinoma, after platinum therapy (CheckMate 275): a multicenter, single-arm, phase 2 trial. *Lancet Oncol* 2017;18:312-322.
[34] Markowitz J, Brooks TR, Duggan MC, et al. Patients with pancreatic adenocarcinoma exhibit elevated levels of myeloid-derived suppressor cells upon progression of disease. *Cancer Immunol Immunother*. 2015;64:149-59.
[35] Farren M, Mace TA, Geyer S, et al. Systemic Immune Activity Predicts Overall Survival in Treatment-Naïve Patients with Metastatic Pancreatic Cancer. *Clin Cancer Res*, 2016;22(10):2565-74.
[36] Mace TA, Ameen Z, Collins A et al. Pancreatic cancer-associated stellate cells promote differentiation of myeloid –derived suppressor cells in a STAT3-dependent manner. *Cancer Res,* 2013;73:3007-18.
[37] Noonan AM, Farren MR, Geyer, SM, et al. Randomized Phase II Trial of the Oncolytic Virus Pelareorep (Reolysin) in Upfront Treatment of Metastatic Pancreatic Adenocarcinoma. *Molecular Therapy* 2016, Jun;24(6):1150-1158.

[38] Sckisel GD, Mirsolian A, Minnar CM et al. Differential phenotypes of memory CD4 and CD8 T cells in the spleen and peripheral tissues following immunostimulatory therapy. J Immunother of Cancer, 2017;5:33.
[39] Fisher T et al. Saturation monitoring of VX15/2503, a novel semaphorin 4D-specific antibody, in clinical trials. Cytometry B-Clinical Cytometry. 90(2): 199-208.
[41] Evans EE, Bussler H, Torno S et al. Antibody blockade of semaphoring 4D breaks down stromal barriers to enhance tumorcidal immune infiltration, supporting rational immunotherapy combinations. AACR 107th Annual Meeting 2016, New Orleans, LA: abstract 4888.
