# Supplementary material for: Neoadjuvant pepinemab in combination with nivolumab and/or ipilimumab in resectable metastatic melanoma: a pilot biomarker-driven trial
Source: Nat Commun. 2026 Oct 6;17:11377. doi: 10.1038/s41467-026-78231-3 (PMC13736988; doi:10.1038/s41467-026-78231-3)
Supplement: Supplementary file 2 [file 41467_2026_78231_MOESM2_ESM.pdf]

nature portfolio

Corresponding author(s): Chrystal M Paulos, Micheal C. Lowe, Gregory B Lesinski

Last updated by author(s): 09/15/2026

Reporting Summary

Nature Portfolio wishes to improve the reproducibility of the work that we publish. This form provides structure for consistency and transparency in reporting. For further information on Nature Portfolio policies, see our Editorial Policies and the Editorial Policy Checklist.

Statistics

For all statistical analyses, confirm that the following items are present in the figure legend, table legend, main text, or Methods section.

n/a	Confirmed	
☐	☒	The exact sample size (*n*) for each experimental group/condition, given as a discrete number and unit of measurement
☒	☐	A statement on whether measurements were taken from distinct samples or whether the same sample was measured repeatedly
☐	☒	The statistical test(s) used AND whether they are one- or two-sided<br>*Only common tests should be described solely by name; describe more complex techniques in the Methods section.*
☒	☐	A description of all covariates tested
☐	☒	A description of any assumptions or corrections, such as tests of normality and adjustment for multiple comparisons
☐	☒	A full description of the statistical parameters including central tendency (e.g. means) or other basic estimates (e.g. regression coefficient) AND variation (e.g. standard deviation) or associated estimates of uncertainty (e.g. confidence intervals)
☐	☒	For null hypothesis testing, the test statistic (e.g. *F*, *t*, *r*) with confidence intervals, effect sizes, degrees of freedom and *P* value noted<br>*Give P values as exact values whenever suitable.*
☒	☐	For Bayesian analysis, information on the choice of priors and Markov chain Monte Carlo settings
☒	☐	For hierarchical and complex designs, identification of the appropriate level for tests and full reporting of outcomes
☒	☐	Estimates of effect sizes (e.g. Cohen's *d*, Pearson's *r*), indicating how they were calculated

Our web collection on statistics for biologists contains articles on many of the points above.

Software and code

Policy information about availability of computer code

Data collection	Graph Pad Prism v11, GeoMx_NGS_Pipeline_3.1.1.6 software
Data analysis	GeoMxWorkflows_1.12.0<br>GeomxTools_3.10.0<br>umap_0.2.10.0<br>fgsea_1.32.2<br>org.Hs.eg.db_3.19.1<br>ggplot2_3.5.1<br>MCPcounter_1.2.0<br>ComplexHeatmap_2.22.0<br>Visiopharm Oncotopix®

For manuscripts utilizing custom algorithms or software that are central to the research but not yet described in published literature, software must be made available to editors and reviewers. We strongly encourage code deposition in a community repository (e.g. GitHub). See the Nature Portfolio guidelines for submitting code & software for further information.

Data

Policy information about availability of data

All manuscripts must include a data availability statement. This statement should provide the following information, where applicable:

- Accession codes, unique identifiers, or web links for publicly available datasets
- A description of any restrictions on data availability
- For clinical datasets or third party data, please ensure that the statement adheres to our policy

The datasets generated during and/or analyzed during the current study for clinical trial NCT03769155 are available on NCBI Gene Omnibus, Emory cohort: GSE332987 PRJNA1469599, MDACC cohort: GSE331322, PRJNA1467675. The Adaptive BCR and TCR sequencing data have been deposited in the ImmuneACCESS database (Adaptive Biotechnologies) under DOI:10.21417/ATR2026S and URL https://clients.adaptivebiotech.com/pub/ruffin-2026-s Nanostring n counter raw and normalized counts are available within the source data file. Source data are provided with this paper and are available as a Source Data file. Given the small cohort size individual de-identified participant data will not be published; however, clinical data will be available from the corresponding author upon request. The study protocol is available in the Supplementary Information. The remaining data are available within the Article, Supplementary Information or Source Data file or available from the authors upon request.

Research involving human participants, their data, or biological material

Policy information about studies with human participants or human data. See also policy information about sex, gender (identity/presentation), and sexual orientation and race, ethnicity and racism.

Reporting on sex and gender

Sex was self reported but not relevant to study comparisons and data analysis

Reporting on race, ethnicity, or other socially relevant groupings

Race was self reported but not used in data analysis and comparisons

Population characteristics

41 patients enrolled
Inclusion Criteria:
Stage IIIB, IIIC, IIID histologically-proven melanoma.

Cancer confirmed to be surgically resectable, with surgery evaluation with planned prior to resection.
No prior immunotherapy with cytotoxic T-lymphocyte associated protein-4 (CTLA-4), anti programmed cell death-1 (PD-1) or VX15/2503. Prior interferon (at least 1 year prior to consent) will be allowed.
Average Age 60

Recruitment

patients were recruited that matched the following criteria: Patient-specific criteria
Informed consent
Age > 18 years
ECOG performance status of 0 or 1
Adequate bone marrow function (leukocyte count >2,000/mm3; platelet count >100,000/mm3; and hemoglobin count >9.0gm/dL)
Adequate renal function (creatinine clearance > 50 mL/min; or serum creatinine level <1.5 mg/dL)
Adequate hepatic function (bilirubin < 1.5 x upper normal limit; AST, ALT, and alkaline phosphatase <2.5 x upper normal limit)
INR ≤1.5. (Anticoagulation is allowed if target INR ≤ 1.5 on a stable dose of warfarin).
Patient receiving LMW heparin on stable therapeutic dose for more than 2 weeks or with factor Xa level <1.1U/mL are allowed on the trial.
Disease-specific criteria:
Biopsy-accessible surgically resectable stage IIIB/IIIC/IIID melanoma (AJCC 8th edition)
No prior immunotherapy for melanoma. Prior interferon will be allowed as long as it was greater than 1 year prior to consent.

Ethics oversight

Emory University Institutional Review Board (IRB00104273)

Note that full information on the approval of the study protocol must also be provided in the manuscript.

Field-specific reporting

Please select the one below that is the best fit for your research. If you are not sure, read the appropriate sections before making your selection.

[x] Life sciences [ ] Behavioural & social sciences [ ] Ecological, evolutionary & environmental sciences

For a reference copy of the document with all sections, see nature.com/documents/nr-reporting-summary-flat.pdf

Life sciences study design

All studies must disclose on these points even when the disclosure is negative.

Sample size	Sample size was determined using a power analysis. Assuming a CV of 50%, n=8 patients the in treatment groups and 6 patients in the control group can achieve at least 80% power to detect a 3-fold difference in change of expression by treatment between the pairwise groups (α=0.0125, 2-sided).
Data exclusions	No data was excluded
Replication	As this was a pilot biomarker trial, no replication was conducted
Randomization	Randomization was not applicable to this study.
Blinding	Blinding was not relevant to this study as investigators needed to know treatment groups to do comparisons

Reporting for specific materials, systems and methods

We require information from authors about some types of materials, experimental systems and methods used in many studies. Here, indicate whether each material, system or method listed is relevant to your study. If you are not sure if a list item applies to your research, read the appropriate section before selecting a response.

Materials & experimental systems

n/a	Involved in the study	
☐	☒	Antibodies
☒	☐	Eukaryotic cell lines
☒	☐	Palaeontology and archaeology
☒	☐	Animals and other organisms
☐	☒	Clinical data
☒	☐	Dual use research of concern
☒	☐	Plants

Methods

n/a	Involved in the study	
☒	☐	ChIP-seq
☐	☒	Flow cytometry
☒	☐	MRI-based neuroimaging

Antibodies

Antibodies used

CD20 L26 Thermo MA513141 1:100
CD4 EPR6855 Abcam ab133616 1:100
FoxP3 236A/E7 Thermo 14-4777-82 1:40
CD8 C8/144B Thermo MA513473 1:50
CD21 2G9 Cell Signaling 76069S 1:100
CD23 E1R4G Cell Signaling 87960 1:800
CXCR5 D6L3C Cell Signaling 72172S 1:100
Sema4D EP3569 Abcam ab134128 6ug/ml
CD11c EP1347Y Abcam ab52632 1:500
CD141 141C01 Thermo MA5-11454 1:50
CD68 D4B9C Cell Signaling 76437S 1:400
HLA-DR TAL 1B5 Abcam ab20181 1ug/ml
PD-L1 E1L3N Cell Signaling 13684S 1:200
Sox-10 EP268 Cell Marque 383R-18 1:100
VX15-2503 unconjugated 20µg/mL
anti-chicken IgY Biotin 10µg/mL Southern Biotech 8320-08
anti-human IgG4 Biotin 10µg/mL Southern Biotech 9200-08
PlexinB1 unconjugated 1µg/mL R&D Systems MAB37491
Live/Dead Ghost Dye 780 1:1000 Tonbo Biosciences 13-0865-T100
CD45 Pe-Cy7 1:100 Biolegend 368532
anti-mouse CD8 FITC 1:100 Biolegend 1007806

streptavidin-APC 1:5 BD Biosciences 349024
CD3 BV711 1:100 Biolegend 344838
CD4 AF700 1:100 Biolegend 344622
CD8 BV650 1:100 Biolegend 301041
CD19 PerCP-Cy5.5 1:100 Biolegend 302320
CD56 BUV395 1:100 BD Biosciences 563554
CD38 BV605 1:100 Biolegend 303532
HLA-DR BV785 1:100 Biolegend 307642

	CD26 PECy5 1:100 Biolegend 302708<br>PD1 BV421 1:100 Biolegend 367421<br>Tim3 BV510 1:100 Biolegend 345030<br>IgG PE 1:100 Biolegend 400114<br>LAG3 PE 1:100 Biolegend 369306<br>Ki67 PE-Dazzle594 1:100 Biolegend 350534<br>CD11b PeCy5 1:100 Biolegend 101210<br>CD11c BV421 1:100 Biolegend 337226<br>CD14 PerCP-Cy5.5 1:100 Biolegend 325622<br>CD15 Pe-Dazzle594 1:100 Biolegend 323038<br>CD16 AF700 1:100 Biolegend 302026<br>CD33 BV785 1:100 Biolegend 303428<br>CD40 BV650 1:100 Biolegend 334338<br>HLA-DR BV510 1:100 Biolegend 307646<br>CD103 BV605 1:100 Biolegend 350218<br>anti-mouse F(ab) APC 1:100 R&D Systems F0101B<br>IgG PE 1:100<br>IgG BV711 1:100 Biolegend 400354<br>PlexinB2 PE 1:100 R&D Systems FAB53291P<br>PDL1 BV711 1:100 Biolegend 329722
Validation	Commercial antibody validation was performed by manufacturer. VX15-2503 is Pepinemab and was validated by Vaccinex

Clinical data

Policy information about clinical studies

All manuscripts should comply with the ICMJE guidelines for publication of clinical research and a completed CONSORT checklist must be included with all submissions.

Clinical trial registration	NCT03769155
Study protocol	Clinical Trials.gov
Data collection	Data was collected at Emory University. All data was collected by December 31st 2023.
Outcomes	Primary objectives<br>Evaluate the effect of Pepinemab in combination with immune checkpoint inhibitors on T cell infiltrate into the tumor microenvironment in involved and uninvolved lymph nodes and peripheral blood.<br>Secondary Objectives<br>Evaluate the effect of Pepinemab in combination with immune checkpoint inhibitors on the immune profile of involved and uninvolved lymph nodes and peripheral blood.<br>Assess safety and tolerability of profile and tolerability of single agent Pepinemab to the combination of Pepinemab and immune checkpoint inhibitors in patients with resectable metastatic melanoma.<br>Document pathologic response rates of Pepinemab and immune checkpoint inhibitors in patients with resectable melanoma.<br>Compare pathologic response to radiographic response using RECIST criteria in patients receiving single agent Pepinemab and combination Pepinemab and immune checkpoint inhibitors in patients with resectable melanoma

Plants

Seed stocks	*Report on the source of all seed stocks or other plant material used. If applicable, state the seed stock centre and catalogue number. If plant specimens were collected from the field, describe the collection location, date and sampling procedures.*
Novel plant genotypes	*Describe the methods by which all novel plant genotypes were produced. This includes those generated by transgenic approaches, gene editing, chemical/radiation-based mutagenesis and hybridization. For transgenic lines, describe the transformation method, the number of independent lines analyzed and the generation upon which experiments were performed. For gene-edited lines, describe the editor used, the endogenous sequence targeted for editing, the targeting guide RNA sequence (if applicable) and how the editor was applied.*
Authentication	*Describe any authentication procedures for each seed stock used or novel genotype generated. Describe any experiments used to assess the effect of a mutation and, where applicable, how potential secondary effects (e.g. second site T-DNA insertions, mosiacism, off-target gene editing) were examined.*

Flow Cytometry

Plots

Confirm that:

- [x] The axis labels state the marker and fluorochrome used (e.g. CD4-FITC).
- [x] The axis scales are clearly visible. Include numbers along axes only for bottom left plot of group (a 'group' is an analysis of identical markers).
- [x] All plots are contour plots with outliers or pseudocolor plots.
- [x] A numerical value for number of cells or percentage (with statistics) is provided.

Methodology

Sample preparation	Patient tissue resections were stored in hypothermosol for 24 hours at 4C prior to manual dissociation with a razor, followed by digestion with the GentleMACS Tumor Dissociation Kit (Miltenyi Biotec cat no. 130-095-929). The resulting cell suspension was stained using antibodies listed in (supplemental table 1). Surface stains were incubated at 4C for thirty minutes in the dark. Cells were then fixed using Cytofix and SEMA4D saturation samples were stored at 4C overnight. Samples were analyzed on a FACSymphony flow cytometer the following day. Where appropriate, samples were then briefly washed with PermWash before freezing in 90%FBS/10% DMSO (freezing media) until batch staining for intracellular markers. Batch staining was performed on frozen TILs and patient PBMCs. PBMCs were thawed in a 37C water bath, centrifuged to remove freezing media, then plated to be surface stained. TILs were thawed in PermWash in a 37C incubator, then centrifuged to remove freezing media before plating with surface stained PBMCs. All samples were then stained for intracellular markers in PermWash at 4C in the dark for 1 hour. TILs and PBMCs were run on a FACSymphony flow cytometer and cell populations were identified and analyzed using FlowJo (BD Biosciences).
Instrument	FACSymphony
Software	Flowjo v10
Cell population abundance	Cell sorting was not performed in this study
Gating strategy	Lymphocytes were gated using SCA and FSC. Single cells were then gated using FSC-H and FSC-A. Live cells were gated using SCA and Live dead viability dye. We gated CD45+ cells then CD3+ T cells. CD19 cell were gated from the CD3 - fraction

- [x] Tick this box to confirm that a figure exemplifying the gating strategy is provided in the Supplementary Information.
