# Supplementary material for: Neoadjuvant pepinemab in combination with nivolumab and/or ipilimumab in resectable metastatic melanoma: a pilot biomarker-driven trial
Source: Nat Commun. 2026 Oct 6;17:11377. doi: 10.1038/s41467-026-78231-3 (PMC13736988; doi:10.1038/s41467-026-78231-3)
Supplement: Supplementary file 3 [file 41467_2026_78231_MOESM3_ESM.pdf]

nature portfolio

Peer Review File

# Neoadjuvant pepinemab in combination with nivolumab and/or ipilimumab in resectable metastatic melanoma: a pilot biomarker-driven trial

Corresponding Author: Dr Gregory B. Lesinski

**This file contains all reviewer reports in order by version, followed by all author rebuttals in order by version.**

Version 0:

Reviewer comments:

Reviewer #1

(Remarks to the Author)
This is a pilot study that evaluated pepinemab, a Semaphorin 4D (SEMA4D)–blocking antibody, in combination with nivolumab and/or ipilimumab in a neoadjuvant trial for patients with resectable melanoma stage IIIB/C/D melanoma. Patients received two neoadjuvant doses followed by surgery on day 42 and one year of adjuvant nivolumab. Patients were sequentially enrolled into one of four treatment cohorts (n=8 each): pepinemab/nivolumab, pepinemab/ipilimumab, pepinemab/nivolumab/ipilimumab, or nivolumab alone as well one arm of surgery alone (n = 6). pCR/MPR rates were 25%/37.5%, 12.5%/12.5%, 62.5%/75%, 25%/37.5% with pep/nivo, pep/ipi, pep/nivo/ipi and nivo alone respectively. Mechanistically, biomarker analysis of patient tumors reported significant immunomodulatory changes with pepinemab in combination with ICI. Overall, the study results support the safety, feasibility, and immune modulatory activity of pepinemab when administered in melanoma in the neoadjuvant setting together with ICI.

The study is interesting as a safety, feasibility and immune monitoring study but is limited by the too ambitious design of 5 study arms with small sample size in each, asking too many questions and making conclusive results hard to attain. For example, what is the recommended next step in testing pepinemab in the neoadjuvant setting of melanoma? The pCR/MPR rates were identical between the pepinemab/nivolumab arm and nivolumab alone arm. Does that mean that pepinemab has no added value to PD1 blockade? What is the value of the surgery alone arm as part of this study. Therefore, it may have been more interesting to evaluate 2 or 3 study arms allowing more definitive analyses supporting more conclusive recommendations and future directions.

The study also lacked a pepinemab monotherapy arm which would have been ideal to better understand the added impact of pepinemab to ICI. Alternatively, would have been useful to initiate systemic therapy with one dose of pepinemab while initiating ICI a week or 2 later while obtaining biopsies at baseline, then right before ICI addition and then while on-treatment with the combination.

The lack of randomization is also a limitation in order to minimize confounding factors that may impact the clinical as well as the mechanistic analyses that can be attributed to sequential enrollment spanning over a number of years.

The authors note that no patients experienced toxicity that required delay or discontinuation of therapy or planned surgery. What were the range and median duration of time from study registration to surgery that was planned at day 42?

Mechanistically, the authors note that pepinemab plus ICI induced pathologic responses associated with immune remodeling, including increased T and B cell infiltration and enrichment of type 1 conventional dendritic cells. These changes promoted formation of mature lymphoid aggregates resembling tertiary lymphoid structures that harbored clonally expanded T cell receptor repertoires, consistent with localized cytotoxic activity. Aren't such changes expected in pathologic responses following anti-PD1 as monotherapy or anti-PD1 + anti-CTLA4? It is not clear how the study proves the added impact of pepinemab in mediating these immunomodulatory changes within the TME as compared to PD1 blockade alone or in combination with CTLA4 blockade.

The authors note that "Melanoma clinical stage was 15% stage IIIB, 46% IIIC and 38% IIID". This adds up to 99%.

Similarly, the authors note that “46% of patients had de novo presentation and 51% had prior surgical management of their primary disease”. These add up to 97%. What about the other 3%.

Toxicity is only presented briefly. A table that summarizes toxicities by grade should be included.

The authors report the study as a randomized phase II trial, but in the study protocol it is labelled as a pilot study.

Reviewer #2

(Remarks to the Author)
In their manuscript 'Neoadjuvant Pepinemab Enhances Immune Checkpoint Blockade in Patients with Metastatic Melanoma' the authors describe a small phase 1b trial testing a SEMA4D inhibitor in combination with neoadjuvant nivolumab or nivolumab plus ipilimumab in macroscopic stage III melanoma and present here in addition the translational research work.

While being an interesting concept the authors ignore completely the GCP of reporting on small cohorts and over-interpret their data completely. The whole manuscript must be adjusted in toning done the wording in regards of claiming efficacy and enhancement.

Major:
1) Introduction claims that many patients who initially benefit still progress. This is not true in the neoadjuvant setting
2) Key resistance mediators described in the introduction have not been shown in the neoadjuvant setting - thus you need to adjust the wording
3) With 62.5% grade 3/4 tax in the triplet arm and 37.5% in the doublet arm, which is twice the grade 3/4 too as compared to trAE grade 3/4 in NADINA (30%) and SWOG1801 (15%). So how come you to the conclusion that this is well tolerated?
4) You cannot conclude that the response was highest in the pepinemab containing arms, as the trial is clearly underpowered (as you state yourself) for comparisons
5) The arms have not been stratified for stage which makes any comparative clinical response conclusions in these very imbalanced cohorts absolutely unacceptable. At best case you can word this as giving some indication for a potential efficacy and really need to mention this in the results section and discuss in the discussion.
6) the baseline characteristics of the ipi/nivo cohort are missing
7) why have you not also analysed the nivo cohort from MDA which would have included at least one treatment scheme in both cohorts
8) all TR analyses must compare nivolumab versus nivo+pep and ipilimumab+nivolumab vs ipi+nivo+pep and only in case of statistical significance you can speak of increase/superiore, etc. E.g. the conclusions in Fig 2b are completely overstated with the ipi+nivo cohort missing. All observations here could be also due to the addition of ipi.
9) Figure 2d is illustrative. If you draw any conclusion you need to quantify for all patients
10) Figure 3b only triplet versus nivo is significant. How can you conclude that the addition of pep does the effect?
11) adjust all TR analyses and figure descriptions according to the suggestions above

Minor:
1) Change the title stating that it is tested in the neoadjuvant setting, remove the enhance and use a more neutral wording
2) In the introduction the current efficacy data on neoadjuvant pembrolizumab (SWOG1801), ipilimumab + nivolumab (NADINA) and nivo/rela are missing
3) please describe in the introduction more clearly for a non expert where SEMA4D in expressed and functions as ligand for its receptors expressed on which immune cells at which stage.

Reviewer #3

(Remarks to the Author)
The manuscript reports results from a pilot integrated biomarker study in which 38 patients with resectable metastatic melanoma were assigned to five treatment arms. The study addresses an important translational question in neoadjuvant melanoma and presents biologically rich, multi platform correlative data with potential impact. However, the current manuscript overinterprets certain causal and comparative findings given the small, non randomized cohorts; the absence of a contemporaneous nivolumab+ipilimumab control arm; the reliance on an external “synthetic control”; and the exploratory nature of many correlative analyses. The manuscript would benefit substantially from a more disciplined framing as a biomarker focused, hypothesis generating trial, accompanied by clearer reporting of patient flow and analysis denominators. More specifically,
1. The protocol and statistical plan describe this as a pilot integrated biomarker study with hypothesis generating objectives, yet portions of the manuscript imply that the trial supports comparative clinical efficacy conclusions. The manuscript should consistently differentiate biomarker endpoints from exploratory clinical outcomes and avoid language suggesting definitive comparative benefit.
2. The manuscript acknowledges that randomization across all five arms was not feasible. Expanded discussion is needed on the resulting risks of temporal drift, confounding, and arm imbalance, particularly in the context of cross arm comparisons.
3. The absence of a contemporaneous nivolumab+ipilimumab control arm is a major design limitation. Use of the external MD Anderson “synthetic control” should be characterized strictly as contextual or supportive rather than comparative

evidence. Additional detail is needed on how batch or cohort effects were handled.
4. The manuscript notes that 38 patients were enrolled and 32 treated, but it remains difficult to track how many patients contributed to each correlative analysis, each survival analysis, and each comparison set. A CONSORT style flow diagram and a table summarizing assay specific denominators by arm would improve transparency.
5. Overstatements in the discussion should be tempered. Phrases implying that pepinemab "improves" clinical benefit or that the data demonstrate distinct contributions beyond dual ICI should be revised to more cautious language such as "is associated with," "supports the hypothesis," or "warrants evaluation in a larger trial."
6. For figure panels relying on ordinary ANOVA or Fisher's exact tests in very small cohorts, effect sizes and confidence intervals would be more informative than isolated P values.

Minor revisions:
• For all main and extended figures, include the exact n per arm and per assay in each legend.
• Follow up time for recurrence analyses should be reported explicitly (median follow up, range, and censoring pattern).
• Page 7: "based on" appears twice in the sentence beginning "A more comprehensive multiplexed IHC analysis…".
• Add confidence intervals to Figure 1b.
• Include the surgery only arm in Figure 1d.
• The number of samples described in the legend does not match the number of plotted data points in Figure 2b. Missing data in the "pepi + ipi" group may explain fewer observations, but the appearance of more plotted lines than specified (e.g., nine in "nivo" when n=8; seven in "pepi + nivo" when n=5) requires clarification.
• Clarify why the "nivo" group is omitted from Figure 5.
• Extended Data Figure 5a may contain a typo in the y axis label.
• Extended Table 1 is missing a table title.

Reviewer #4

(Remarks to the Author)
I co-reviewed this manuscript with one of the reviewers who provided the listed reports. This is part of the Nature Communications initiative to facilitate training in peer review and to provide appropriate recognition for Early Career Researchers who co-review manuscripts.

Version 1:

Reviewer comments:

Reviewer #1

(Remarks to the Author)
My original comments have been addressed. No additional concerns.

Reviewer #2

(Remarks to the Author)
The reviewers addressed all my concerns except for one:

The 30% and 15% grade 3/4 AEs are the reported systemic treatment related adverse events. The additional 17% you are referring to are the surgery related AEs. Considering the fact that you are reporting only the systemic therapy related AEs the statement must be that the triplet in your work doubles/quadruples the systemic therapy grade 3/4 toxicity as compared to ipi/nivo and pembro neoadj respectively.

This needs to be discussed because such a considerable more toxic therapy should be therefore considered only for patients with highly unfavroabel markers unlikely to respond to the NADINA and SWOG schemes.

Reviewer #3

(Remarks to the Author)
Most of our prior comments have been fully addressed. However, we noted several inconsistencies between the authors' notes in the response file and the revised manuscript/supplemental files:

1.We could not identify a table labeled as Extended Data Table 1. In addition, the clinical stage names are inconsistent between the paragraph and the table (IIIB–IIID versus IIIA–IIIC). The percentages reported for the clinical stages in the paragraph also do not fully align with the values shown in the table, for example, 15.4% versus 15.5% and 46.2% versus

46%. The total percentage reported below also appears to contain a typo, as 38/39 does not equal 88.9%.

2.The response indicates that confidence intervals were added to Extended Data Figure 1a, but we were unable to locate them in the revised figure.

3.There remains a typo in Extended Data Figure 7: “Choa1” vs “Chao1.”

Reviewer #4

(Remarks to the Author)
I co-reviewed this manuscript with one of the reviewers who provided the listed reports. This is part of the Nature Communications initiative to facilitate training in peer review and to provide appropriate recognition for Early Career Researchers who co-review manuscripts.

**Open Access** This Peer Review File is licensed under a Creative Commons Attribution 4.0 International License, which permits use, sharing, adaptation, distribution and reproduction in any medium or format, as long as you give appropriate credit to the original author(s) and the source, provide a link to the Creative Commons license, and indicate if changes were made.
In cases where reviewers are anonymous, credit should be given to 'Anonymous Referee' and the source.
The images or other third party material in this Peer Review File are included in the article’s Creative Commons license, unless indicated otherwise in a credit line to the material. If material is not included in the article’s Creative Commons license and your intended use is not permitted by statutory regulation or exceeds the permitted use, you will need to obtain permission directly from the copyright holder.
To view a copy of this license, visit https://creativecommons.org/licenses/by/4.0/

**Response to Reviewers**

*Original manuscript title: Neoadjuvant Pepinemab Enhances Immune Checkpoint Blockade in Patients with Metastatic Melanoma*

**Revised manuscript title: Neoadjuvant Pepinemab with Immune Checkpoint Blockade Remodels the Tumor Microenvironment in Metastatic Melanoma**

Corresponding Author: Gregory B. Lesinski, Chrystal M. Paulos, Michael C. Lowe

We thank the Reviewers for their careful evaluation of our manuscript and for recognizing the importance of this translational study in neoadjuvant melanoma. We took the reviewers' concerns seriously and have revised the manuscript to more clearly present this work as a pilot biomarker-driven, hypothesis-generating trial rather than a definitive comparative efficacy study. Across the revised manuscript, we have tempered clinical claims, clarified the exploratory nature of cross-arm analyses, expanded discussion of limitations, added assay-specific denominators, incorporated confidence intervals where appropriate, and reframed the external MD Anderson cohort as contextual rather than comparative.

We believe these revisions substantially strengthen the manuscript while preserving its central contribution: a biologically rich, multi-platform analysis of how pepinemab-containing neoadjuvant regimens are associated with remodeling of the tumor microenvironment in metastatic melanoma. These data provide important translational insight into SEMA4D blockade in combination with immune checkpoint inhibition and offer a clear rationale for future randomized evaluation.

Below, we address each comment point by point. Reviewer comments are reproduced in ***italics***, and our responses are provided in **plain text**. Changes made to the manuscript are highlighted in cyan throughout the manuscript.

**Reviewer #1**

*This is a pilot study that evaluated pepinemab, a Semaphorin 4D (SEMA4D)–blocking antibody, in combination with nivolumab and/or ipilimumab in a neoadjuvant trial for patients with resectable melanoma stage IIIB/C/D melanoma. Patients received two neoadjuvant doses followed by surgery on day 42 and one year of adjuvant nivolumab. Patients were sequentially enrolled into one of four treatment cohorts (n=8 each): pepinemab/nivolumab, pepinemab/ipilimumab, pepinemab/nivolumab/ipilimumab, or nivolumab alone as well one arm of surgery alone (n = 6). pCR/MPR rates were 25%/37.5%, 12.5%/12.5%, 62.5%/75%, 25%/37.5% with pep/nivo, pep/ipi, pep/nivo/ipi and nivo alone respectively. Mechanistically, biomarker analysis of patient tumors reported significant immunomodulatory changes with pepinemab in combination with ICI. Overall, the study results support the safety, feasibility, and immune modulatory activity of pepinemab when administered in melanoma in the neoadjuvant setting together with ICI.*

**Comment 1.1**. *The study is interesting as a safety, feasibility and immune monitoring study but is limited by the too ambitious design of 5 study arms with small sample size in each, asking too many questions and making conclusive results hard to attain.*

**Response**: We agree with the reviewer that the sample size within each treatment limits the strength of conclusions that can be drawn, particularly for secondary clinical endpoints such as pathological response and recurrence-free survival. As this study was designed primarily as an exploratory-biomarker driven investigation, we have revised the manuscript to place greater emphasis on the translational and immune profiling findings, while more cautiously interpreting exploratory clinical outcomes and tempering conclusions throughout.

Importantly, the clinical endpoints discussed were pre-specified secondary objectives defined prospectively in the study protocol. While we acknowledge the limitations associated with the cohort size and multi-arm design, we believe these analyses remain valuable to report when interpreted appropriately as exploratory observations. To better align the manuscript with the original intent of the trial, we also revised the manuscript title to "*Neoadjuvant Pepinemab with Immune Checkpoint Blockade Remodels the Tumor Microenvironment in Metastatic Melanoma*" and replaced references to a "phase II trial" with "pilot biomarker-driven trial" throughout the manuscript.

**Comment 1.2**. *For example, what is the recommended next step in testing pepinemab in the neoadjuvant setting of melanoma?*

**Response**: We thank the reviewer for this question. The findings from the pilot biomarker-driven study imply that pepinemab-containing regimens are associated with immunomodulation in both the tumor and peripheral blood, while remaining feasible and tolerate in the neoadjuvant setting. Given these observations, an appropriate next step would be a prospective randomized, phase II clinical trial evaluating dual blockade of PD-1 and CTLA-4, with or without pepinemab in patients with resectable melanoma. Such a study would enable more definitive assessment of the biologic and clinical contribution of SEMA4D blockade in combination with immune checkpoint inhibition. We revised the Discussion to better outline these future directions.

**Comment 1.3**. *The pCR/MPR rates were identical between the pepinemab/nivolumab arm and nivolumab alone arm. Does that mean that pepinemab has no added value to PD1 blockade?*

**Response**: We agree that the cohort size in each treatment arm limits interpretation of secondary clinical outcomes, such as pCR/MPR, and the study was not powered for definitive efficacy comparisons between groups. Accordingly, we have toned down conclusions throughout the manuscript regarding clinical outcomes. However, despite similar pCR/MPR rates in these small cohorts, correlative analyses showed biological differences between treatment groups, including changes in immune biomarkers and tumor microenvironment organization associated with pepinemab-containing regiments (see Figures 2b, 3d,e, 4b). These findings are biologically meaningful and support further evaluation of SEMA4D blockade in larger, appropriately powered studies.

**Comment 1.4**. *What is the value of the surgery alone arm as part of this study?*

**Response**: At the time this trial began enrollment in 2018, neoadjuvant PD-1 pathway blockade was not yet established as the standard of care treatment for patients with resectable stage III melanoma. Patients enrolled to the surgery-only cohort were individuals who elected to proceed directly to surgery, rather than receive neoadjuvant therapy. Because the primary objective of this study was biomarker-driven and tumor microenvironment analysis, inclusion of a surgery-only arm also provided useful reference groups for interpretation of treatment-associated immune changes, particularly given the limited availability of pre-treatment biopsy tissue suitable for comprehensive special analyses. We have clarified this rationale in the revised manuscript.

**Comment 1.5**. *Therefore, it may have been more interesting to evaluate 2 or 3 study arms allowing more definitive analyses supporting more conclusive recommendations and future directions.*

**Response**: We agree with the reviewer that a study design with fewer treatment arms could have increased sample size per group and improved power for exploratory clinical comparisons. However, both ipilimumab and nivolumab represent clinically relevant checkpoint inhibitors with distinct mechanisms of action, making evaluations of their respective combinations with pepinemab of biological interest. The study was therefore designed primarily to explore treatment-associated changes within the tumor microenvironment across different checkpoint blockade backbones, based on supportive preclinical data showing significantly improved tumor control with both PD-1/SEMA4D and CTLA-4/SEMA4D inhibitor combinations compared to SEMA4D inhibition alone or control. As discussed above, we now more clearly frame this work as a pilot biomarker-driven study and have tempered conclusions related to explorative clinical outcomes throughout the manuscript. This primary objective was indeed accomplished with the study design. As noted above, these correlative data support a subsequent randomized clinical trial to evaluate ipilimumab/nivolumab +/- pepinemab powered for clinical efficacy. We have acknowledged in the Discussion that sample size is noted as a limitation and outlined the next logical steps.

**Comment 2.0.** *The study also lacked a pepinemab monotherapy arm which would have been ideal to better understand the added impact of pepinemab to ICI. Alternatively, would have been useful to initiate systemic therapy with one dose of pepinemab while initiating ICI a week or 2 later while obtaining biopsies at baseline, then right before ICI addition and then while on-treatment with the combination.*

**Response**: We agree that inclusion of a pepinemab monotherapy arm and additional serial biopsies could have provided further mechanistic insight into the independent biologic effects of SEMA4D blockade. However, the study design was developed with consideration of both ethical and logistical constraints in patients with resectable stage III melanoma. We believe this study design was appropriate to meet the primary correlative objectives of the clinical trial. Much like

Relatlimab (an FDA-approved agent for melanoma), prior preclinical and early phase clinical trials in patients with other malignancies suggested limited single-agent efficacy of pepinemab, while supporting its use in combination with immune checkpoint blockade therapy through effects on lymphocyte infiltration and myeloid remodeling in the oppressive tumor. In addition, clinical activity of pepinemab-containing combinations has been observed in patients with non-small cell lung cancer. At the time of opening the trial there were also emerging data indicating immune checkpoint inhibitors may offer benefit in the neoadjuvant setting, thus we did not elect to withhold these therapies in patients in order to administer pepinemab alone, or stagger treatment initiation.

**Comment 3.0**. *The lack of randomization is also a limitation in order to minimize confounding factors that may impact the clinical as well as the mechanistic analyses that can be attributed to sequential enrollment spanning over a number of years.*

**Response**: We agree with the reviewer that the lack of concurrent randomization represents an important limitation of this study. It may introduce the potential for confounding related to sequential enrollment over time. Thus, we have revised the Discussion to explicitly acknowledge these considerations and to clarify that comparisons across treatment groups should be interpreted cautiously, particularly in exploring clinical endpoints. However, as this clinical trial was not designed in a manner to evaluate efficacy as a primary endpoint, we determined randomization was not necessary and added little to a study powered for translational endpoints. During this clinical trial, the management of patients enrolled also did not change, thus we are confident in the integrity of our patient population and data derived from this study.

**Comment 4.0**. *The authors note that no patients experienced toxicity that required delay or discontinuation of therapy or planned surgery. What were the range and median duration of time from study registration to surgery that was planned at day 42?*

**Response**: Thank you for the opportunity to clarify this information. As specified in the clinical protocol, all surgeries were required to occur on day 42 +/- 7 days from enrollment; and no patient underwent surgery outside this window. The observed range was day 37-45 days, with a median of 43 days from registration to surgery.

**Comment 5.1**. *Mechanistically, the authors note that pepinemab plus ICI induced pathologic responses associated with immune remodeling, including increased T and B cell infiltration and enrichment of type 1 conventional dendritic cells. These changes promoted formation of mature lymphoid aggregates resembling tertiary lymphoid structures that harbored clonally expanded T cell receptor repertoires, consistent with localized cytotoxic activity. Aren't such changes expected in pathologic responses following anti-PD1 as monotherapy or anti-PD1 + anti-CTLA4?*

**Response**: We agree that immune changes can occur with anti–PD-1 or anti–PD-1 plus anti–CTLA-4 therapy alone. To address this point, we now more explicitly highlight data supporting a myeloid-directed effect associated with pepinemab. Specifically, Figure 3b-e demonstrates APC alterations that were not observed with ipilimumab plus nivolumab alone, suggesting effects not

fully explained by checkpoint blockade therapy. In addition, we cautiously examined the MD Anderson dataset for supportive trends and present the Emory and MD Anderson cohorts separately to avoid overinterpretation, while still allowing conceptual comparison. These analyses primarily emphasize changes within the myeloid compartment. Differences in lymphoid aggregate maturity are addressed separately in the subsequent figure.

**Comment 5.2**. *It is not clear how the study proves the added impact of pepinemab in mediating these immunomodulatory changes within the TME as compared to PD1 blockade alone or in combination with CTLA4 blockade.*

**Response**: We thank the reviewer for this comment and refer to our response above (Comment 1.9), where we clarify the evidence supporting a pepinemab-associated myeloid/APC effect beyond checkpoint blockade alone.

**Comment 6.0**. *The authors note that "Melanoma clinical stage was 15% stage IIIB, 46% IIIC and 38% IIID". This adds up to 99%.*

**Response**: Thank you for catching this discrepancy. We reviewed the raw clinical data and concluded this reflects a rounding issue. The percentages have been corrected in the revised manuscript to ensure they sum appropriately.

**Comment 7.0**. *Similarly, the authors note that "46% of patients had de novo presentation and 51% had prior surgical management of their primary disease". These add up to 97%. What about the other 3%.*

**Response**: Thank you for identifying this discrepancy. We reviewed the underlying clinical dataset and corrected the percentages in the revised manuscript.

**Comment 8.0**. *Toxicity is only presented briefly. A table that summarizes toxicities by grade should be included.*

**Response:** Thank you for the suggestion. We agree and have added a table summarizing grade-specific toxicities. This information can now be found as Supplemental Table 4 of the revised manuscript.

**Comment 9.0**. *The authors report the study as a randomized phase II trial, but in the study protocol it is labelled as a pilot study.*

**Response**: Thank you for pointing out this inconsistency. We are not aware of any instance in the submitted manuscript in which the study was described as randomized, as the Methods section states that this was a sequentially enrolling clinical trial. However, we acknowledge

inconsistent terminology regarding study designation across the manuscript, protocol, and ClinicalTrials.gov registration. To improve clarity and consistency, we have revised all references throughout the manuscript to uniformly describe this trial as a "pilot biomarker-driven study," consistent with the clinical protocol and trial registration.

**Reviewer #2**

*In their manuscript 'Neoadjuvant Pepinemab Enhances Immune Checkpoint Blockade in Patients with Metastatic Melanoma' the authors describe a small phase 1b trial testing a SEMA4D inhibitor in combination with neoadjuvant nivolumab or nivolumab plus ipilimumab in macroscopic stage III melanoma and present here in addition the translational research work.*

**Overall Comment:** *While being an interesting concept the authors ignore completely the GCP of reporting on small cohorts and over-interpret their data completely. The whole manuscript must be adjusted in toning done the wording in regards of claiming efficacy and enhancement.*

**Response**: We appreciate the reviewer's point concerning interpretation of data from relatively small treatment cohorts. In response, we have revised the manuscript throughout to adopt more conservative language and to avoid overinterpretation of the findings. Now, we place greater emphasis on the biomarker analyses, which represented the primary objective of this pilot biomarker-driven study. In addition, all conclusions related to clinical efficacy or immune enhancement have been carefully revised to clarify that these observations are exploratory and hypothesis-generating in nature given the limited sample sizes within each treatment group.

**Major Comments**

**Comment 1.0**. *Introduction claims that many patients who initially benefit still progress. This is not true in the neoadjuvant setting*

**Response**: We appreciate the reviewer's clarification regarding terminology. While the proportion of patients varies by setting, recurrence after excellent pathologic response is still observed in a meaningful subset of cases. We have therefore revised "many" to "some" to align with a more conservative phrasing, and we retain the statement to emphasize that this remains an active area of investigation.

**Comment 2.0**. *Key resistance mediators described in the introduction have not been shown in the neoadjuvant setting - thus you need to adjust the wording*

**Response**: We thank the reviewer for highlighting this important distinction. The wording in the Introduction has been revised to accurately reflect the current evidence regarding these resistance mediators in the neoadjuvant setting and to avoid overstating conclusions beyond the available data.

**Comment 3.0**. *With 62.5% grade 3/4 tax in the triplet arm and 37.5% in the doublet arm, which is twice the grade 3/4 too as compared to trAE grade 3/4 in NADINA (30%) and SWOG1801 (15%). So how come you to the conclusion that this is well tolerated?*

**Response**: We appreciate the opportunity to clarify interpretation of the toxicity data. First, we note that the overall reported grade 3/4 treatment-related adverse event rate in the NADINA trial was 47.2%, rather than 30%. We agree that the frequency of grade 3 adverse events in the triplet arm of our study warrants careful interpretation. However, comparisons across studies are challenging given the very small cohort size in the present pilot biomarker-driven trial relative to the substantially larger randomized studies such as NADINA and SWOG1801. In the present study, there were only n=8 patients in each treatment arm. In contrast, the NADINA trial had an n=450 patients randomized between two treatment arms. In our study, each treatment arm included only eight patients, such that adverse event percentages are highly sensitive to small changes in event number. Importantly, despite the occurrence of grade 3 toxicities, no patients experienced grade 4 or 5 toxicities, no surgeries were delayed or cancelled due to treatment-related adverse events, and the observed toxicities were consistent with those expected for checkpoint inhibitor–based regimens. We have revised the manuscript to present these findings more cautiously and to avoid overinterpretation of tolerability comparisons across trials.

**Comment 4.0**. *You cannot conclude that the response was highest in the pepinemab containing arms, as the trial is clearly underpowered (as you state yourself) for comparisons*

**Response**: We agree with the reviewer. We have revised the manuscript accordingly. Definitive statements regarding comparative clinical efficacy between treatment arms have been removed *or* tempered throughout the manuscript. While these clinical endpoints were prospectively defined secondary objectives of the study, they are now presented more cautiously and interpreted as exploratory observations within the context of this pilot biomarker-driven trial.

**Comment 5.0**. *The arms have not been stratified for stage which makes any comparative clinical response conclusions in these very imbalanced cohorts absolutely unacceptable. At best case you can word this as giving some indication for a potential efficacy and really need to mention this in the results section and discuss in the discussion.*

**Response**: We thank the reviewer for highlighting this important limitation. We agree that the lack of stratification and small cohort sizes limit interpretation of comparative clinical outcomes across treatment arms. Accordingly, we have revised both the Results and Discussion sections to more clearly present these findings as exploratory observations that may suggest potential biologic and clinical activity, but which require validation in larger, appropriately powered randomized studies.

**Comment 6.0**. *the baseline characteristics of the ipi/nivo cohort are missing*

**Response:** We have now included a supplemental table (Extended table 1b) that contains the baseline characteristics of the ipi/nivo cohort.

**Comment 7.0**. *why have you not also analysed the nivo cohort from MDA which would have included at least one treatment scheme in both cohorts*

**Response:** Thank you for this excellent suggestion. We have now incorporated analysis of the nivolumab only cohort from MD Anderson, which is included in the revised plots (Fig 3d-f). This addition provides a treatment arm common to both cohorts and improves contextual interpretation across datasets.

**Comment 8.0**. *all TR analyses must compare nivolumab versus nivo+pep and ipilimumab+nivolumab vs ipi+nivo+pep and only in case of statistical significance you can speak of increase/superiore, etc. E.g. the conclusions in Fig 2b are completely overstated with the ipi+nivo cohort missing. All observations here could be also due to the addition of ipi.*

**Response**: As noted below by Reviewer 3, broad comparisons of data from our clinical trial to that from our MD Anderson-derived synthetic control cohort has major limitations. The primary purpose for including these samples in the current study was to better understand possible biologic differences between lymphoid aggregate (LA) structures in patient tumors following exposure to different immunotherapy treatment regimens. As such, the data for this synthetic control is limited to the IHC figures: 3, 4 and 5. Consistent with the narrative raised by all reviewers, this approach to discussing the synthetic arm seems an appropriate and more conservative approach aligned with the intent of this as a biomarker study. Thus, although perhaps interesting and tempting to conduct more exploratory comparisons between the ipi+nivo vs. triplet therapy arms, we are hesitant to do so for all the reasons stated. We do agree with the Reviewer regarding the conclusions for Figure 2b and have modified our interpretation, accordingly, acknowledging in our revised Discussion that some biologic differences could have been influenced by ipilimumab where appropriate.

**Comment 9.0**. *Figure 2d is illustrative. If you draw any conclusion you need to quantify for all patients*

**Response**: We apologize for omitting the compiled data for this figure. We agree that the data are stronger when presenting the aggregate. As a result, SEMA4D, CD8, CD4 and CD20 cell counts have now been calculated for each patient in the nivo and nivo+pepi cohorts within the periphery (1000 µm interior edge of tumor bed) and the tumor bed core (Tumor bed minus the 1000 µm periphery). The ratio was calculated as # cells within the Core / # cells within the periphery. The plots on the right now also show confidence intervals (CI) as suggested by reviewers and not p values, due to the low n. These compiled data are below and they show a trend of an increased ratio of CD8, and SEMA4D+ cells within the core of tumors from patients receiving pepinemab and nivolumab in comparison with nivolumab alone. The new data is found in Fig. 3a and is also pasted below for ease of review.

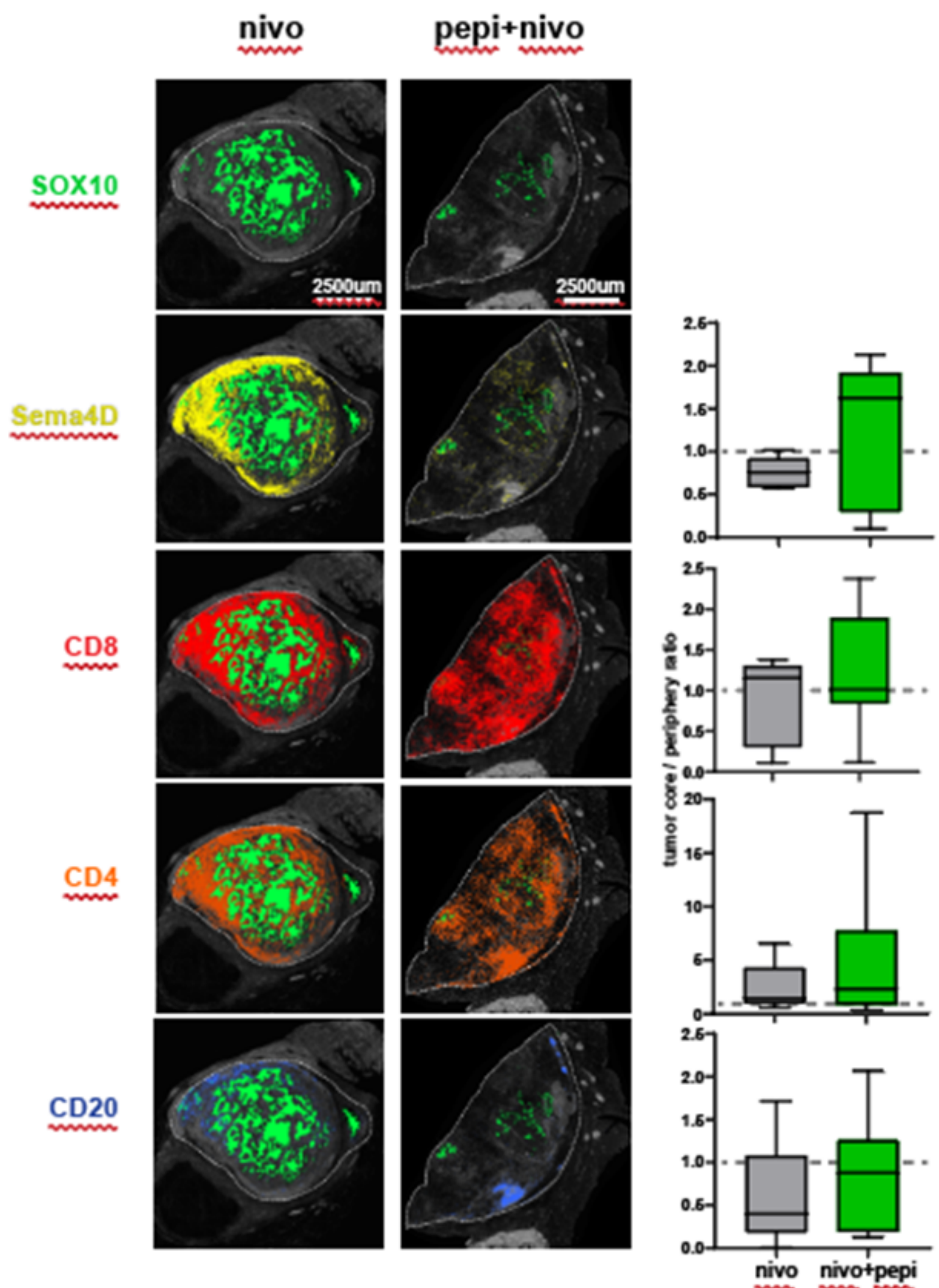


**Comment 10.0**. *Figure 3b only triplet versus nivo is significant. How can you conclude that the addition of pep does the effect?*

**Response**: We agree with the reviewer that interpretation of these analyses is limited by cohort size and variability between samples. In the revised manuscript, we have tempered conclusions regarding lymphoid aggregate abundance and no longer attribute these findings solely to the addition of pepinemab. As noted by the reviewer, only the comparison between the triplet therapy and nivolumab-alone cohorts reached statistical significance, while substantial variability was observed within the nivolumab/ipilimumab cohort. Thus, we revised the text to more cautiously describe these findings as associative observations within this exploratory biomarker study. Also, we have now refocused interpretation toward the more consistent treatment-associated changes observed in immune organization, lymphoid aggregate maturation, and myeloid remodeling, rather than overall lymphoid aggregate quantity alone.

**Comment 11.0.** *adjust all TR analyses and figure descriptions according to the suggestions above*

**Response**: We agree with the reviewer. We have revised the tissue response analyses, corresponding figure legends, and interpretive language throughout the manuscript accordingly. The revised analyses now emphasize pairwise, treatment-matched comparisons and a more conservative interpretation of findings. In addition, aside from the exploratory lymphoid aggregate analyses presented in Figures 4–5, we have clearly labeled / separated comparisons involving the external MD Anderson cohort to reduce the risk of overinterpretation.

**Minor Comments**

**Comment 1.0.** *Change the title stating that it is tested in the neoadjuvant setting, remove the enhance and use a more neutral wording*

**Response:** We agree with the reviewer. We have revised the title to more appropriately reflect the exploratory and biomarker-focused nature of the study. The revised title is: "Neoadjuvant Pepinemab with Immune Checkpoint Blockade Remodels the Tumor Microenvironment in Metastatic Melanoma."

**Comment 2.0.** *In the introduction the current efficacy data on neoadjuvant pembrolizumab (SWOG1801), ipilimumab + nivolumab (NADINA) and nivo/rela are missing.*

**Response:** Thank you for pointing this out. The Introduction has been revised to incorporate these efficacy data from prior larger trials.

**Comment 3.0.** *please describe in the introduction more clearly for a non expert where SEMA4D in expressed and functions as ligand for its receptors expressed on which immune cells at which stage.*
**Response:** Thank you for the suggestion. These fundamental properties of SEMA4D and its expression are now described in the revised Introduction.

**Reviewer #3**

We are encouraged that the reviewer states this study addresses an important translational question in neoadjuvant melanoma and presents biologically rich, multi-platform correlative data with potential impact

**Overall Comment**. *The manuscript reports results from a pilot integrated biomarker study in which 38 patients with resectable metastatic melanoma were assigned to five treatment arms. The study addresses an important translational question in neoadjuvant melanoma and presents biologically rich, multi platform correlative data with potential impact. However, the current manuscript overinterprets certain causal and comparative findings given the small, non*

*randomized cohorts; the absence of a contemporaneous nivolumab+ipilimumab control arm; the reliance on an external "synthetic control"; and the exploratory nature of many correlative analyses. The manuscript would benefit substantially from a more disciplined framing as a biomarker focused, hypothesis generating trial, accompanied by clearer reporting of patient flow and analysis denominators. More specifically,*

**Response:** We agree with the reviewer and appreciate these suggestions. In response, we substantially revised the manuscript to more clearly frame this work as a pilot biomarker-driven, hypothesis-generating study focused primarily on translational and tumor microenvironment endpoints. Throughout the revised manuscript, we have now tempered causal and comparative interpretations. We have clarified the exploratory nature of the correlative analysis, and acknowledged limitations related to cohort size, lack of concurrent randomization, and the use of external comparator cohorts. Also, we revised the Results, Discussion and figure legends to provide clearer reporting of patient flow, treatment comparisons, and analysis denominators where appropriate.

**Comment 1.0**. *The protocol and statistical plan describe this as a pilot integrated biomarker study with hypothesis generating objectives, yet portions of the manuscript imply that the trial supports comparative clinical efficacy conclusions. The manuscript should consistently differentiate biomarker endpoints from exploratory clinical outcomes and avoid language suggesting definitive comparative benefit.*

**Response:** We revised the manuscript to represent this work to readership as a biomarker study. As noted in our response to the other reviewers, we have also adopted a more disciplined set of conclusions from our secondary endpoints dealing with pathologic response and relapse free survival. More appropriately, we now treat these results as hypothesis generating. At the same time, because these clinical endpoints were prospectively defined secondary objectives of the study in the clinical trial, we believe it remains important to report them, but with appropriate context and interpretation. Collectively, the biomarker and tumor microenvironment findings from this study support further evaluation of pepinemab-containing regimens in larger, appropriately powered randomized trials.

**Comment 2.0.** *The manuscript acknowledges that randomization across all five arms was not feasible. Expanded discussion is needed on the resulting risks of temporal drift, confounding, and arm imbalance, particularly in the context of cross arm comparisons.*

**Response:** We agree with the reviewer that the inability to randomize concurrently across all five treatment arms introduces important limitations. These limitations include the potential for temporal drift, confounding, and imbalance in patient characteristics across cohorts. We have substantially expanded the Discussion section to more explicitly acknowledge these risks and to clarify the intended interpretation of the study findings.

Specifically, because enrollment into the treatment cohorts was not fully concurrent, changes over time in referral patterns, patient selection, surgical management, supportive care, or other

unmeasured clinical factors may have influenced observed differences between groups independent of treatment effect. Accordingly, cross-arm comparisons are now presented more cautiously and interpreted as exploratory rather than definitive.

Importantly, this study was designed primarily as a pilot integrated biomarker-driven investigation focused on biologic and tumor microenvironment changes associated with pepinemab-containing regimens rather than as a formally powered comparative efficacy trial. Also, we revised the manuscript throughout to better distinguish biomarker endpoints from exploratory clinical observations.

To further strengthen the statistical framing and interpretation of these limitations, Dr. Jeffrey M. Switchenko provided additional statistical review and contributed substantially to the revised discussion and interpretation of study limitations. Accordingly, Dr. Switchenko has been added as a co-author on this revised manuscript.

**Comment 3.0.** *The absence of a contemporaneous nivolumab+ipilimumab control arm is a major design limitation. Use of the external MD Anderson "synthetic control" should be characterized strictly as contextual or supportive rather than comparative evidence. Additional detail is needed on how batch or cohort effects were handled.*

**Response:** We agree with the reviewer that the absence of a contemporaneous nivolumab+ipilimumab control arm represents a limitation of this study. We also agree that the external MD Anderson cohort should be interpreted as contextual and supportive rather than as a definitive comparative control cohort. Therefore, we revised the manuscript throughout to clarify that these analyses were intended primarily to provide biologic context for understanding lymphoid aggregate features across distinct immunotherapy regimens, rather than to support direct efficacy comparisons between independent clinical trials.

To minimize technical variability, samples from both cohorts were processed, stained, and analyzed using the same multiplex immunohistochemistry and digital spatial profiling workflows, reagents, image analysis pipelines, and scoring criteria. In addition, the Emory and MD Anderson cohorts were analyzed separately where appropriate, and interpretations involving cross-cohort comparisons were substantially tempered in our revised manuscript to reduce the risk of overinterpretation related to cohort or batch effects. With these considerations in mind, we believe the revised presentation represents the most conservative and appropriate interpretation of these exploratory biomarker analyses.

**Comment 4.0.** *The manuscript notes that 38 patients were enrolled and 32 treated, but it remains difficult to track how many patients contributed to each correlative analysis, each survival analysis, and each comparison set. A CONSORT style flow diagram and a table summarizing assay specific denominators by arm would improve transparency.*

**Response:** Thank you for the opportunity to clarify our study population and provide transparency on how each patient contributed to the correlative analysis. As requested, we made a CONSORT

style flow diagram to outline this information. This is now provided as Extended data Figure 2 in the revised manuscript. A table identifying individual assay denominators by figure has been added to the Supplementary data (Supplementary table 3).

**Comment 5.0.** *Overstatements in the discussion should be tempered. Phrases implying that pepinemab "improves" clinical benefit or that the data demonstrate distinct contributions beyond dual ICI should be revised to more cautious language such as "is associated with," "supports the hypothesis," or "warrants evaluation in a larger trial."*

**Response:** The Discussion has been revised to remove and/or revise these statements in a manner that is more conservative.

**Comment 6.0.** *For figure panels relying on ordinary ANOVA or Fisher's exact tests in very small cohorts, effect sizes and confidence intervals would be more informative than isolated P values.*

**Response:** Thank you for the input. As noted above in our response to Reviewer 2, we have replaced all the p values on figures with confidence intervals.

**Minor revisions:**

**Comment 1.0.** *For all main and extended figures, include the exact n per arm and per assay in each legend.*

**Response:** This information has been added to the figures and legends as appropriate in both main and extended figures. To keep figures and legends from being too cluttered, some legends refer to the consort diagram (Extended data fig 2) and/or assay denominator table (supplementary table 3).

**Comment 2.0.** *Follow up time for recurrence analyses should be reported explicitly (median follow up, range, and censoring pattern).*

**Response:** We have now included the median follow up, range and censoring pattern. Patients without recurrence were censored at the date of last documented follow-up or death.

**Comment 3.0.** *Page 7: "based on" appears twice in the sentence beginning "A more comprehensive multiplexed IHC analysis…".*

**Response:** Our apologies for the typo. This has been corrected.

**Comment 4.0.** *Add confidence intervals to Figure 1b.*

**Response:** Confidence intervals have been added to now Figure (Ext fig 1a).

**Comment 5.0.** *Include the surgery only arm in Figure 1d.*

**Response:** This comment refers to prior Kaplan-Meier analyses that have since been removed from the manuscript to ensure we are not over-interpreting the secondary endpoints from the trial. Thus it is no longer a comment that needs addressed.

**Comment 6.0.** *The number of samples described in the legend does not match the number of plotted data points in Figure 2b. Missing data in the "pepi + ipi" group may explain fewer observations, but the appearance of more plotted lines than specified (e.g., nine in "nivo" when n=8; seven in "pepi + nivo" when n=5) requires clarification.*

**Response:** To clarify, in the Emory nivo cohort, one subject had been enrolled on study that ended up not meeting criteria and was replaced. This subject was mistakenly included in the nivo cohort but has been rectified using the correct 8 patients. Upon further inspection, the pepi+ipi and pepi+ nivo plots were reversed explaining the numbers not matching. All plots have been thoroughly checked, and this was the only plot with errors.

**Comment 7.0.** *Clarify why the "nivo" group is omitted from Figure 5.*

**Response:** To clarify the omission of "nivo" group from figure 5, Only Grade 3 LA were included in analysis for figure 5 for direct comparison of equivalent LA maturity. Unfortunately, there were no grade 3 LA within the emory nivo group that were selected for GeoMx analysis.

**Comment 8.0.** *Extended Data Figure 5a may contain a typo in the y axis label.*

**Response:** Thank you for identifying this typo. This has been corrected.

**Comment 9.0.** *Extended Table 1 is missing a table title.*

**Response:** A title has been added to the Extended Table 1.

**Reviewer #4**

*I co-reviewed this manuscript with one of the reviewers who provided the listed reports. This is part of the Nature Communications initiative to facilitate training in peer review and to provide appropriate recognition for Early Career Researchers who co-review manuscripts.*

**Comment 1.0**. *No scientific comments provided.*

**Response**: We thank the reviewer for their participation in the peer-review process.

**Reviewer #2 (Remarks to the Author):**

**Comment**: *"The reviewers addressed all my concerns except for one:*

*The 30% and 15% grade 3/4 AEs are the reported systemic treatment related adverse events. The additional 17% you are referring to are the surgery related AEs. Considering the fact that you are reporting only the systemic therapy related AEs the statement must be that the triplet in your work doubles/quadruples the systemic therapy grade 3/4 toxicity as compared to ipi/nivo and pembro neoadj respectively.*

*This needs to be discussed because such a considerable more toxic therapy should be therefore considered only for patients with highly unfavorable markers unlikely to respond to the NADINA and SWOG schemes."*

**Response**: We appreciate this comment and the opportunity to clarify. The reviewer is referring to the treatment related grade3/4 AEs that occurred within the prior NADINA (30%) and SWOG1801 (15%) trials. To provide context, in the present study, we report 62.5% grade 3/4 treatment related AEs in the triplet arm (5/8 patients) and 37.5% (3/8 patients) in the doublet arm. Given the extremely small cohorts in this study (n=8/treatment arm) in comparison to NADINA (n=450 patients randomized across two arms) and SWOG1801 (n=313 patients in two arms), we do not feel it is appropriate to compare either toxicity or efficacy measures across clinical trials with definitive statements. This is consistent with the comments of all three referees during the initial review, and as such we are not comfortable making strong statements related to relative degrees of toxicity across clinical trials. We do agree with the reviewer that further efforts to identify optimal high-risk patient populations that may benefit from experimental neoadjuvant clinical trials are warranted. A statement reflecting this has been added to the revised discussion on line 372-374 and 383-385.

**Reviewer #3**

**General Comment:** *"Most of our prior comments have been fully addressed. However, we noted several inconsistencies between the authors' notes in the response file and the revised manuscript/supplemental files:"*

**Response**: We apologize for this oversight. Our group has worked extensively with the Editors to reconcile differences and have provided the journal with a revised manuscript and Table 1 that has corrected these issues.

**Comment 1.** *"We could not identify a table labeled as Extended Data Table 1. In addition, the clinical stage names are inconsistent between the paragraph and the table (IIIB–IIID versus IIIA–IIIC). The percentages reported for the clinical stages in the paragraph also do not fully align with the values shown in the table, for example, 15.4% versus 15.5% and 46.2% versus*

*46%. The total percentage reported below also appears to contain a typo, as 38/39 does not equal 88.9%."*
**Response**: Thank you for pointing this out.  As noted above, this was an error on our part, and we have corrected the information contained in Table 1 after careful review of the clinical source data, which has been provided to the editors.

**Comment 2**. *"The response indicates that confidence intervals were added to Extended Data Figure 1a, but we were unable to locate them in the revised figure."*
**Response**: Confidence intervals have been added to now Figure (Supplemental fig 1a).

**Comment 3**. "There remains a typo in Extended Data Figure 7: "Choa1" vs "Chao1."
**Response**: We apologize for the typo, which has been corrected.
